# Computational Design, Synthesis, and Biophysical Evaluation of β-Amido Boronic Acids as SARS-CoV-2 M^pro^ Inhibitors

**DOI:** 10.3390/molecules28052356

**Published:** 2023-03-03

**Authors:** Enrico M. A. Fassi, Marco Manenti, Andrea Citarella, Michele Dei Cas, Sara Casati, Nicola Micale, Tanja Schirmeister, Gabriella Roda, Alessandra Silvani, Giovanni Grazioso

**Affiliations:** 1 Dipartimento di Scienze Farmaceutiche, Università degli Studi di Milano, Via L. Mangiagalli 25, 20133 Milan, Italy; 2 Dipartimento di Chimica, Università degli Studi di Milano, Via Golgi 19, 20133 Milan, Italy; 3 Dipartimento di Scienze della Salute, Università degli Studi di Milano, Via a di Rudinì 8, 20142 Milan, Italy; 4 Dipartimento di Scienze Biomediche, Chirurgiche ed Odontoiatriche, Università degli Studi di Milano, Via Luigi Mangiagalli 37, 20133 Milan, Italy; 5 Dipartimento di Scienze Chimiche, Biologiche, Farmaceutiche ed Ambientali, Università degli Studi di Messina, Viale Ferdinando Stagno D’Alcontres 31, 98166 Messina, Italy; 6 Department of Medicinal Chemistry, Institute of Pharmaceutical and Biomedical Sciences, Johannes Gutenberg University, Staudinger Weg 5, 55128 Mainz, Germany

**Keywords:** drug design, MM-GBSA, boronic acids, multicomponent reactions, protease, SARS-CoV-2, M^pro^

## Abstract

The COVID-19 pandemic has given a strong impetus to the search for antivirals active on SARS-associated coronaviruses. Over these years, numerous vaccines have been developed and many of these are effective and clinically available. Similarly, small molecules and monoclonal antibodies have also been approved by the FDA and EMA for the treatment of SARS-CoV-2 infection in patients who could develop the severe form of COVID-19. Among the available therapeutic tools, the small molecule nirmatrelvir was approved in 2021. It is a drug capable of binding to the M^pro^ protease, an enzyme encoded by the viral genome and essential for viral intracellular replication. In this work, by virtual screening of a focused library of β-amido boronic acids, we have designed and synthesized a focused library of compounds. All of them were biophysically tested by microscale thermophoresis, attaining encouraging results. Moreover, they also displayed M^pro^ protease inhibitory activity, as demonstrated by performing enzymatic assays. We are confident that this study will pave the way for the design of new drugs potentially useful for the treatment of SARS-CoV-2 viral infection.

## 1. Introduction

**Figure 1:**
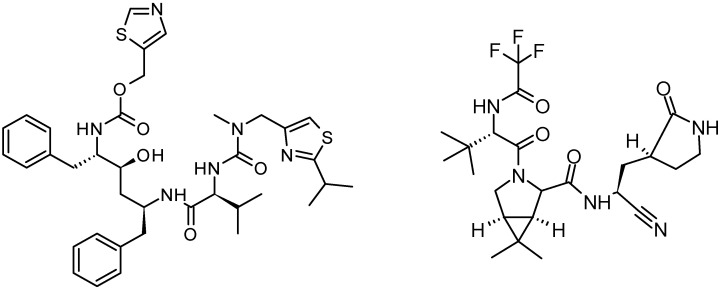
Chemical structure of Ritonavir (**left**) and Nirmatrelvir (**right**), both composing Paxlovid^®^, clinically employed for the treatment of COVID-19.

The COVID-19 pandemic caused by the SARS-CoV-2 virus continues to have a significant impact on the lives and economies of countries worldwide. To combat this pandemic, numerous pharmaceutical companies and academia have developed vaccines. The first vaccines were released at the end of 2020 by Pfizer, Moderna, and Astra Zeneca. Although these vaccines elicit an immune response to SARS-CoV-2 infection, unfortunately, they are ineffective against new and unpredicted mutations of the spike protein, which is the protein target of the triggered immune response. For this reason, in parallel with active research on new and more efficient vaccines, cheap and orally available drugs directed at molecular targets vital for the replication of the virus need to be developed. Among numerous viral molecular targets, the main protease (M^pro^), also called 3CLpro, is considered the most therapeutically relevant target, as it is highly conserved among β-coronaviruses and possesses very few structural similarities to human proteases [1,2]. Due to the important role that M^pro^ plays in the replication of human coronaviruses, in particular, that of SARS-CoV-2, finding a specific inhibitor of this enzyme that could be clinically useful is the focus of current research. During 2022, the FDA approved the association of ritonavir (an anti-HIV drug) and nirmatrelvir (Paxlovid^®^, Pfizer Inc., New York, NY, USA) for the treatment of severe COVID-19, highlighting the importance of including M^pro^ inhibitors in the therapeutic armamentarium against SARS-CoV-2 (Figure 1).

SARS-CoV-2 Mpro (Mpro^Cov-2^) is a homodimeric chymotrypsin-like cysteine protease, with a non-canonical catalytic dyad composed of Cys145 and His41, which forms hydrogen bonds with the water molecule needed to hydrolyze the amide bond of the substrates. Mpro^Cov-2^, together with SARS-CoV-2 papain-like protease (PL^pro^), contributes to viral polyprotein processing, acting in the early stages of viral replication inside infected cells. The inhibition of one or both of these enzymes constitutes a valuable therapeutic strategy to prevent SARS-CoV-2 proliferation in infected cells. Regarding the mechanism of action of nirmatrelvir, it covalently binds M^pro^-Cys145, which is responsible for the catalytic cleavage of polypeptide amide bonds, by means of a nitrile warhead. In this way, nirmatrelvir blocks the production of functional viral proteins needed to build structural proteins, such as those that form the viral capsid.

Numerous compounds bearing different warheads that show non-covalent or covalent activity against M^pro^ have been reported in the literature [3,4,5,6,7]. Among these, research suggests that boron-containing compounds (BCCs) may have potential against SARS-CoV-2 infection [8], similar to that of boronic acid derivatives reported by Bacha et al., which are active against human β-coronaviruses other than SARS-CoV [9]. BCCs have attracted increasing attention in recent years, with five BCCs now used as therapeutic agents, three of which having been approved in the last 6 years [10]. In terms of the clinical applications of BCCs, tavaborole (Kerdyn^®^, Pfizer Inc., New York, NY, USA) is used as a treatment for fungal infections, and bortezomib (Velcade^®^, Takeda Pharmaceuticals, Lexington, MA, USA) is used as an anti-cancer agent. Another BCC, vaborbactam (Vabomere^®^, Melinta Therapeutics, Parsippany, NJ, USA), in association with meropenem, inhibits β-lactamases produced by antibiotic-resistant bacterial infections [11]. More recently, the boron neutron capture therapy, by the irradiation of nonradioactive boron-10, is an emerging treatment modality for tumors [12].

Based on the considerations of the importance of BCCs as covalent inhibitors and of Mpro^CoV-2^ as a therapeutic target for COVID-19 treatment, we performed a computational study, including covalent docking, molecular dynamics (MD) simulations, and Molecular Mechanics-Generalized Born Surface Area (MM-GBSA) calculations, on a virtual library of compounds, all endowed with a boronic acid warhead. This study utilized a library of easily synthesizable β-amido boronic acids. Their chemical synthesis relies on multi-component methodologies, and they have advantages in terms of process speed, ease of work up, and use of commercial substrates compared to multi-step routes. By varying and modulating the chemical structure of the individual components, multi-component reactions allow access to peptidomimetic skeletons characterized by high levels of structural diversity. In this study, we synthesized a number of computationally optimized derivatives and then determined the affinities of these derivatives for recombinant Mpro^Cov-2^ through biophysical experiments. We then evaluated the capability of the most promising compounds to inhibit the catalytic activity of Mpro^Cov-2^.

## 2. Results and Discussion

### 2.1. Computational Design of New β-Amino Boronic Acids

**Figure 2:**
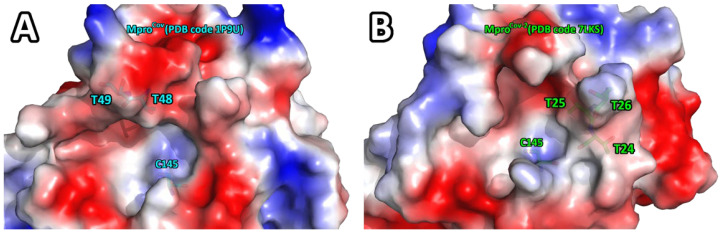
Three-dimensional depiction of the Mpro^CoV^ (**A**) and Mpro^CoV-2^ (**B**) catalytic sites. The enzyme surfaces are represented as a “solvent accessible surface”, in which the partial charges of the residue atoms are colored in blue or in red, accordingly with their positive or negative charge. Residues are numbered as in the X-ray structures.

Bacha et al. previously reported that bifunctional phenyl boronic acid compounds could bind to a cluster of serine residues (Ser139, Ser144, and Ser147) on Mpro^CoV-2^, the active sites of which are largely conserved among β-coronaviruses [9]. With the aim of designing new inhibitors targeting Mpro^Cov-2^, we structurally aligned the X-ray structures of both M^pro^ (PDB 1P9U [13] for Mpro^CoV^ and 7LKS [14] for Mpro^CoV-2^) and found that the majority of residues shaping the catalytic sites of both enzymes were largely conserved. Intriguingly, Mpro^CoV-2^ contains a peculiar Thr-rich cluster (Thr24-Thr25-Thr26) in place of the Ser-rich cluster found in Mpro^CoV^. Furthermore, among these three threonine residues, Thr25 projected its side chain in the catalytic site of the enzyme, and the Thr25 Cα atom was only 10.7 Å far from the one of Cys145, the residue targeted by most known Cys protease inhibitors, including nirmatrelvir (Figure 2).

**Scheme 1:**
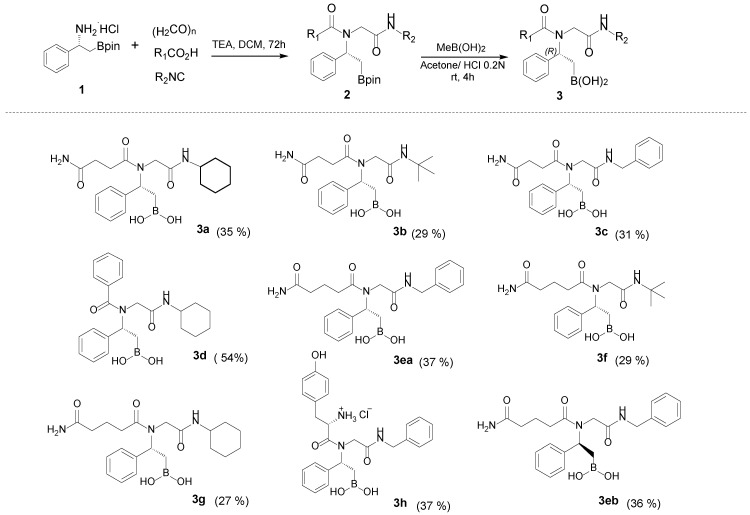
Synthesis of target β-amido boronic acids **3**, overall two-steps yield in brackets.

Accordingly, as proposed by Bacha et al., we used the Thr-rich cluster of Mpro^CoV-2^ as a target for the computational design of new boronic acids [9], creating a virtual library composed of β-amido boronic acids capable of covalently binding the side chain of Thr25. Relying on the Ugi reaction reported in Scheme 1, chemical diversity was introduced through 49 carboxylic acids, bearing different R_1_ groups, which were virtually combined with three isonitriles (benzyl, cyclohexyl, and *t*-butyl as R_2_, Table 1). Among the selected carboxylic acids, 29 were chosen among those already available in our organic synthesis laboratory, and the remaining 20 were natural amino acids. We then used the ‘enumeration’ tool of Maestro software (Schrödinger Inc., New York, NY, USA) to create a virtual library of 294 compounds containing both enantiomers of each compound. After performing covalent docking calculations, the three top-scoring binding poses of each ligand were simulated in complex with Mpro^CoV-2^ in 250 ns long MD simulations. The ligands displaying the lowest non-hydrogen atoms root mean square deviation (RMSD) over the simulation time were chosen for calculating ligand binding free energy (ΔG*) values, adopting the MM-GBSA single trajectory approach (see the Experimental section for details) [15,16,17]. The results obtained suggested that the compounds with the lowest ΔG* values (Table 1) possessed an *R* configuration at the chiral center and contained a 2-aminocarbonylethyl (**3a**, **3b**, **3c**) or 3-aminocarbonylpropyl (**3f**, **3ea**, **3eb**, **3g**) chain as an R_1_ substituent. Among the compounds derived from the natural amino acids, only the compound retaining the *L*-tyrosine side chain (**3h**) appeared to be promising as potent Mpro^CoV-2^ inhibitors.

**Figure 3:**
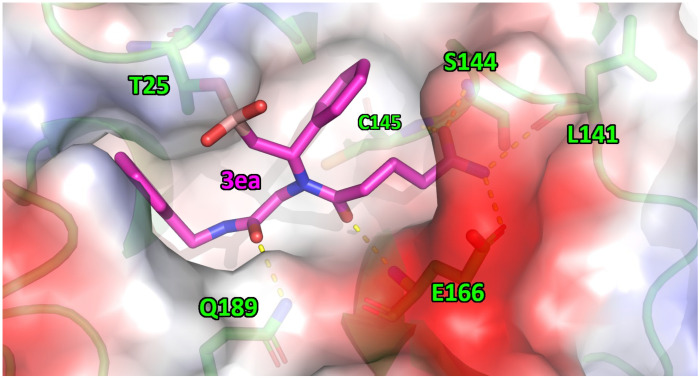
Supposed binding mode of **3ea** (magenta sticks) in complex with Mpro^CoV-2^. The enzyme surface is represented as a “solvent accessible surface”, in which the partial charges of the residue atoms are colored in blue or in red, accordingly with their positive or negative charge. The hydrogen bonds are represented as dashed yellow lines. Residues are numbered as was found in the X-ray structure.

As the most promising compounds contained 2-aminocarbonylethyl and 3-aminocarbonylpropyl as R_1_, we speculated that the number of methylene groups could be critical for the Mpro^CoV-2^ inhibitory activity. To establish whether this was the case, a compound derived using 3-amino-3-oxopropanoic acid as reactant, bearing only a methylene group between the amino boronic core and the primary amide group was designed. The ΔG* value of the resulting compound, simulated in complex with Mpro^CoV-2^, was higher than that of its homologs. Consequently, we can argue that effective (at least theoretically) inhibitors of Mpro^CoV-2^ depend critically on the full occupation of the Mpro^CoV-2^ pocket shaped by Leu141, Asn142, Gly143, Glu166, and His163 (Figure 3).

### 2.2. Chemistry

Based on the computational studies, the β-amido boronic acids reported in Table 1 were synthesized. Relying on our previous experience [18,19], the Ugi multi-component reaction (Ugi-4CR) was chosen as the key step for the synthesis of boron-enriched peptidomimetics. Starting from enantiopure β-amino boronic acid hydrochloride **1** and formaldehyde (fixed amino and carbonyl components, respectively), a series of selected acids and isocyanides were employed in the reaction. All compounds were easily obtained as pinacol esters **2**, which were purified by direct flash chromatography and then subjected to mild boron deprotection using methylboronic acid, resulting in target-free boronic acids **3** (Scheme 1).

The synthesis protocol proved to be suitable with 5-amino-5-oxopentanoic acid, 4-amino-4-oxobutanoic acid, and benzoic acid as acid components, in combination with commercial isocyanides (**3a**–**3g**). Boc-l-tyrosine was suitable for the reaction, giving the desired product **3h**, as a hydrochloride salt, with a good yield. Finally, as a control experiment in the biological evaluation, the *S*-enantiomer of compound **3ea**, namely, **3eb**, was also prepared.

All the obtained products were stable and were fully characterized by ^1^H, ^11^B, and ^13^C NMR and by high-resolution mass spectrometry. In most cases, the ^1^H and ^13^C spectra showed more rotamers, as expected in the presence of tertiary amide bonds in peptides and peptidomimetic compounds [20]. As such isomerism could not be solved by varying the polarity of deuterated solvents or running the experiments at higher temperature, the rotamers’ ratio was safely quantified by integration of the ^1^H NMR peaks assigned to each rotamer. As equilibrium between monomeric and trimeric forms of free boronic acids **3** is observed in deuterated solvents [21], one drop of water was added to the NMR solvent, in order to suppress intermolecular interactions (see Supplementary Materials).

### 2.3. Biophysical Assays by Microscale Thermophoresis (MST)

MST is a biophysical technique enabling the characterization and quantification of molecular interactions of two partners in liquid phase, without any sample immobilization. MST experiments aim at measuring ligand-dependent changes in the temperature-related intensity change (TRIC-F_norm_) as a function of the ligand concentration in a dose–response curve [22,23,24,25]. In the experiments conducted in this research, we used His-tagged recombinant Mpro^CoV-2^ provided by GeneTex. To determine the minimum concentration of ligand capable of covalently binding Mpro^CoV-2^, we used a standard protein labeling protocol, according to the manufacturer’s instructions (NanoTemper Technologies GmbH, München, Germany), using a constant concentration of fluorescent target molecule and fixed concentrations of ligands (Table 2).

**Figure 4:**
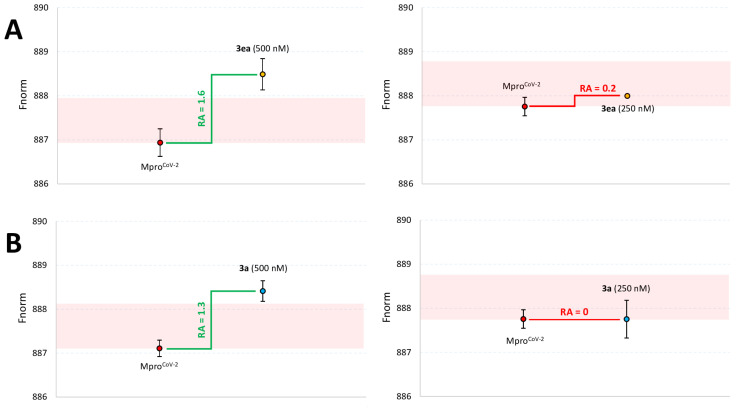
MST binding check experiments of (**A**) **3ea** and (**B**) **3a** tested at the concentration of 500 and 250 nM, respectively. In red is highlighted the “no binding” F_norm_ area due to an insufficient Response Amplitude (RA).

Except for **3h,** the obtained results suggested that all the synthesized compounds displayed significant affinity for the target, confirming the theoretical studies previously reported in the previous section. Moreover, as computationally predicted, the *S* enantiomer of compound **3ea** (named **3eb**) was less active than the *R* enantiomer (**3ea**). This β-amido boronic acid, together with **3a**, displayed the highest affinity for Mpro^CoV-2^, as binding was detected at concentrations between 250 and 500 nM (Figure 4). The MST binding check plots of all the compounds are shown in Figure S1 (Supplementary Materials).

To investigate the role played by the boronic acid warhead in the creation of the complex with the target, MST experiments on β-amido boronic compounds protected by the pinacol group (**3d-pin** and **3f-pin)** were also conducted (Table 2). Interestingly, although binding of **3d-pin** was not observed at a concentration of 100 µM, binding of free boronic acid (**3d**) was observed at concentrations ranging between 20 and 5 µM. A comparison of the binding check experiments of both **3d** and **3d-pin** at a concentration of 100 µM is shown in Figure S2A (Supplementary Materials). Similarly, the binding affinity of the pinacol analogue **3f-pin** was decreased compared to that of **3f** (Table 2 and Figure S2B,C, Supplementary Materials). These data suggest that the hydroxyl groups of the boronic warhead can induce critical bonds with the M^pro^ binding pocket. In fact, increasing the size of the warhead through the pinacol-protecting group prevented the entry of the ligand into the catalytic site. The low affinity displayed by pinacol ester could also be due to the impossibility of establishing a covalent bond with the target.

To verify that the β-amido boronic acid compounds bind only to the Mpro^CoV-2^ protein, and that they do not interfere with His-labeling or the dye, we conducted a binding affinity assay, testing **3ea** on the 6His peptide control provided by NanoTemper (NanoTemper Technologies GmbH, München, Germany), according to standard protocols suggested by MST developers (see Experimental Section). As shown in Figure S3 (Supplementary Materials), the MST traces do not display a binding curve, as was analyzed by “MO.Affinity Analysis” software provided by NanoTemper Technologies GmbH (München, Germany), and no significant fluorescence change is observed between the ligand samples in the capillaries, thus confirming the specific binding of compound **3ea** (and consequently that of all the other analogues) to the Mpro^CoV-2^ protein.

### 2.4. Inhibition of Mpro^CoV-2^: Reversible or Irreversible

We used MST to determine whether the binding of the β-amido boronic acid compounds was reversible or irreversible. First, we pre-incubated the Mpro^CoV-2^/**3ea** complex with a ligand concentration (Figure S4A, Supplementary Materials) that was previously determined high enough to bind the target in solution (100% bound, stock). We then measured the F_norm_ values of this and that of other solutions obtained progressively by diluting a Mpro^CoV-2^/**3ea** complex solution (stock). We then compared the F_norm_ of all the dilutions to that of the stock dilution. If the ligand was reversible, diluting the stock solution would produce a change in the F_norm_. Conversely, if the ligand was irreversible, no F_norm_ changes would be noted in the diluted solutions, as the ligand would be firmly bound to the target. In our experiments (Figure S4B, Supplementary Materials), the F_norm_ values did not change with the dilution of the stock complex solution, indicating that the ligand, once bound to the target, was unable to rapidly return in the solvent. This points to ligand binding being irreversible. In addition, preliminary LC-MS/MS experiments showed that at least 6% of the ligand present in solution could bind in an irreversible manner to Mpro^CoV-2^ (see Experimental section for details).

### 2.5. In Vitro Enzymatic Activity against Recombinant SARS-CoV-2 Proteases

We evaluated the ability of boronic acid derivatives **3a** and **3ea** to inhibit in vitro the activity of recombinant Mpro^CoV-2^ in a FRET-based assay (Table 3). First, we incubated the compounds with the Mpro^CoV-2^ enzyme for 10 min at 25 °C, followed by the addition of fluorogenic substrate. All the boronic acids partially reduced enzyme activity, with inhibition of ≅23% at a concentration of 20 μM. In contrast, evaluation of the enzymatic activity of **3a** and **3ae** against recombinant SARS-CoV-2 PL^pro^ revealed no inhibition, disclosing a noteworthy selectivity of the β-amido boronic acid compounds for Mpro^CoV-2^ with respect to PL^pro^. The selectivity of Mpro^CoV-2^ inhibitors against PL^pro^ represents an interesting feature, avoiding a specific interaction with human DUBs (Deubiquitinases), which share similarity sequences at the C-terminal with viral PL^pro^ [26]. Further extension of the incubation time to 30 min did not lead to an increase in percentage inhibition. Due to the low activity observed in the enzymatic inhibition assays, no further assays were conducted.

The results obtained by enzymatic assays, considered in conjunction with the results of the experiments using MST, in which some compounds displayed binding at a concentration of 500 nM, suggest that the studied compounds could bind to Mpro^CoV-2^ on a site different from the catalytic one. Varying the substrate concentration resulted in no significant changes in percentage inhibition, suggesting a non-competitive mode of target binding. Consequently, the existence and involvement of allosteric or multiple sites in the reaction with the studied compounds cannot be excluded. It is possible that due to its high reactivity, the boronic warhead could be attached to nucleophilic residues different from those in the Thr cluster. Alternatively, the ligands may not fully occupy the catalytic site of the enzyme, partially allowing the physiological activity of the Mpro^CoV-2^.

## 3. Experimental Section

### 3.1. Simulating System Setup and MD Simulations

The “protein preparation wizard” was used to build the Mpro^CoV-2^ computational model to be used as target, retrieving the coordinates from the Protein Data Bank (accession code 7LKS [14]). The virtual library of the amino boronic acids series was created by means of the “Reaction Based Enumeration” tool, implemented in Maestro (Schrödinger Inc., New York, NY, USA, release 2021-2). Then, the “ligprep” tool was used to generate the virtual library of compounds, containing both enantiomers of each compound, and to assign the OPLS4 force field. Docking calculations were accomplished by the GLIDE algorithm [27], supposing that a covalent bond was created between the side chain of Thr25 and the boronic warhead of the ligands composing the library. Subsequently, the ligand poses acquiring the lowest Gscore were simulated in complex with Mpro^CoV-2^ by 250 ns-long MD, evaluating also the RMSD/time plot (Figure S5, Supplementary Materials) to establish if the ligands remained anchored in the catalytic site of the enzyme. Finally, the ligands acquiring the most promising Gscore, and displaying the highest stability in the catalytic site, were analyzed in order to predict their ∆G values, by means of the PRIME tool of Maestro (Schrödinger Inc., New York, NY, USA). To this purpose, by a script developed by Schrodinger, the Thr25 was mutated into a Gly residue, leaving the ligand free in the Mpro^CoV-2^ catalytic site. Finally, the ligands binding free energy values (ΔG) were computed as the mean of the values acquired by the ligands in the trajectory frames in which they displayed the highest geometrical stability, as indicated by the RMSD/time plot. In these calculations, the single trajectory approach was applied, and the entropy contributions to the binding free energy, coming from the normal mode analysis, were neglected, due to the computational costs of the calculations and the inaccuracy in the estimation. For this reason, the calculated ΔG values are termed ΔG* throughout the text.

### 3.2. Chemistry, General Information

All hydrochlorides **1** were prepared according to the method reported in the literature [28]. All employed reagents, including aldehydes, carboxylic acids, and isocyanides, are commercially available or were synthesized according to the literature procedures. Solvents were purchased as “anhydrous” and used without further purification. ^1^H NMR, ^13^C NMR, and ^11^B NMR spectra were recorded using a Bruker AV 400 Ultrashield spectrometer. ^1^H NMR and ^13^C NMR chemical shifts were reported in parts per million (ppm) downfield from tetramethylsilane,^11^B NMR chemical shifts were determined relative to BF_3_·Et_2_O, and spectra were recorded using quartz NMR tubes. Coupling constants (*J*) were reported in Hertz (Hz). The residual solvent peaks were used as internal references: ^1^H NMR (CDCl_3_ 7.26 ppm, CD_3_CN 1.94 ppm) and ^13^C NMR (CDCl_3_ 77.0 ppm, CD_3_CN 1.32 and 118.26 ppm). The following abbreviations were used to explain the multiplicities: s = singlet, d = doublet, t = triplet, m = multiplet, br = broad, app = apparent. Reactions involving boron-containing compounds were followed by TLC using a curcumin solution, which was prepared as reported in the literature [29]. Chromatographic purifications were performed by Flash Chromatography (FC), using Merck Silica gel 60.

### 3.3. General Procedure (A) for the Synthesis of β-Amido Boronic Esters ***2***

In a flame-dried round-bottom flask, (*R*)-phenyl-β-amino boronic hydrochloride **1** (0.345 mmol, 1 eq) was suspended in dry dichloromethane (0.70 mL, 0.5M), then freshly distilled triethylamine (48 μL, 0.345 mmol, 1 eq) was added dropwise and the reaction stirred at room temperature for 5 min. Paraformaldehyde (0.345 mmol, 1 eq), carboxylic acid (0.380 mmol, 1.1 eq), and isocyanide (0.449 mmol, 1.3 eq) were added sequentially and the reaction was stirred at room temperature for 72 h (reaction changes from turbid pale-yellow to clear dark-yellow). The solvent was removed under reduced pressure and the crude product was purified by FC, to afford pure β-amido boronic esters **2**.

#### 3.3.1. (R)-N1-(2-(Cyclohexylamino)-2-oxoethyl)-N1-(1-phenyl-2-(4,4,5,5-tetramethyl-1,3,2-dioxaborolan-2-yl)ethyl)succinamide (**2a**)

Synthesized according to the General Procedure (A), using succinamic acid and cyclohexyl isocyanide. Purified by FC (dichloromethane/methanol 96:4) to afford compound **2a** as a pale-yellow foam (yield 35%). [α]^20^_D_ = +63.5 (c 1.0, CHCl_3_); ^1^H NMR (400 MHz, CDCl_3_, 55:45 rotameric mixture) δ 7.37–7.25 (m, 5H), 6.31 (br d, 1.45H), 6.16 (app t, 0.45H), 6.04 (br d, 0.45H), 5.62 (br d, 0.55H), 5.52 (br s, 0.55H), 5.45 (app t, 0.55H), 3.80–3.65 (m, 2H), 3.60–3.49 (m, 1H), 3.07–2.96 (m, 1H), 2.80–2.58 (m, 3H), 2.52–2.43 (m, 1H), 1.76–1.27 (m, 10H), 1.15 and 1.13 (s, 6H), 1.09 and 1.05 (s, 6H), 0.96–0.88 (m, 1H); ^13^C NMR (101 MHz, CDCl_3_, 55:45 rotameric mixture) δ 175.5 and 175.3 (Cq), 173.6 and 173.5 (Cq), 168.6 and 168.3 (Cq), 140.9 and 140.30 (Cq), 129.4, 129.3, 128.7, 128.6, 128.2, 84.3 and 84.1 (2 Cq), 57.7 and 53.8, 49.2 and 48.7, 47.9 and 47.0, 33.4 and 33.2 (2C), 33.0 and 32.8, 31.3 and 31.1, 29.6 and 29.1, 26.2 and 26.0, 25.8 and 25.7, 25.5 and 25.4 (2C), 25.3 and 25.2 (2C), 16.2 and 14.4 (CH_2_-B); ^11^B NMR (128 MHz, CDCl_3_) δ 33.6; HRMS (ESI) calcd for C_26_H_40_BN_3_NaO_5_^+^ [M + Na]^+^ 508.2959, found 508.2966.

#### 3.3.2. (R)-N1-(2-(Tert-butylamino)-2-oxoethyl)-N1-(1-phenyl-2-(4,4,5,5-tetramethyl-1,3,2-dioxaborolan-2-yl)ethyl)succinamide (**2b**)

Synthesized according to the General Procedure (A), using succinamic acid and tert-butyl isocyanide. Purified by FC (dichloromethane/methanol 96:4) to afford compound **2b** as a pale-yellow foam (yield 29%). [α]^20^_D_ = +40.3 (c 1.0, CHCl_3_); ^1^H NMR (400 MHz, CDCl_3_, 50:50 rotameric mixture) δ 7.39–7.28 (m, 5H), 6.22–6.11 (m, 1.5H), 5.77 (br s, 0.5H), 5.58 (br s, 0.5H), 5.50–5.42 (m, 1.5H), 3.74–3.61 (m, 2H), 3.16–3.02 (m, 1H), 2.80–2.60 (m, 2H), 2.55–2.48 (m, 1H), 1.69 (dd, *J*_2_ = 15.5 Hz, *J*_3_ = 8.9 Hz, 1H), 1.55–1.53 (m, 1H), 1.26 (s, 3H), 1.17 (s, 6H), 1.12 (s, 6H), 1.11 (s, 3H), 1.05 (s, 3H); ^13^C NMR (101 MHz, CDCl_3_, 50:50 rotameric mixture) δ 175.3 and 175.0 (Cq), 173.5 and 173.2 (Cq), 168.8 and 168.2 (Cq), 141.1 and 140.4 (Cq), 129.5 (2C), 128.7, 128.6, 128.2, 84.3 and 84.0 (2 Cq), 57.7 and 53.7, 51.9 and 51.4 (Cq), 48.2 and 47.9, 31.3 and 31.1, 29.9 and 29.4, 25.5, 25.4 (2C), 25.3 (2C), 25.22, 25.16, 16.2 and 14.5 (CH_2_-B); ^11^B NMR (128 MHz, CDCl_3_) δ 34.4; HRMS (ESI) calcd for C_24_H_38_BN_3_NaO_5_^+^ [M + Na]^+^ 482.2802, found 482.2799.

#### 3.3.3. (R)-N1-(2-(Benzylamino)-2-oxoethyl)-N1-(1-phenyl-2-(4,4,5,5-tetramethyl-1,3,2-dioxaborolan-2-yl)ethyl)succinamide (**2c**)

Synthesized according to the General Procedure (A), using succinamic acid and benzyl isocyanide. Purified by FC (dichloromethane/methanol 96:4) to afford compound **2c** as a pale-yellow foam (yield 31%). [α]^20^_D_ = +54.2 (c 1.0, CHCl_3_); ^1^H NMR (400 MHz, CDCl_3_, 65:35 rotameric mixture) δ 7.41–7.17 (m, 10H), 6.72 (br t, 0.65H), 6.13 (app t, 0.35H), 5.96–5.90 (m, 1.35H), 5.45 (app t, 0.65H), 5.40 (br s, 0.35H), 5.11 (br s, 0.65H), 4.37–4.23 (m, 2H), 3.91 (d, *J*_2_ = 16.0 Hz, 0.65H), 3.80 (s, 0.70H), 3.72 (d, *J*_2_ = 16.0 Hz, 0.65H), 3.09–2.99 (m, 1.30H), 2.75–2.61 (m, 2H), 2.57–2.52 (m, 0.70H), 1.69 (dd, *J*_2_ = 15.6 Hz, *J*_3_ = 8.4 Hz, 1H), 1.55 (dd, *J*_2_ = 15.6 Hz, *J*_3_ = 8.4 Hz, 1H), 1.18 (s, 3.90H), 1.14 (s, 2.10H), 1.13 (s, 3.90H), 1.07 (s, 2.10); ^13^C NMR (101 MHz, CDCl_3_, 65:35 rotameric mixture) δ 174.8 and 174.5 (Cq), 173.1 and 172.9 (Cq), 169.1 and 168.9 (Cq), 139.6 (Cq), 138.5 and 138.1 (Cq), 127.9, 128.8, 128.6, 128.5, 128.4, 128.1, 127.8, 127.6, 127.4, 127.0, 83.7 and 83.6 (2 Cq), 57.3 and 53.5, 47.6 and 46.5, 43.3 and 43.1, 30.8 and 30.6, 29.0 and 28.6, 24.8 (2C), 24.5 (2C), 15.5 and 15.2 (CH_2_-B); ^11^B NMR (128 MHz, CDCl_3_) δ 33.3; HRMS (ESI) calcd for C_27_H_36_BN_3_NaO_5_^+^ [M + Na]^+^ 516.2646, found 516.2650.

#### 3.3.4. (R)-N-(2-(Cyclohexylamino)-2-oxoethyl)-N-(1-phenyl-2-(4,4,5,5-tetramethyl-1,3,2-dioxaborolan-2-yl)ethyl)benzamide (**2d**)

Synthesized according to the General Procedure (A), using benzoic acid and cyclohexyl isocyanide. Purified by FC (hexane/ethyl acetate 6:4) to afford compound **2d** as a white foam (yield 55%). [α]^20^_D_ = +56.5 (c 1.0, CHCl_3_); ^1^H NMR (400 MHz, CDCl_3_, complex rotameric mixture: the section by section integration proves the overall number of protons) δ 7.65–7.11 (m, 10H), 6.36–5.30 (m, 1H), 4.61–3.15 (m, 4H), 1.98–1.88 (m, 2H), 1.69–1.38 (m, 10H), 1.15–1.09 (m, 12H); ^13^C NMR (101 MHz, CDCl_3_, complex rotameric mixture) δ 173.1 (1C), 170.1 and 168.7 (1C), 136.6 (1C), 134.1 (1C), 130.1–126.4 (10C), 84.0 (2C), 63.9 and 61.6 and 54.8 (1C), 63.2 and 59.0 and 58.5 (1C), 49.4–47.1 (1C), 33.2–33.0 (2C), 31.5 and 30.1 (1C), 25.9 (2C), 25.2–25.1 (4C), (CH_2_-B missing due to boron-quadrupole-induced relaxation); ^11^B NMR (128 MHz, CDCl_3_) δ 34.0; HRMS (ESI) calcd for C_29_H_39_BN_2_NaO_4_^+^ [M + Na]^+^ 513.2895, found 513.2891.

#### 3.3.5. (R)-N1-(2-(Benzylamino)-2-oxoethyl)-N1-(1-phenyl-2-(4,4,5,5-tetramethyl-1,3,2-dioxaborolan-2-yl)ethyl)glutaramide (**2ea**)

Synthesized according to the General Procedure (A), using 5-amino-5-oxopentanoic acid and benzyl isocyanide. Purified by FC (dichloromethane/methanol 96:4) to afford compound **2ea** as a yellow foam (yield 37%). [α]^20^_D_ = +40.1 (c 1.0, CHCl_3_); ^1^H NMR (400 MHz, CDCl_3_, 60:40 rotameric mixture) δ 7.36–7.15 (m, 10H), 0.46 (br t, 0.60H), 6.40 (br s, 1H), 6.09 (app t, 0.40H), 5.97 (br s, 0.40H), 5.73 (br s, 0.40H), 5.34 (app t, 0.60H), 4.77–4.68 (m, 0.60H), 4.38–4.22 (m, 2H), 3.92 (d, *J*_2_ = 16.0 Hz, 0.60H), 3.73 (s, 0.80H), 3.65 (d, *J*_2_ = 16.0 Hz, 0.60H), 2.89–2.70 (m, 1H), 2.37–2.23 (m, 3H), 2.08–1.95 (m, 2H), 1.68 (dd, *J*_2_ = 15.7 Hz, *J*_3_ = 8.3 Hz, 1H), 1.52 (dd, *J*_2_ = 15.7 Hz, *J*_3_ = 8.3 Hz, 1H), 1.16–1.09 (m, 12H); ^13^C NMR (101 MHz, CDCl_3_, 60:40 rotameric mixture) δ 176.5 and 176.4 (Cq), 174.2 and 174.1 (Cq), 170.1 and 169.8 (Cq), 141.3 and 140.6 (Cq), 139.0 and 138.7 (Cq), 129.5, 129.4, 129.3, 129.2, 128.7, 128.6, 128.3, 128.2, 128.1, 127.8, 84.4 and 84.3 (2Cq), 57.7 and 54.0, 48.3 and 47.3, 43.9, 35.3 and 35.2, 33.5 and 32.7, 25.34, 25.30, 25.22 (2C), 21.6 and 21.5, 16.4 and 14.5 (CH_2_-B); ^11^B NMR (128 MHz, CDCl_3_) δ 33.2; HRMS (ESI) calcd for C_28_H_38_BN_3_NaO_5_^+^ [M + Na]^+^ 530.2802, found 530.2807.

#### 3.3.6. (R)-N1-(2-(Tert-butylamino)-2-oxoethyl)-N1-(1-phenyl-2-(4,4,5,5-tetramethyl-1,3,2-dioxaborolan-2-yl)ethyl)glutaramide (**2f**)

Synthesized according to the General Procedure (A), using 5-amino-5-oxopentanoic acid and tert-butyl isocyanide. Purified by FC (dichloromethane/methanol 96:4) to afford compound **2f** as a pale-yellow foam (yield 29%). [α]^20^_D_ = +76.7 (c 1.0, CHCl_3_); ^1^H NMR (400 MHz, CDCl_3_, 55:45 rotameric mixture) δ 7.40–7.29 (m, 5H), 6.35 (br s, 0.45H), 6.23 (app t, 0.55H), 6.05 (br s, 0.45H), 5.83 (br s, 0.55H), 5.54–5.49 (m, 1H), 5.33 (app t, 0.45H), 5.19 (br s, 0.55H), 3.77 (d, *J*_2_ = 15.4 Hz, 0.55H), 3.71 (s, 0.90H), 3.56 (d, *J*_2_ = 15.4 Hz, 0.55H), 2.90–2.73 (m, 1H), 2.40–2.24 (m, 3H), 2.11–2.07 (m, 1H), 2.06–2.00 (m, 1H), 1.68 (dd, *J*_2_ = 15.8 Hz, *J*_3_ = 9.4 Hz, 1H), 1.49–1.44 (m, 1H), 1.21 (s, 4.70H), 1.18 (s, 3H), 1.15 (s, 3H), 1.12 (s, 3H), 1.07 (s, 7.30); ^13^C NMR (101 MHz, CDCl_3_, 55:45 rotameric mixture) δ 176.1 and 175.7 (Cq), 174.0 and 173.8 (Cq), 169.1 and 168.5 (Cq), 141.4 and 140.8 (Cq), 129.7, 129.4, 128.7, 128.6, 127.9, 84.3 and 84.1 (2Cq), 57.9 and 53.4, 51.8 and 51.5 (Cq), 48.2 and 48.0, 35.7 and 35.5, 33.4 and 32.6, 29.2 (2C), 28.9 (2C), 25.4, 25.32, 25.26, 21.9 and 21.6, 16.8 and 14.5 (CH_2_-B); ^11^B NMR (128 MHz, CDCl_3_) δ 33.0; HRMS (ESI) calcd for C_25_H_40_BN_3_NaO_5_^+^ [M + Na]^+^ 496.2959, found 496.2964.

#### 3.3.7. (R)-N1-(2-(Cyclohexylamino)-2-oxoethyl)-N1-(1-phenyl-2-(4,4,5,5-tetramethyl-1,3,2-dioxaborolan-2-yl)ethyl)glutaramide (**2g**)

Synthesized according to the General Procedure (A), using 5-amino-5-oxopentanoic acid and cyclohexyl isocyanide. Purified by FC (Dichloromethane/Methanol 96:4) to afford compound **2g** as a pale-yellow foam (yield 27%). [α]^20^_D_ = +51.0 (c 1.0, CHCl_3_); ^1^H NMR (400 MHz, CDCl_3_, 65:35 rotameric mixture) δ 7.37–7.26 (m, 5H), 6.20–6.14 (m, 0.70H), 6.03–5.91 (m, 1H), 5.44 (app t, 0.65H), 5.38–5.32 (m, 0.65H), 3.79 (d, *J*_2_ = 15.8 Hz, 0.65H), 3.75 (s, 0.70H), 3.68 (d, *J*_2_ = 15.8 Hz, 0.65H), 3.61–3.49 (m, 1H), 3.06–3.03 (m, 1H), 2.80–2.44 (m, 3H), 1.79–1.72 (m, 2H), 1.71–1.24 (m, 12H), 1.16–1.14 (m, 6H), 1.10–1.07 (m, 6H), (1 exchangeable NH proton missing); ^13^C NMR (101 MHz, CDCl_3_, 65:35 rotameric mixture) δ 175.3 and 175.0 (Cq), 173.4 and 173.2 (Cq), 168.3 and 167.9 (Cq), 140.7 and 140.1 (Cq), 128.9, 128.7, 128.01 and 127.96, 127.8, 127.2, 83.7 and 83.6 (2Cq), 57.2 and 52.8, 48.2 and 48.0, 47.4 and 46.8, 35.1 and 34.8, 32.8 and 32.72, 32.68 and 32.5, 32.1 and 32.0, 25.5 and 25.4, 24.9 and 24.83, 24.75 (2C), 24.68 (2C), 24.6, 21.1 and 20.9, 15.9 and 13.7 (CH_2_-B); ^11^B NMR (128 MHz, CDCl_3_) δ 34.3; HRMS (ESI) calcd for C_27_H_42_BN_3_NaO_5_^+^ [M + Na]^+^ 522.3115, found 522.3111.

#### 3.3.8. Tert-Butyl ((S)-1-((2-(Benzylamino)-2-oxoethyl)((R)-1-phenyl-2-(4,4,5,5-tetramethyl-1,3,2-dioxaborolan-2-yl)ethyl)amino)-3-(4-hydroxyphenyl)-1-oxopropan-2-yl)carbamate (**2h**)

Synthesized according to the General Procedure (A), using (*tert*-butoxycarbonyl)-L-tyrosine, and benzyl isocyanide. Purified by FC (hexane/ethyl acetate 6:4) to afford compound **2h** as a white foam (yield 37%). [α]^20^_D_ = +42.9 (c 1.0, CHCl_3_); ^1^H NMR (400 MHz, CDCl_3_, 50:50 rotameric mixture) δ 7.31–7.00 (m, 12H), 6.87 (br s, 1H), 6.75–6.67 (m, 2H), 6.03–5.99 (m, 0.50H), 5.49 (br. s, 0.50H), 5.14–5.13 (m, 1H), 5.02–4.97 (m, 0.50H), 4.38–4.28 (m, 1.50H), 4.12–4.10 (m, 1H), 3.76–3.74 (m, 2H), 3.03–2.96 (m, 1H), 2.85–2.75 (m, 1H), 1.74 (dd, *J_2_* = 15.3 Hz, *J_3_* = 9.9 Hz, 1H), 1.55 (dd, *J_2_* = 15.3 Hz, *J_3_* = 9.9 Hz, 1H), 1.39–1.37 (m, 9H), 1.16 (s, 3H), 1.13 (s, 3H), 1.09 (s, 3H), 1.04 (s, 3H), (1 exchangeable NH proton missing); ^13^C NMR (101 MHz, CDCl_3_, 50:50 rotameric mixture) δ 173.2 and 173.1 (Cq), 169.2 (Cq) 168.7 (Cq), 155.9 and 155.6 (Cq), 139.6 and 139.3 (Cq), 138.3 (Cq) 137.6 (Cq), 130.5, 130.3, 128.8–126.9 (11C), 116.8 and 116.7, 83.7 and 83.5 (2C), 80.3 and 80.2 (Cq), 57.9 and 52.4, 54.5 and 53.7, 47.1 and 46.7, 43.5 and 43.1, 37.8 and 37.6, 28.3 and 28.2 (3C), 24.7 (2C), 24.5 (2C), (CH_2_-B missing due to boron-quadrupole-induced relaxation); ^11^B NMR (128 MHz, CDCl_3_) δ 32.9; HRMS (ESI) calcd for C_37_H_49_BN_3_O_7_^+^ [M + H]^+^ 658.3658, found 658.3670.

#### 3.3.9. (S)-N1-(2-(Benzylamino)-2-oxoethyl)-N1-(1-phenyl-2-(4,4,5,5-tetramethyl-1,3,2-dioxaborolan-2-yl)ethyl)glutaramide (**2eb**)

Synthesized according to the General Procedure (A), with the exception of using (*S*)-phenyl-β-amino boronic hydrochloride **1**, as well as paraformaldehyde, 5-amino-5-oxopentanoic acid, and benzyl isocyanide. Purified by FC (dichloromethane/methanol 96:4) to afford compound **2eb** as a yellow foam (yield 36%). [α]^20^_D_ = −39.8 (c 1.0, CHCl_3_); ^1^H, ^13^C, and ^11^B NMR data are identical to those of compound **2ea**, previously reported. HRMS (ESI) calcd for C_28_H_38_BN_3_NaO_5_^+^ [M + Na]^+^ 530.2802, found 530.2799.

### 3.4. General Procedure (B) for the Synthesis of β-Amido Boronic Acids ***3***

The reaction was performed following a modified literature procedure [30]. In a round-bottom flask, the desired β-amido boronic ester **2** (0.20 mmol, 1 eq) and methylboronic acid (2.00 mmol, 10 eq) were dissolved in an acetone/0.2 N HCl_aq_ (1:1 *v*/*v*) solution (5 mL, 0.04 M) and stirred at room temperature for 4 h (the reaction changes from yellow to pale-yellow). The solvent was evaporated under reduced pressure using a 50 °C bath, then the reaction was diluted with 0.2 NHCl_aq_ (1.0 mL) and evaporated again under reduced pressure. The crude was then dissolved in MeCN/H_2_O 1:1 (4 mL) and freeze-dried to afford pure β-amido boronic acids **3**.

#### 3.4.1. (R)-(2-(4-Amino-N-(2-(cyclohexylamino)-2-oxoethyl)-4-oxobutanamido)-2-phenylethyl)boronic Acid (**3a**)

Synthesized according to the General Procedure (B) starting from compound **2a**. Product **3a** was obtained as a yellow powder (yield 99%). [α]^20^_D_ = +33.4 (c 1.0, MeCN/H_2_O 1:1); ^1^H NMR (400 MHz, CD_3_CN + 1 drop of H_2_O, 60:40 rotameric mixture) δ 7.43–7.29 (m, 5H), 7.09–6.80 (m, 3H), 5.32 (dd, *J_3_* = 9.7 Hz, *J_3_* = 4.8 Hz 0.60H), 5.46 (dd, *J_3_* = 9.7 Hz, *J_3_* = 4.8 Hz 0.40H), 3.69–3.40 (m, 3H), 2.67–2.58 (m, 2H), 1.84–1.55 (m, 6H), 1.44–1.10 (m, 8H), (two exchangeable B(OH)_2_ protons missing); ^13^C NMR (101 MHz, CD_3_CN + 1 drop of H_2_O, 60:40 rotameric mixture) δ 176.4 and 175.1 (Cq), 174.3 and 173.8 (Cq), 170.2 and 169.3 (Cq), 141.7 and 141.6 (Cq), 129.3, 129.2, 128.6, 128.4 and 128.3, 128.1, 57.7 and 54.9, 49.3 and 49.1, 47.4 and 46.7, 33.0, 32.8, 32.7, 29.3 and 28.7, 25.9 and 25.4 (2C), 25.3, 18.5 and 18.0 (CH_2_-B); ^11^B NMR (128 MHz, CD_3_CN + 1 drop of H_2_O) δ 32.3 (-B(OH)_2_), 19.8 (-B(OH)_2_·H_2_O); HRMS (ESI) calcd for C_22_H_34_BNaN_3_O_5_^+^ [MB(OMe)_2_ + Na]^+^ 454.2489, found 454.2495.

#### 3.4.2. (R)-(2-(4-Amino-N-(2-(tert-butylamino)-2-oxoethyl)-4-oxobutanamido)-2-phenylethyl)boronic Acid (**3b**)

Synthesized according to the General Procedure (B) starting from compound **2b**. Product **3b** was obtained as a yellow powder (yield >99%). [α]^20^_D_ = +27.0 (c 1.0, MeCN/H_2_O 1:1); ^1^H NMR (400 MHz, CD_3_CN + 1 drop of H_2_O, 70:30 rotameric mixture) δ 7.42–7.28 (m, 5H), 7.02–6.59 (m, 2H), 5.57–5.54 (m, 0.70H), 5.46 (dd, *J*_3_ = 9.3 Hz, *J*_3_ = 5.6 Hz, 0.30H), 3.90–3.68 (m, 2H), 3.17–3.08 (m, 1H), 2.76–2.64 (m, 3H), 1.33–1.31 (m, 2.70H), 1.30–1.25 (m, 2H), 1.24–1.22 (m, 6.30H), (two exchangeable B(OH)_2_ and one NH protons missing); ^13^C NMR (101 MHz, CD_3_CN + 1 drop of H_2_O, 70:30 rotameric mixture) δ 178.1 and 177.8 (Cq), 176.5 and 175.1 (cq), 169.5 and 167.9 (Cq), 141.2 and 140.8 (Cq), 129.4 (2C), 128.7 (2C), 128.2, 58.8 and 58.2, 55.0 (Cq), 49.6, 47.1, 43.9, 28.5, 28.4 (2C), 19.5 and 18.5 (CH_2_-B); ^11^B NMR (128 MHz, CD_3_CN + 1 drop of H_2_O) δ 32.3 (-B(OH)_2_), 19.9 (-B(OH)_2_·H_2_O); HRMS (ESI) calcd for C_20_H_32_BNaN_3_O_5_^+^ [MB(OMe)_2_ + Na]^+^ 428.2333, found 428.2329.

#### 3.4.3. (R)-(2-(4-Amino-N-(2-(benzylamino)-2-oxoethyl)-4-oxobutanamido)-2-phenylethyl)boronic Acid (**3c**)

Synthesized according to the General Procedure (B) starting from compound **2c**. Product **3c** was obtained as a dark-yellow powder (yield >99%). [α]^20^_D_ = +26.8 (c 1.0, MeCN/H_2_O 1:1); ^1^H NMR (400 MHz, CD_3_CN + 1 drop of H_2_O, 55:45 rotameric mixture) δ 7.67–7.07 (m, 12H), 5.67–5.57 (m, 0.55H), 5.48 (app t, 0.45H), 4.44–4.24 (m, 2H), 4.03–3.74 (m, 2H), 3.17–2.93 (m, 1H), 2.84–2.53 (m, 3H), 1.53–1.21 (m, 2H), (two exchangeable B(OH)_2_ and one NH protons missing); ^13^C NMR (101 MHz, CD_3_CN + 1 drop of H_2_O, 55:45 rotameric mixture) δ 178.1 and 177.8 (Cq), 176.3 and 174.1 (Cq), 171.1 and 169.1 (Cq), 141.2 and 140.7 (Cq), 139.2 and 138.9 (Cq), 129.3, 129.2, 129.1, 128.8, 128.7, 128.5, 128.2, 128.0, 127.9, 127.7, 58.6 and 58.1, 49.1 and 46.7, 43.7 and 43.4, 30.7, 29.6 and 28.9, 19.5 and 18.7 (CH_2_-B); ^11^B NMR (128 MHz, CD_3_CN + 1 drop of H_2_O) δ 32.3 (-B(OH)_2_), 19.9 (-B(OH)_2_·H_2_O); HRMS (ESI) calcd for C_23_H_30_BNaN_3_O_5_^+^ [MB(OMe)_2_ + Na]^+^ 426.2176, found 426.2173.

#### 3.4.4. (R)-(2-(N-(2-(Cyclohexylamino)-2-oxoethyl)benzamido)-2-phenylethyl)boronic Acid (**3d**)

Synthesized according to the General Procedure (B) starting from compound **2d**. Product **3d** was obtained as a white powder (yield 98%). [α]^20^_D_ = +57.4 (c 1.0, CHCl_3_); ^1^H NMR (400 MHz, CDCl_3_, complex rotameric mixture: the section by section integration proves the overall number of protons) δ 7.58–7.13 (m, 10H), 6.14–6.05 (m, 1H), 5.31–5.18 (m, 1H), 4.13–3.68 (m, 2H), 3.61–3.32 (m, 1H), 1.78–1.10 (m, 12H), (two exchangeable B(OH)_2_ protons missing); ^13^C NMR (101 MHz, CDCl_3_, complex rotameric mixture) δ 173.4 and 172.7 (Cq), 169.6 and 168.4 (Cq), 141.0 (Cq), 136.1 and 135.8 (Cq), 129.7, 128.7 (2C), 128.6 (2C), 127.7 (2C), 126.9, 126.7 (2C), 58.9 and 58.7, 48.9 and 48.8, 47.3, 32.63, 32.58, 25.4, 24.7, 24.6, 17.8 and 17.3 (CH_2_-B); ^11^B NMR (128 MHz, CDCl_3_) δ 32.5; HRMS (ESI) calcd for C_25_H_33_BNaN_2_O_4_^+^ [MB(OMe)_2_ + Na]^+^ 459.2431, found 459.2434.

#### 3.4.5. (R)-(2-(5-Amino-N-(2-(benzylamino)-2-oxoethyl)-5-oxopentanamido)-2-phenylethyl)boronic Acid (**3ea**)

Synthesized according to the General Procedure (B) starting from compound **2ea**. Product **3ea** was obtained as a dark-yellow powder (yield >99%). [α]^20^_D_ = +39.2 (c 1.0, MeCN/H_2_O 1:1); ^1^H NMR (400 MHz, CD_3_CN + 1 drop of H_2_O, 55:45 rotameric mixture) δ 8.19–6.79 (m, 12H), 5.84 (dd, *J*_3_ = 9.4 Hz, *J*_3_ = 7.0 Hz, 0.45H), 5.42 (dd, *J*_3_ = 9.4 Hz, *J*_3_ = 7.0 Hz, 0.55H), 4.40–4.36 (m, 1H), 4.31–4.26 (m, 1H), 3.85 (d, *J*_2_ = 18.1 Hz, 0.45H), 3.74 (d, *J*_2_ = 18.1 Hz, 0.45H), 3.72 (d, *J*_2_ = 16.3 Hz, 0.55H), 3.45 (d, *J*_2_ = 16.3 Hz, 0.55H), 2.77–2.66 (m, 1H), 2.38–2.21 (m, 2H), 1.96–1.83 (m, 2H), 1.49–1.39 (m, 1H), 1.34–1.29 (m, 2H), (two exchangeable B(OH)_2_ and one NH protons missing); ^13^C NMR (101 MHz, CD_3_CN + 1 drop of H_2_O, 55:45 rotameric mixture) δ 178.7 and 178.2 (Cq), 176.7 and 174.4 (Cq), 171.3 and 169.3 (Cq), 141.5 and 141.1 (Cq), 139.2 and 139.0 (Cq), 129.4 (2C), 129.2, 129.1, 128.7, 128.6 and 128.5, 128.3, 128.1, 128.0 and 127.9, 127.7, 58.3 and 57.9, 49.1 and 46.8, 43.7 and 43.4, 35.3 and 35.1, 33.4 and 32.6, 21.7 and 21.5, 19.5 and 18.5 (CH_2_-B); ^11^B NMR (128 MHz, CD_3_CN + 1 drop of H_2_O) δ 32.4 (-B(OH)_2_), 19.9 (-B(OH)_2_·H_2_O); HRMS (ESI) calcd for C_24_H_32_BNaN_3_O_5_^+^ [MB(OMe)_2_ + Na]^+^ 476.2333, found 476.2337.

#### 3.4.6. (R)-(2-(5-Amino-N-(2-(tert-butylamino)-2-oxoethyl)-5-oxopentanamido)-2-phenylethyl)boronic Acid (**3f**)

Synthesized according to the General Procedure (B) starting from compound **2f**. Product **3f** was obtained as a pale-yellow powder (yield 99%). [α]^20^_D_ = +51.8 (c 1.0, MeCN/H_2_O 1:1); ^1^H NMR (400 MHz, CD_3_CN + 1 drop of H_2_O, 65:35 rotameric mixture) δ 7.47–7.32 (m, 5H), 7.26–6.56 (m, 2H), 5.61 (app t, 0.65H), 5.41 (dd, *J*_3_ = 9.8 Hz, *J*_3_ = 5.6 Hz, 0.35H), 3.76 (br s, 1.30H), 3.66 (d, *J*_2_ = 15.8 Hz, 0.35H), 3.27 (d, *J*_2_ = 15.8 Hz, 0.35H), 2.80–2.71 (m, 1H), 2.51–2.31 (m, 4H), 1.96–1.89 (m, 1H), 1.46–1.40 (m, 1H), 1.36–1.29 (m, 3.15H), 1.25 (br s, 6.85H), (two exchangeable B(OH)_2_ and one NH protons missing); ^13^C NMR (101 MHz, CD_3_CN + 1 drop of H_2_O, 65:35 rotameric mixture) δ 178.6 and 178.3 (Cq), 176.6 and 174.3 (Cq), 170.5 and 168.3 (Cq), 141.6 and 141.3 (Cq), 129.4 (2C), 128.6, 128.5, 128.1, 58.1 and 57.8, 52.0 and 51.8 (Cq), 49.3 and 47.1, 34.8, 33.2 and 32.5, 28.5 and 28.4 (3C), 21.7 and 21.5, 19.5 and 18.2 (CH_2_-B); ^11^B NMR (128 MHz, CD_3_CN + 1 drop of H_2_O) δ 32.3 (-B(OH)_2_), 20.0 (-B(OH)_2_·H_2_O); HRMS (ESI) calcd for C_21_H_34_BNaN_3_O_5_^+^ [MB(OMe)_2_ + Na]^+^ 442.2489, found 442.2492.

#### 3.4.7. (R)-(2-(5-Amino-N-(2-(cyclohexylamino)-2-oxoethyl)-5-oxopentanamido)-2-phenylethyl)boronic acid (**3g**)

Synthesized according to the General Procedure (B) starting from compound **2g**. Product **3g** was obtained as a fluoro-yellow powder (yield >99%). [α]^20^_D_ = +27.3 (c 1.0, MeCN/H_2_O 1:1); ^1^H NMR (400 MHz, CD_3_CN + 1 drop of H_2_O, 60:40 rotameric mixture) δ 7.44–7.24 (m, 5H), 7.18–6.75 (m, 1H), 5. 53 (app, t, 0.60H), 5.42 (dd, *J*_3_ = 9.6 Hz, *J*_3_ = 6.4 Hz, 0.40H), 3.88–3.78 (m, 1.20H), 3.68 (d, *J*_2_ = 15.7 Hz, 0.40H), 3.61–3.52 (m, 1H), 3.36 (d, *J*_2_ = 15.7 Hz, 0.40H), 2.76 (t, *J*_3_ = 6.7 Hz, 0.60H), 2.52–2.35 (m, 3H), 1.96–1.91 (m, 1H), 1.85–1.60 (m, 5.40H), 1.45–1.40 (m, 1H), 1.37–1.11 (7H), (two exchangeable B(OH)_2_ and two NH protons missing); ^13^C NMR (101 MHz, CD_3_CN + 1 drop of H_2_O, 60:40 rotameric mixture) δ 179.1 and 178.7 (Cq), 176.8 and 174.5 (Cq), 170.3 and 168.1 (Cq), 141.5 and 141.1 (Cq), 129.4 and 129.2 (2C), 128.6 and 128.4 (2C), 128.1, 58.7 and 57.9, 49.4 and 49.3, 46.7 and 43.3, 34.9, 33.4 and 32.5, 32.8 (2C), 25.9, 25.3, 25.2, 21.7 and 21.5, 19.8 and 18.4; ^11^B NMR (128 MHz, CD_3_CN + 1 drop of H_2_O) δ 32.3 (-B(OH)_2_), 19.4 (-B(OH)_2_·H_2_O); HRMS (ESI) calcd for C_23_H_36_BNaN_3_O_5_^+^ [MB(OMe)_2_ + Na]^+^ 468.2646, found 468.2649.

#### 3.4.8. (S)-1-((2-(Benzylamino)-2-oxoethyl)((R)-2-borono-1-phenylethyl)amino)-3-(4-hydroxyphenyl)-1-oxopropan-2-aminium Chloride (**3h**)

In a round-bottom flask, compound **2h** (0.20 mmol, 1 eq) was dissolved in dry DCM (0.7 mL, 0.30 M), then TFA (0.3 mL, 1.76 mmol, 9 eq) was added dropwise and the reaction stirred at room temperature for 10 min. The solvent was removed under reduced pressure to afford the N-Boc-deprotected intermediate as a white powder (yield >99%). [α]^20^_D_ = +25.1 (c 1.0, CHCl_3_); HRMS (ESI) calcd for C_32_H_41_BN_3_O_5_^+^ [M]^+^ 558.3134, found 558.3130. The obtained N-Boc-deprotected intermediate was treated according to the General Procedure (B), to obtain pure compound **3h** as a dark-yellow powder (yield 99%). [α]^20^_D_ = +44.7 (c 1.0, MeCN/H_2_O 1:1); ^1^H NMR (400 MHz, CD_3_CN + 1 drop of H_2_O, 60:40 rotameric mixture) δ 8.00 (br s, 3H), 7.51 (br dd, amide NH, 0.40H), 7.42 (br dd, amide NH, 0.60H), 7.35–7.22 (m, 8H), 7.12–7.10 (m, 2H), 7.00 and 6.96 (d, *J*_3_ = 7.4 Hz, 2H), 6.83 and 6.79 (d, *J*_3_ = 8.5 Hz, 2H), 5.99 (app t, 0.60H), 5.40 (app t, 0.40H), 5.00 (br s, 0.40H), 4.33–4.27 (m, 1.60H), 4.00 (br d, 1H), 3.71 (d, *J*_2_ = 17.8 Hz, 0.60H), 3.58 (hidden by the H_2_O signal, detected by ^1^H/^13^C HSQC, 0.80H), 3.44 (d, *J*_2_ = 17.8 Hz, 0.60H), 3.13–3.09 (m, 2H), 1.55 (d, *J*_3_ = 8.0 Hz, 0.80H), 1.47 (dd, *J*_2_ = 15.4 Hz, *J*_3_ = 9.7 Hz, 0.60H), 1.17 (dd, *J*_2_ = 15.4 Hz, *J*_3_ = 9.7 Hz, 0.60H), (two exchangeable B(OH)_2_ and one NH protons missing); ^13^C NMR (101 MHz, CD_3_CN + 1 drop of H_2_O, 60:40 rotameric mixture) δ 170.4 and 170.08 (Cq), 170.05 and 169.3 (Cq), 157.5 and 157.4 (Cq), 140.6 and 139.3 (Cq), 139.2 (Cq), 138.5 (Cq), 131.8, 131.6, 129.5, 129.4, 129.1 (2C), 128.9, 128.6 and 128.5, 128.2, 128.1, 127.9, 127.8, 125.5 and 125.3, 116.6 and 116.5, 58.7 and 55.5, 53.8 and 52.4, 47.3 and 45.9, 43.6 and 43.5, 36.9 and 36.7, 20.2 and 18.1 (CH_2_-B); ^11^B NMR (128 MHz, CD_3_CN + 1 drop of H_2_O) δ 32.4 (-B(OH)_2_), 19.9 (-B(OH)_2_·H_2_O); HRMS (ESI) calcd for C_28_H_35_BN_3_O_5_^+^ [MB(OMe)_2_]^+^ 504.2604, found 504.2609.

#### 3.4.9. (S)-(2-(5-Amino-N-(2-(benzylamino)-2-oxoethyl)-5-oxopentanamido)-2-phenylethyl)boronic Acid (**3eb**)

Synthesized according to the General Procedure (B) starting from compound **2eb**. Product **3eb** was obtained as a dark-yellow powder (yield >99%). [α]^20^_D_ = −40.1 (c 1.0, MeCN/H_2_O 1:1); ^1^H, ^13^C, and ^11^B NMR data are identical to those of compound **3ea**, previously reported. HRMS (ESI) calcd for C_24_H_32_BNaN_3_O_5_^+^ [MB(OMe)_2_ + Na]^+^ 476.2333, found 476.2330.

### 3.5. MST Experiments

The ligand capacity to bind Mpro^CoV-2^ was measured by Monolith NT.115 instrument (NanoTemper Technologies GmbH, München, Germany). Briefly, histidine-tagged Mpro^CoV-2^ was labeled by using a non-covalent His-tag dye for 30 min at room temperature, using the His-Tag Labeling Kit RED-tris-NTA 2nd Generation (MO-L018), purchased from NanoTemper Technologies (GmbH, München, Germany). A fixed concentration of the labeled Mpro^CoV-2^ enzyme (50 nM) was mixed with different dilutions of the synthesized β-amido boronic compounds, and “binding check” experiments were conducted, using the “expert mode” of the Monolith software MO.Control v1.6 (München, Germany). At least two concentration points for each compound were performed for the F_norm_ evaluation. The enzyme and the ligands were incubated for 60–90 min at room temperature. The MST measurements were accomplished using standard capillaries, using a medium MST power (40%), in order to create the temperature gradient, and an excitation power of 60% at the temperature of 25 °C. The ligand capability to bind the enzyme (Table 2) was calculated from compound concentration-dependent changes in normalized fluorescence (F_norm_). In all the experiments, both interacting species were dissolved in PBS-T buffer (phosphate-buffered saline + 0.05% Tween ™ 20) of NanoTemper Technologies (GmbH, München, Germany) and 2.5% DMSO. The auto-fluorescence of each ligand was assessed before proceeding to the evaluation of the F_norm_. In all the “binding check” assays performed, only the time of 1.5 s was considered for the evaluation of the F_norm_. Consequently, the F_norm_ difference between the one observed for the ligand and the Mpro^CoV-2^ protein (namely, the Response Amplitude, RA), necessary to consider the ligand binding, must be greater than 1.

The capability of **3ea** compound to specifically bind the Mpro^CoV-2^ protein was assessed through the “binding affinity” experiment on the 6His Peptide Control provided by NanoTemper Technologies (GmbH, München, Germany), applying the standard suggested protocol. Initially, the protein was labeled using the same protocol previously adopted, then a fixed concentration of the labeled Mpro^CoV-2^ enzyme (50 nM) was mixed with sixteen 1:1 serial dilutions of the **3ea** compound (ranging from 100 to 3 nM). The protein and the molecule were incubated for at least 15 min at room temperature. The MST measurements were accomplished using a medium MST power, and an excitation power of 40% at the temperature of 25 °C, using standard capillaries. The full analysis report was generated using the Monolith software MO.Affinity Analysis v2.3 (München, Germany) (Figure S3, Supporting Information).

### 3.6. MST Experiments to Ascertain the Reversible or Irreversible Inhibition of M^pro^

We conducted these experiments by pre-incubating the Mpro^Cov-2^/**3ea** complex with a protein concentration of 400 nM, in order to obtain a sufficient fluorescence value for the subsequent dilution of the ligand/enzyme complex. At that target concentration, we verified the concentration of **3ea,** in which no binding was observed, obtaining a ligand concentration of 2 µM (Figure S4A, Supplementary Materials). Then, we measured the F_norm_ of the other solutions obtained progressively by diluting a Mpro^Cov-2^/**3ea** complex solution to 6.25, 3.125, and 1.56 µM.

### 3.7. LC-MS/MS Experiment to Verify the Ligand Binding of M^pro^

The **3ea** sample (75 µL, 20 µM) was incubated with purified M^pro^ (75 µL, 400 nM) for 16 h at 4 °C, in the dark. The sample was concentrated and filtered through Amicon Centrifugal Filter Devices with a 3 kDa cut-off (Merck Millipore, Milan, Italy) for 30 min at 3030× *g*. Then, the eluent solution (5 µL) was analyzed for protein-free ligands using LC-MS/MS. The supernatant solution (ligand bound to M^pro^ + free ligand, 20 µL) was deproteinized by ACN (20 µL) and centrifuged (10 min at 3030× *g*) to displace the binding between the protease and β-amido boronic acid. Then, an aliquot (5 µL) was injected in LC-MS/MS. UHPLC–MS/MS analyses were performed on a 1290 Infinity ultra-high-performance liquid chromatography system (Agilent Technologies, Palo Alto, CA, USA) coupled to a Q Trap 5500 linear ion trap triple quadrupole mass spectrometer (Sciex, Darmstadt, Germany) and equipped with an electrospray ionization (ESI) source. Chromatographic separation was achieved on a reversed-phase Zorbax SB-C18 column 3.5 μm, 2.1 × 150 mm (Agilent Technologies, Palo Alto, CA, USA) equipped with pre-column using as mobile phases (A) water + 0.2 mM ammonium acetate and (B) acetonitrile. The flow rate was 0.5 mL/min and the column temperature was set to 40 °C. The elution gradient (%B) was set as follows: 0–1 min (1%), 1–4 min (1–95%), 4–8 min (95%), 8–8.1 (95–1%), held until 2 min. Analyses were performed by multiple reaction monitoring (MRM) in negative mode according to the transitions corresponding to **3ea**: *m*/*z* 424.6 > 92.3 (target ion, DP −37, CE −47 eV); *m*/*z* 424.6 > 208.0 (DP −37, CE −20 eV); *m*/*z* 424.6 > 268.0 (target ion, DP −37, CE −20 eV); *m*/*z* 424.6 > 326.0 (DP −37, CE −16 eV); *m*/*z* 210.8 > 152.8 (DP −37, CE −18 eV); *m*/*z* 210.8 > 95 (DP −37, CE −18 eV) (Figure S6, Supplementary Materials).

### 3.8. Enzymatic Assays

The inhibitory activity of the compounds was evaluated by a Förster resonance energy transfer (FRET)-based enzymatic cleavage assay on a TECAN Infinite F2000 PRO plate reader (Agilent Technologies, Santa Clara, CA, USA) using white flat-bottom 96-well microtiter plates (Greiner bio-one, Kremsmünster, Austria) [31]. Nirmatrelvir was purchased from AOBIUS (Gloucester, MA, USA) and used as positive control. Recombinant Mpro^CoV-2^ was expressed and purified as previously described [32], whereas the peptidic substrate Dabcyl-KTSAVLQ↓SGFRKME-Edans (TFA salt) was obtained by commercial source (Genescript, NJ, USA). The arrow indicates the cleavage position. The proteolytic activity of the Mpro^CoV-2^ was measured by monitoring the increasing fluorescence of SGFRKME-Edans upon hydrolytic shedding of the quencher Dabcyl-KTSAVLQ, at 25 °C with a 335 nm excitation filter and a 493 nm emission filter. Each well contained 200 µL composed of 185 µL reaction buffer (20 mM Tris pH 7.5, 0.1 mM EDTA, 1 mM DTT, and 200 mM NaCl), 5 µL Mpro^CoV-2^ in enzyme buffer at a final concentration of 50 nM together with 5 µL of the fluorogenic substrate (final concentration 25 μM) and 10 µL of the compounds present at a final concentration of 20 μM (screening assay). DMSO was used as a negative control. Inhibitors and substrate were dissolved and diluted in DMSO, leading to a final DMSO concentration of 7.5% (*v*/*v*). The compounds and enzyme were incubated for 10 min at 25 °C prior to substrate addition. Product release from substrate hydrolysis was monitored in 30 s increments over a period of 10 min. The related *K*_M_ value was determined in a separate experiment (33 µM). IC_50_ value was determined as previously described by us [33].

## 4. Conclusions

In this study, to investigate whether SARS-CoV-2 M^pro^ Thr25 could be targeted by boron-containing compounds, we designed and synthesized eight compounds displaying affinity to the target in the low micromolar range, as suggested by MST experiments. Enzymatic assays suggested that the most promising of the eight compounds slightly inhibited the catalytic activity of the target, permitting us to suppose that an allosteric site of the enzyme is covalently targeted by the compounds. However, our results provide experimental evidence that BCCs represent new and promising Mpro^CoV-2^ inhibitors. We are confident that this research can pave the way toward the design of new boron-containing compounds with antiviral activity against SARS-CoV-2, potentially useful for the treatment of diseases caused by coronaviruses.

## Figures and Tables

**Table 1:** Chemical structure and predicted binding free energy values (ΔG*) of the most promising β-amido boronic acids.

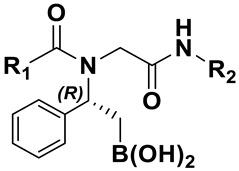			
Compound	R_1_	R_2_	ΔG* (Kcal/mol)
**3a**	2-aminocarbonyl-ethyl	-Cy	−64.9 ± 6.2
**3b**	2-aminocarbonylethyl	-*t*Bu	−42.7 ± 5.7
**3c**	2-aminocarbonylethyl	-Bn	−70.5 ± 6.4
**3d**	phenyl	-Cy	−48.8 ± 8.0
**3ea**	3-aminocarbonylpropyl	-Bn	−68.1 ± 7.4
**3eb**	3-aminocarbonylpropyl	-Bn	−56.5 ± 8.1
**3f**	3-aminocarbonylpropyl	-*t*Bu	−66.0 ± 4.3
**3g**	3-aminocarbonylpropyl	-Cy	−69.5 ± 7.7
**3h**	4-[(2*S*)-2-aminopropyl]phenol	-Bn	−58.4 ± 5.0

**Table 2:** Results of the MST binding check assays.

Compound	Ligand Concentration Displaying:	
	Binding	No Binding
**3a**	500 nM	250 nM
**3b**	5 µM	1 µM
**3c**	4 µM	1 µM
**3d**	20 µM	5 µM
**3ea**	500 nM	250 nM
**3eb**	3.1 µM	1.2 µM
**3f**	1 µM	500 nM
**3g**	5 µM	1 µM
**3h**	100 µM	20 µM
**3d-pin**	/	100 µM
**3f-pin**	20 µM	4 µM

**Table 3:** Evaluation of the inhibitory activity of **3a** and **3ae** against SARS-CoV-2 Proteases.

Compound	Structure	SARS-CoV-2 M^pro^	SARS-CoV-2 PL^pro^
**3a**	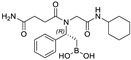	23.39%	n.i.
**3ea**	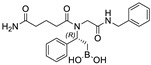	23.70%	n.i.
Nirmaltrevir	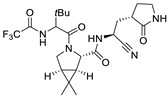	0.003 ± 0.0004 μM ^a^	-

Notes: % of inhibition of the enzymatic activity at 20 μM; ^a^ IC_50_ was determined for Nirmaltrevir; IC_50_ value includes standard deviation from two independent experiments, each performed in triplicate; n.i. = no inhibition.

## Data Availability

Supporting data including NMR spectral charts are available from the Supplementary Materials.

## Supplementary Materials

The following supporting information can be downloaded at https://www.mdpi.com/article/10.3390/molecules28052356/s1, Figure S1, MST binding check experiment of β-amido boronic compounds reported in Table 2. Figure S2, MST binding check experiment of β-amido boronic compounds protected by the pinacol group. Figure S3, MST analysis reporting the binding affinity assay of 3ea on 6His Control Peptide. Figure S4, MST binding check experiments to determine whether the 3ea compound binds reversibly or irreversibly to the Mpro^Cov-2^. Figure S5, RMSD/time plots of β-amido boronic compounds reported in Table 1. Figure S6, LC-MS/MS experiment on a β-amido boronic acid. Figure S7, HPLC chromatograms for compounds 3a and 3ea. Copies of 1H, 13C, and 11B NMR spectra.

## Abbreviations

MD: Molecular dynamics; RMSD root mean square deviation; MM-GBSA, Molecular Mechanics-Generalized Born Surface Area.
