# Supplementary material for: Computational Design, Synthesis, and Biophysical Evaluation of β-Amido Boronic Acids as SARS-CoV-2 Mpro Inhibitors
Source: Molecules. 2023 Mar 3;28(5):2356. doi: 10.3390/molecules28052356 (PMC10005264; doi:10.3390/molecules28052356)

Electronic supporting information *for*

Computational Design, Synthesis, and Biophysical Evaluation of β-Amido Boronic Acids as SARS-CoV-2 M^{pro} Inhibitors

Enrico M. A. Fassi, Marco Manenti, Andrea Citarella, Michele dei Cas, Sara Casati, Nicola Micale, Tanja Schirmeister, Gabriella Roda, Alessandra Silvani, and Giovanni Grazioso

Table of contents:

- Figure S1
- Figure S2
- Figure S3
- Figure S4
- Figure S5
- Figure S6
- Figure S7
- Copies of ^1H, ^{13}C and ^{11}B NMR spectra

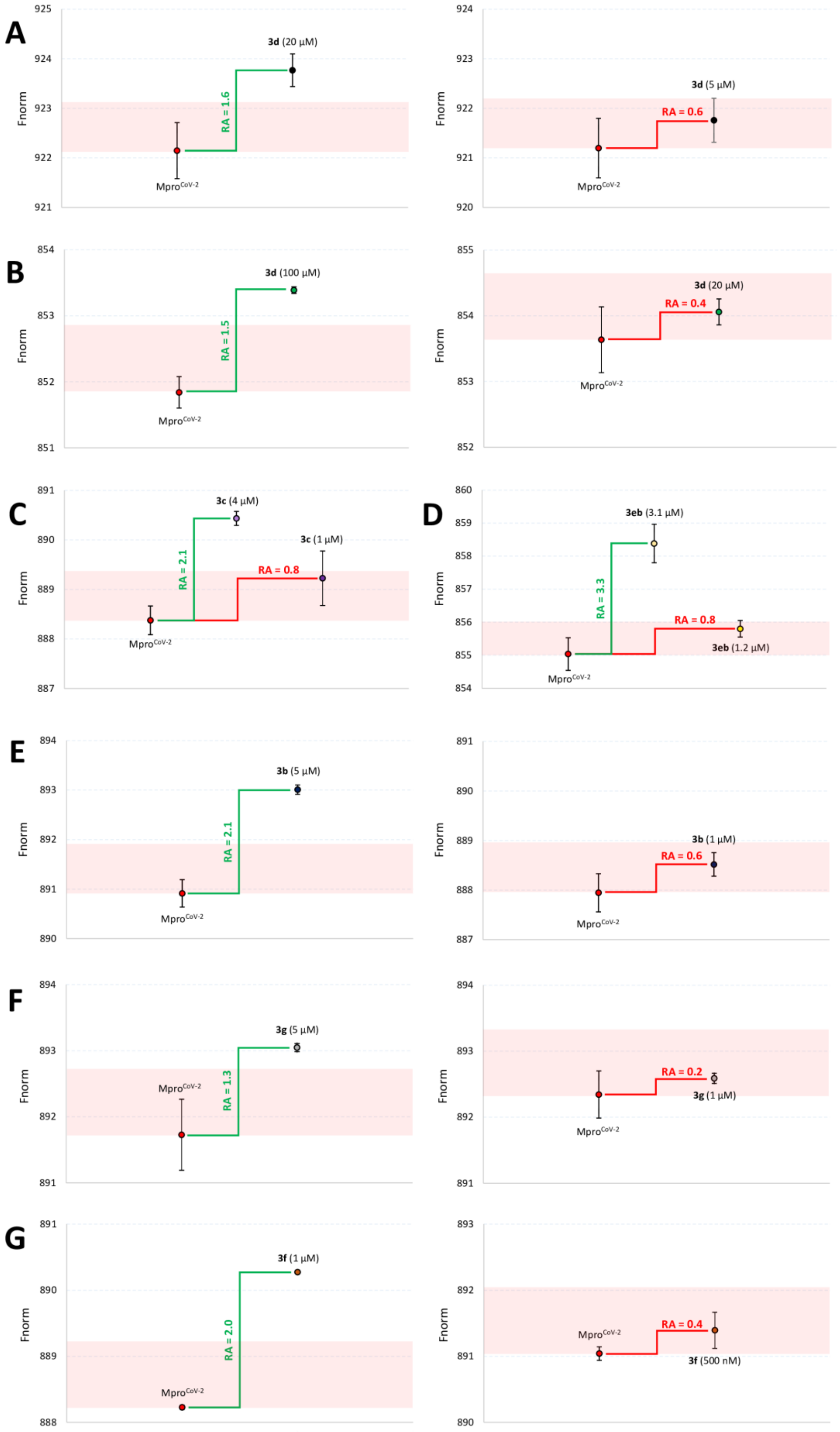

Figure S1. MST binding check experiments of β-amido boronic compounds reported in Table 2 on the manuscript. In red is highlighted the "no binding" Fnorm area due to an insufficient Response Amplitude (RA).

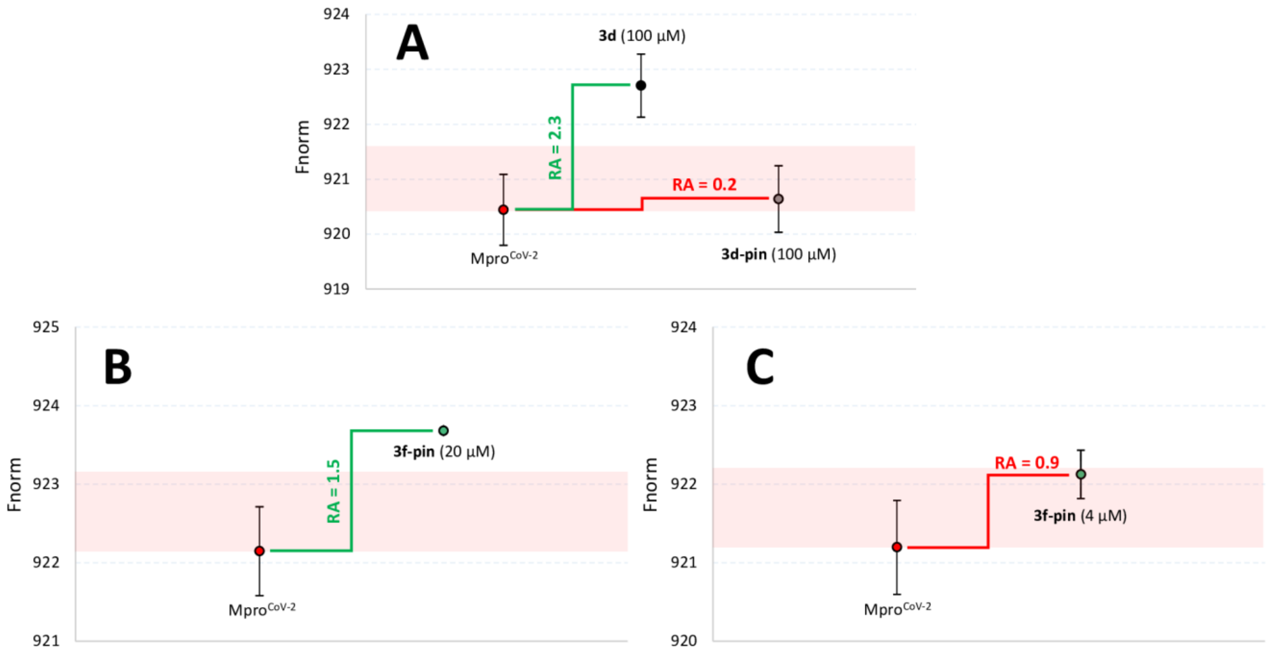

Figure S2. MST binding check experiment of β-amido boronic compounds protected by the pinacol group. (A) Comparison between **3d** and **3d-pin** at a concentration of 100 µM. (B-C) **3f-pin** at a concentration of 20 µM and 4 µM, respectively. In red is highlighted the “no binding” Fnorm area due to an insufficient Response Amplitude (RA).

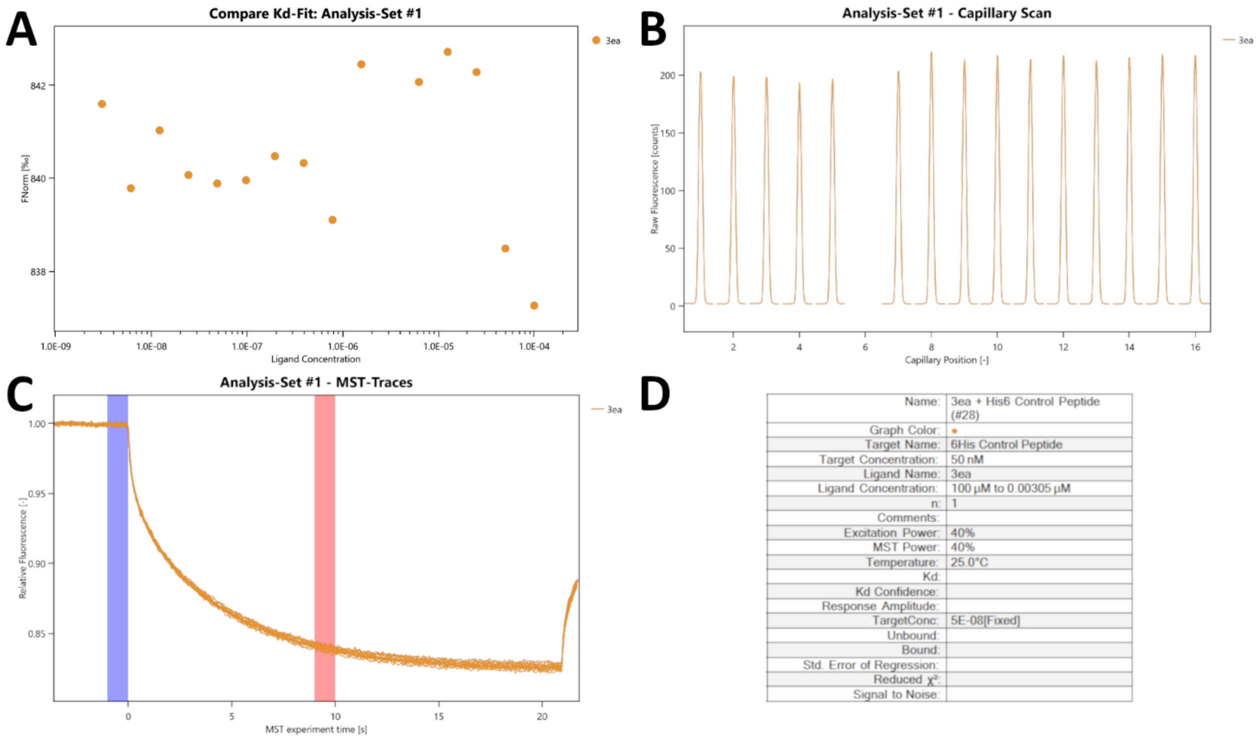

Name:	3ea + His6 Control Peptide (#28)
Graph Color:	•
Target Name:	6His Control Peptide
Target Concentration:	50 nM
Ligand Name:	3ea
Ligand Concentration:	100 µM to 0.00305 µM
n:	1
Comments:	
Excitation Power:	40%
MST Power:	40%
Temperature:	25.0°C
Kd:	
Kd Confidence:	
Response Amplitude:	
TargetConc:	5E-08[Fixed]
Unbound:	
Bound:	
Std. Error of Regression:	
Reduced χ^2:	
Signal to Noise:	

Figure S3. MST analysis reporting the binding affinity assay of **3ea** on 6His Control Peptide. (A) F_{norm} values, (B) raw fluorescence values and (C) MST traces observed for each capillary. (D) Dataset overview. The 6th point, corresponding to a concentration of 3.1 µM, was discarded since it appears to be a clear outlier.

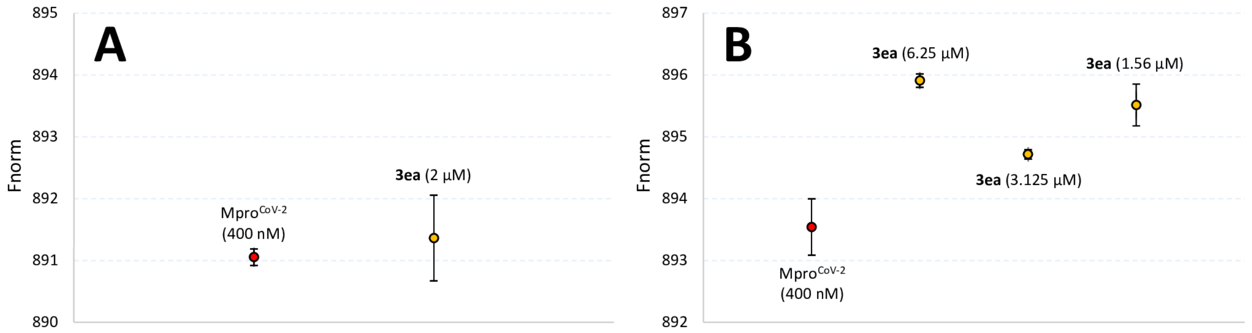

Figure S4. MST binding check experiments to determine whether if **3ea** compound binds reversibly or irreversibly to the Mpro^{Cov-2}. (A) Fnorm values of protein alone compared to **3ea**(2µM)/Mpro^{Cov-2} complex. (B) Fnorm values of protein alone compared to **3ea**(6.25µM)/protein complex and its dilutions.

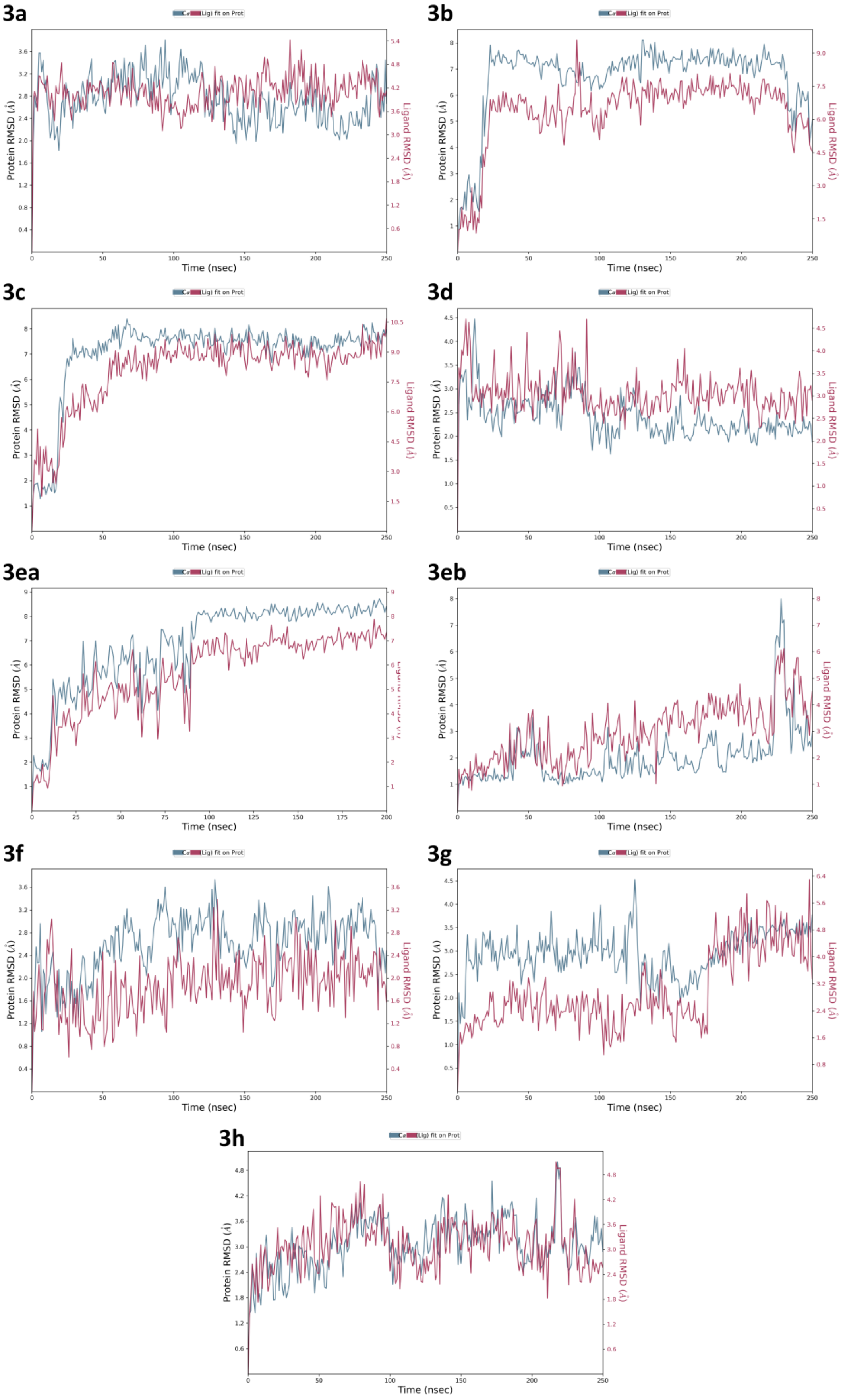

Figure S5. RMSD versus time plots of the synthesized β-amido boronic compounds reported in Table 1 in the main text. All the compounds were subjected to a 250 ns-long MD simulations.

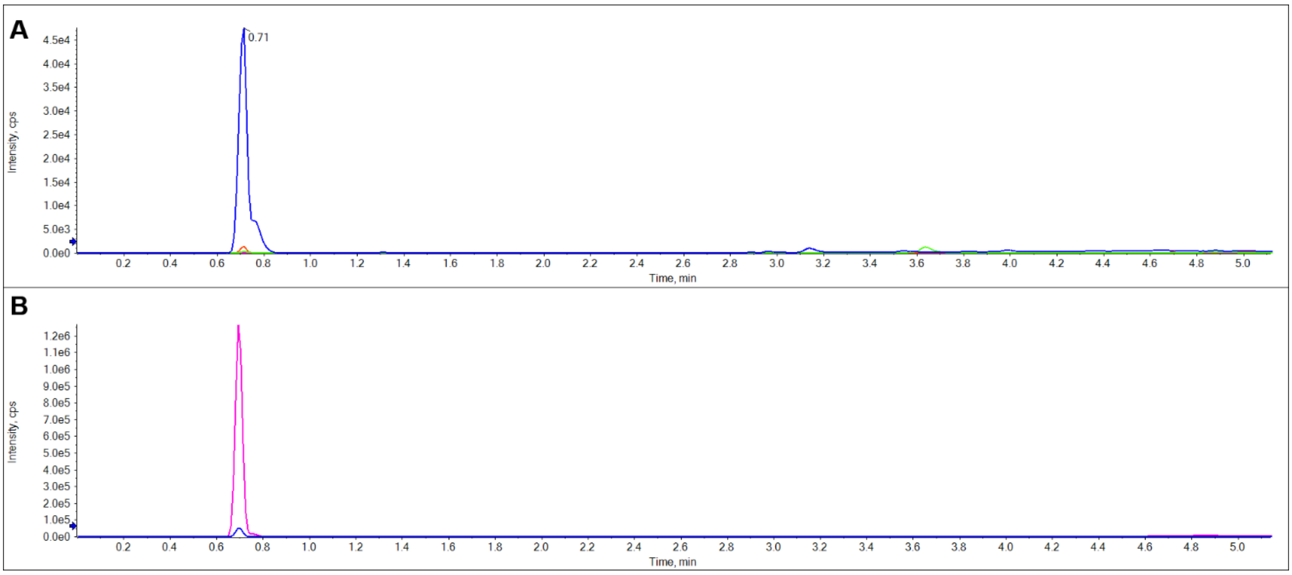

Figure S6. LC-MS/MS experiment on a β-amido boronic acid. (A) Extracted ion chromatograms of **3ea** [M+H]+ and (B) **3ea** $[M+2H]^{2+}$.

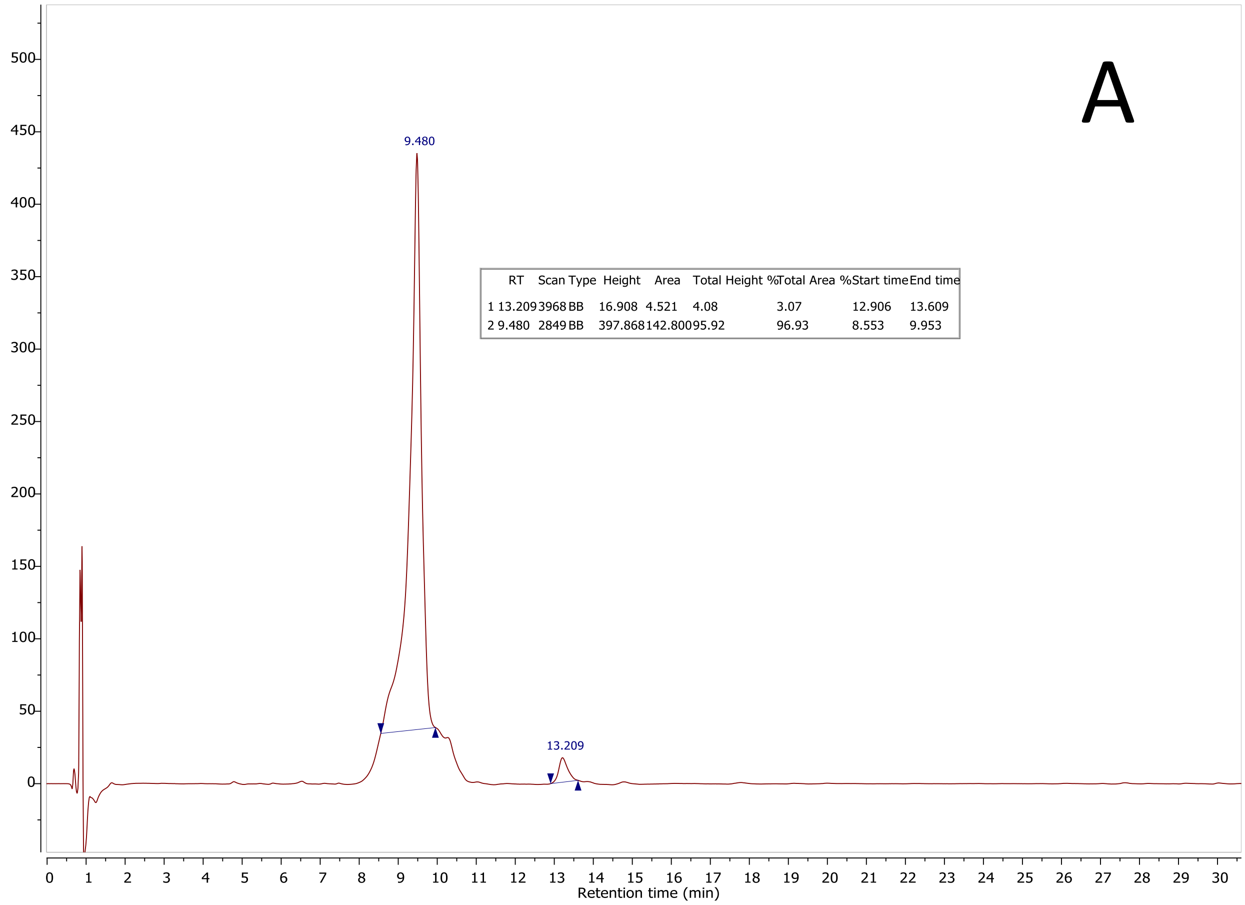

	RT	Scan	Type	Height	Area	Total Height %	Total Area %	Start time	End time
1	13.209	3968	BB	16.908	4.521	4.08	3.07	12.906	13.609
2	9.480	2849	BB	397.868	142.800	95.92	96.93	8.553	9.953

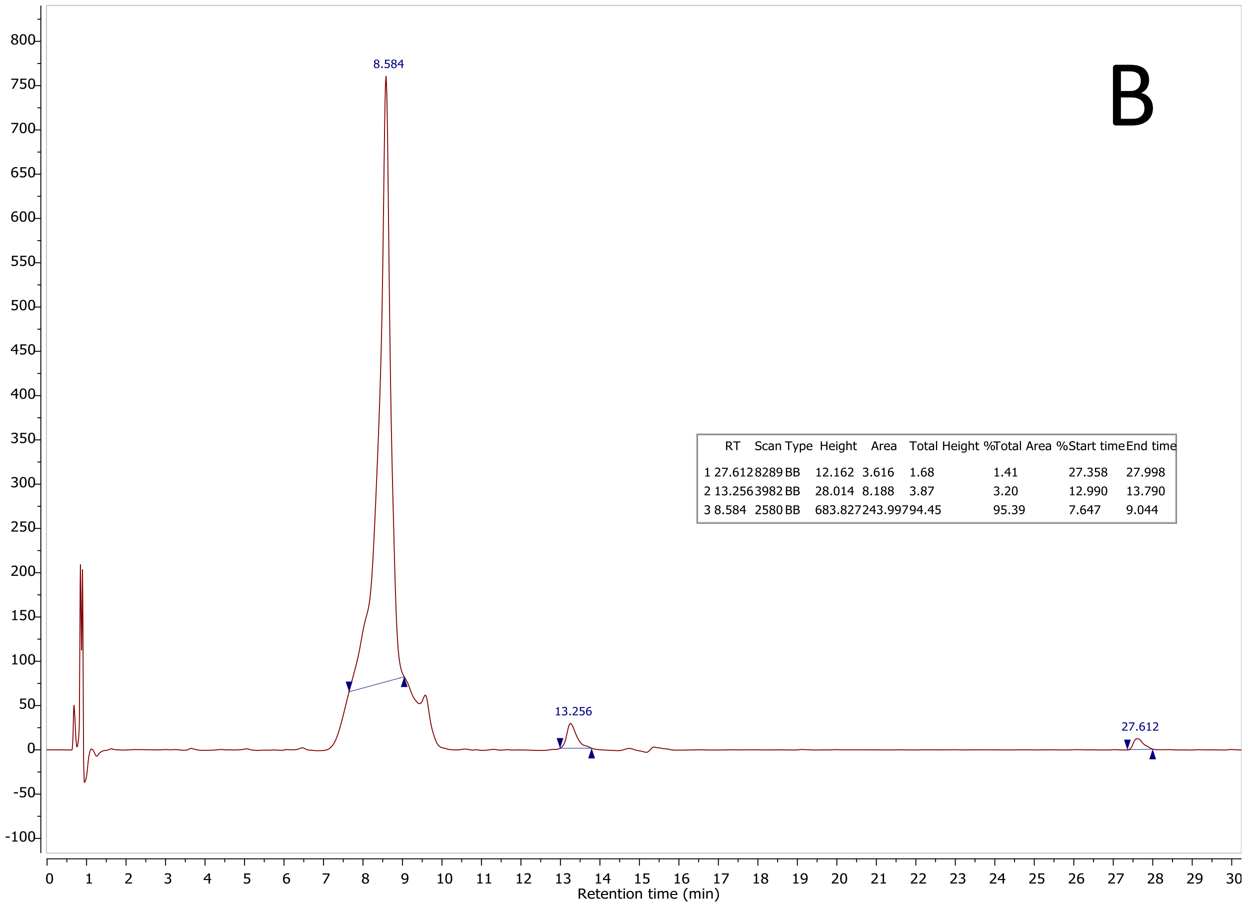

Figure S7. HPLC chromatograms for compounds 3a (panel A) and 3ea (panel B). HPLC was performed on Agilent 1100 Series System using a Gemini 5 µM C18 110 Å LC Column 150 × 3 mm and a gradient of H_2O/ACN from 5% ACN to 100% ACN in 30 min, flux of 1.0 mL/min and sample injection of 20 µL. The wavelength used for detection was 220 nm.

^{1}H NMR of compound **2a** (400 MHz, $CDCl_3$ 55:45 rotameric mixture)

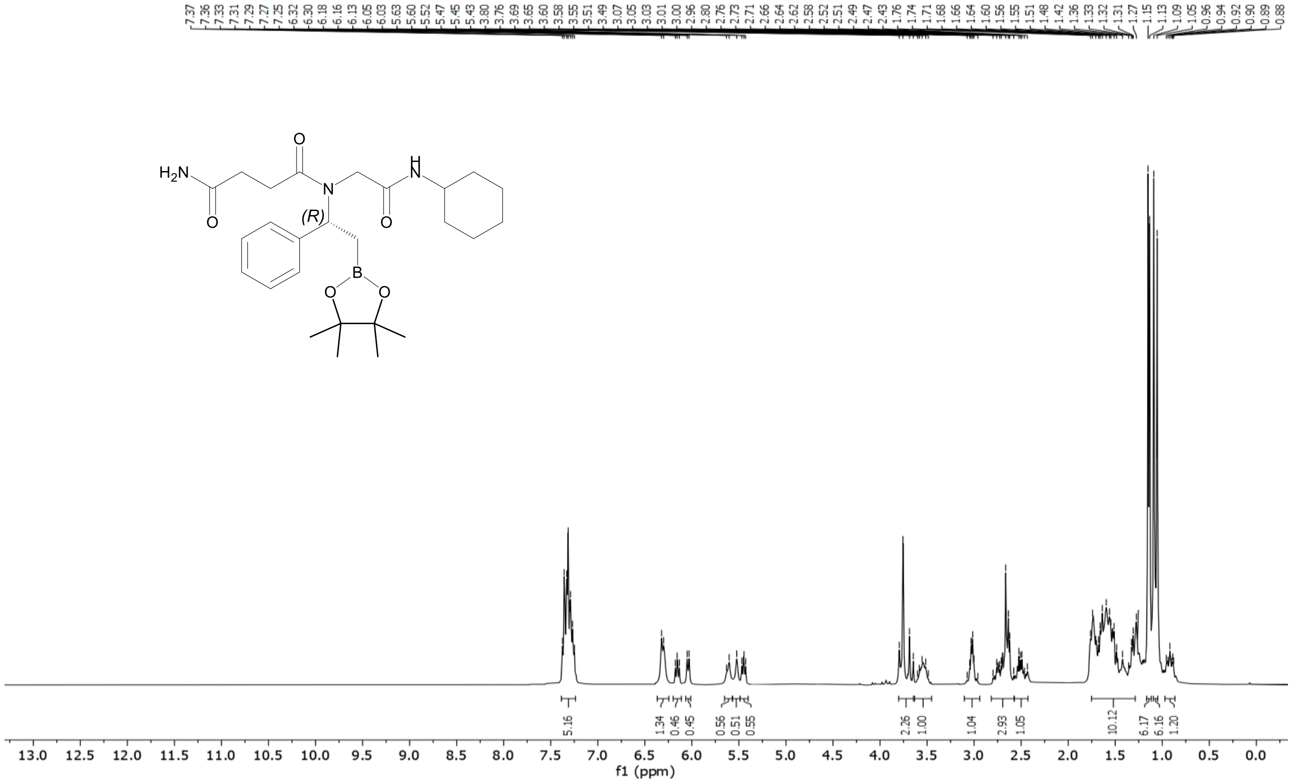

^{13}C NMR of compound **2a** (101 MHz, $CDCl_3$ 55:45 rotameric mixture)

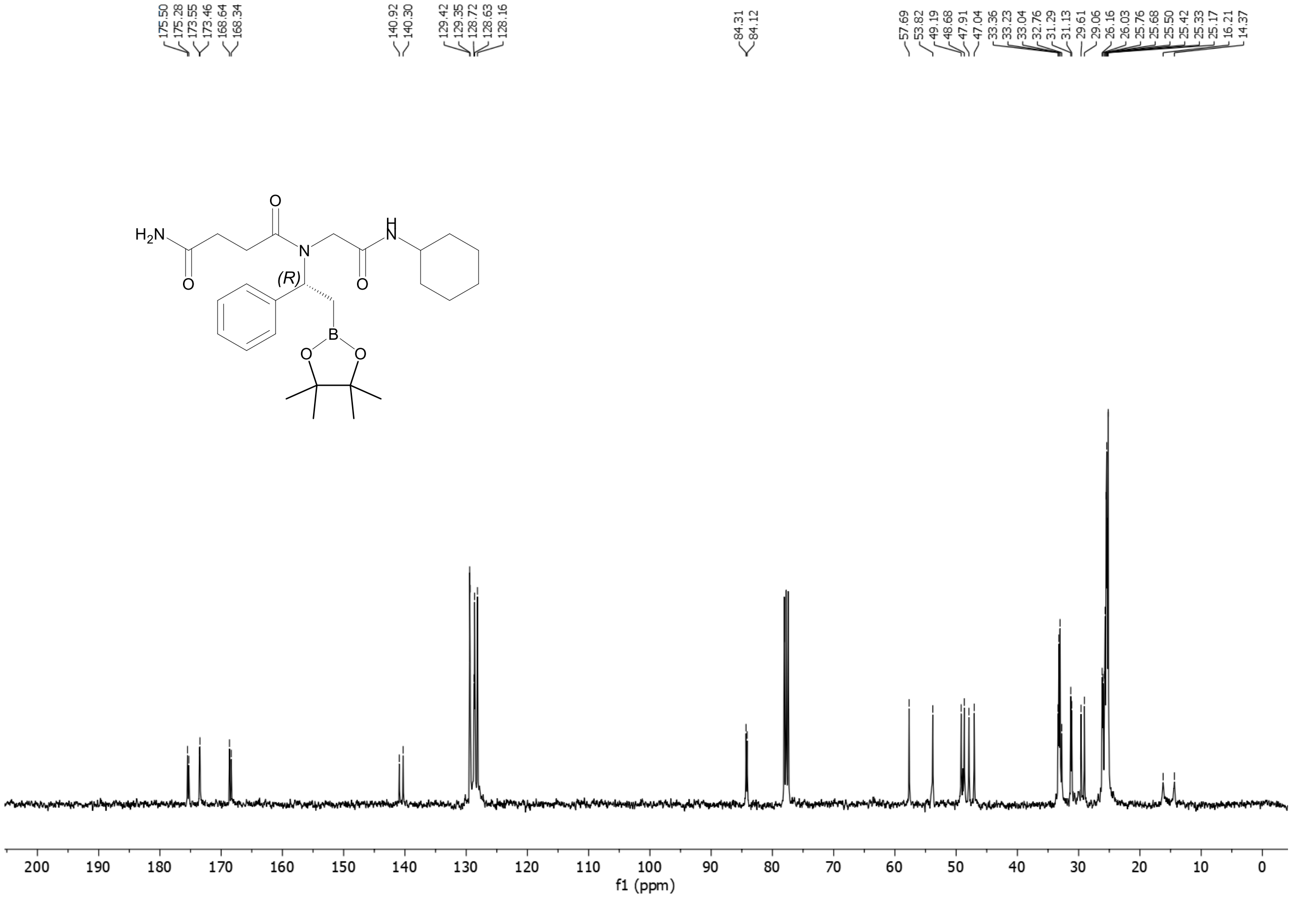

^{11}B NMR of compound **2a** (128 MHz, $CDCl_3$)

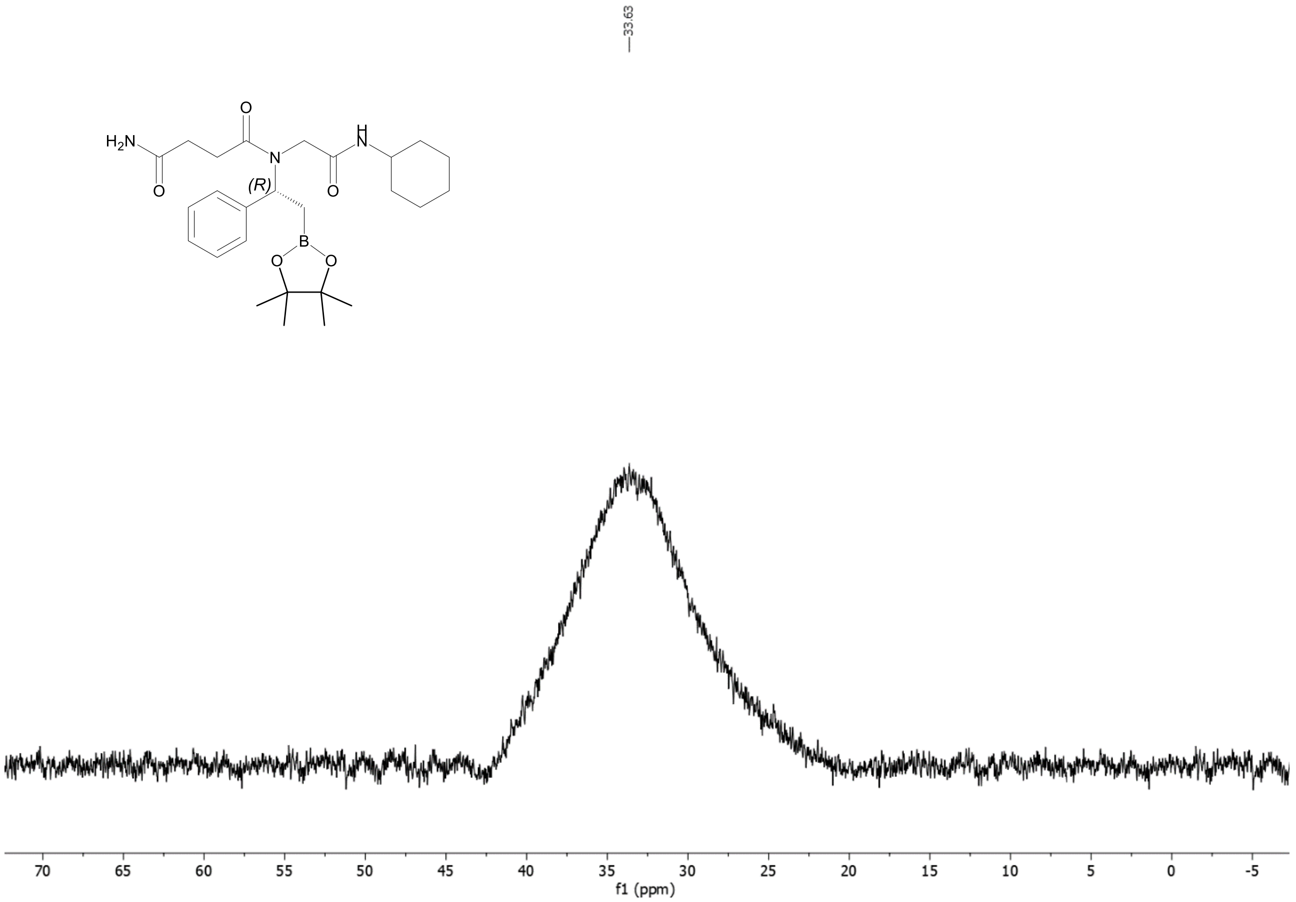

^{1}H NMR of compound **2b** (400 MHz, $CDCl_3$, 50:50 rotameric mixture)

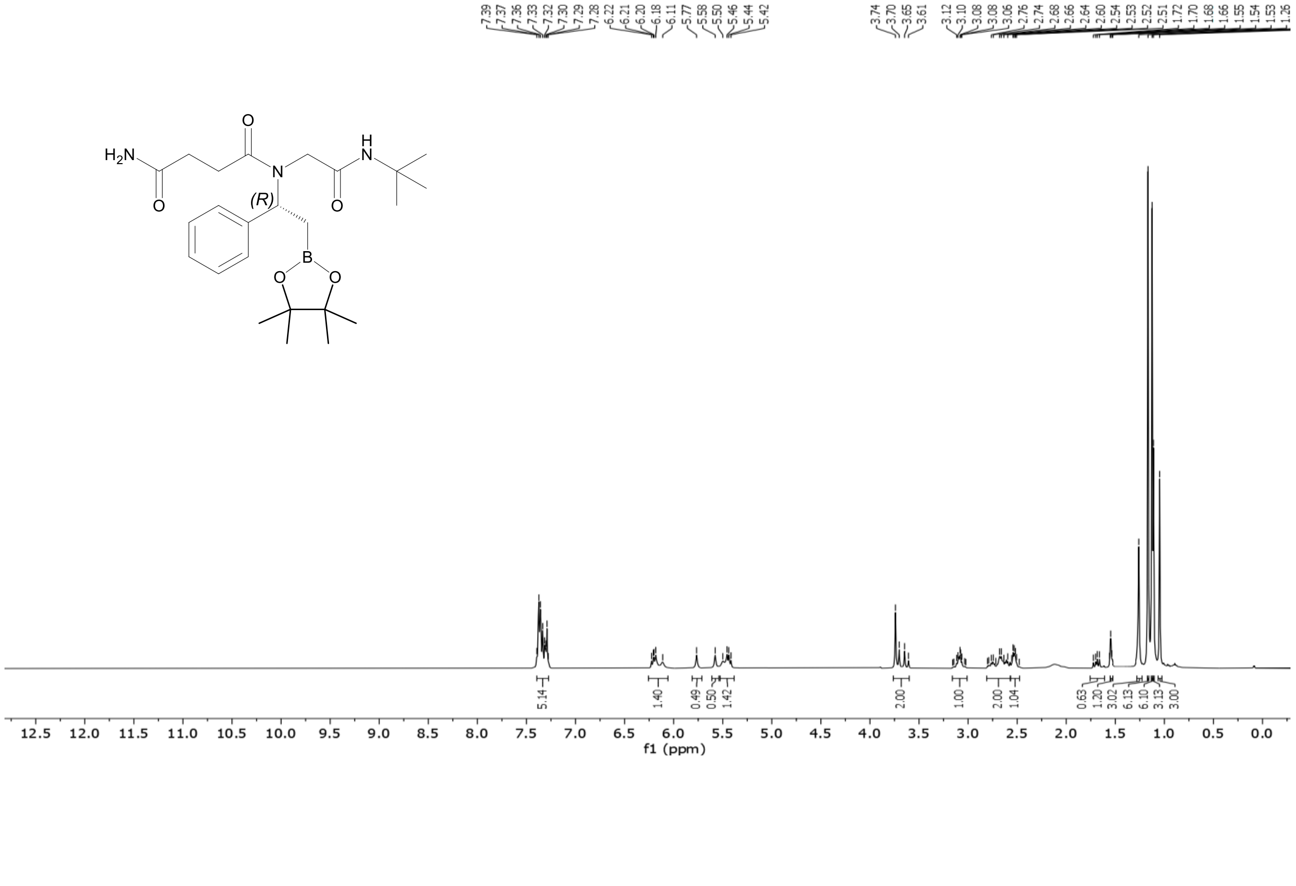

^{13}C NMR of compound **2b** (101 MHz, $CDCl_3$ 50:50 rotameric mixture)

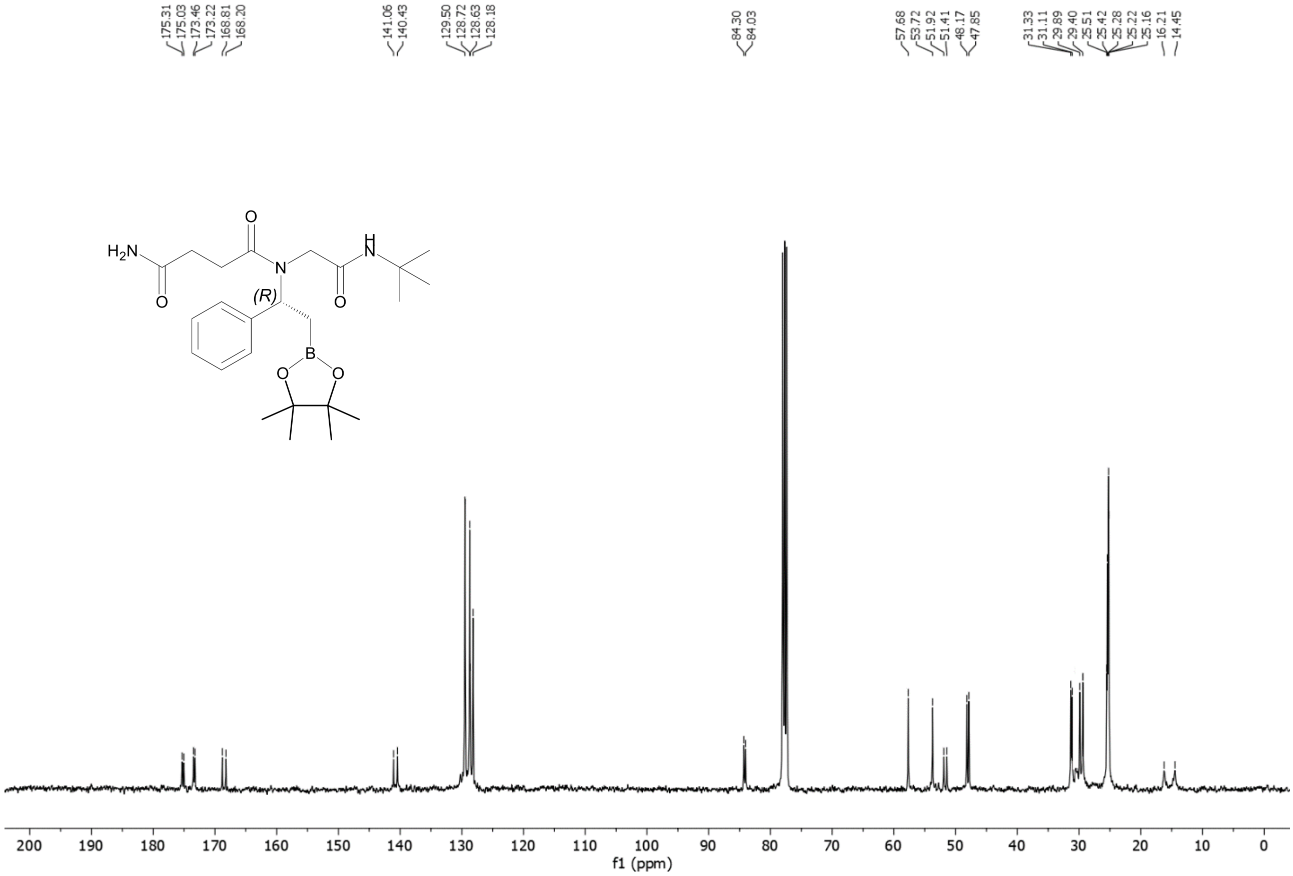

^{11}B NMR of compound **2b** (128 MHz, $CDCl_3$)

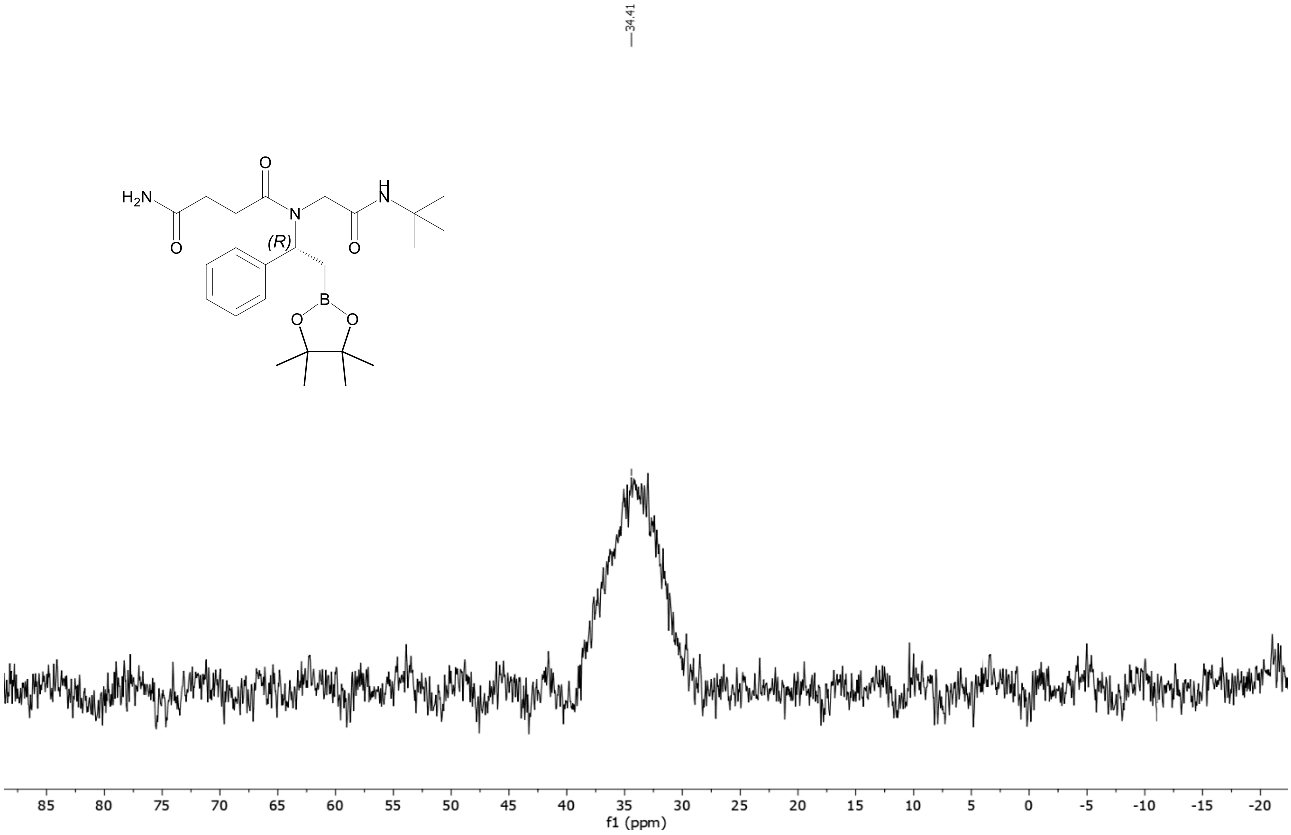

^{1}H NMR of compound **2c** (400 MHz, $CDCl_3$ 65:35 rotameric mixture)

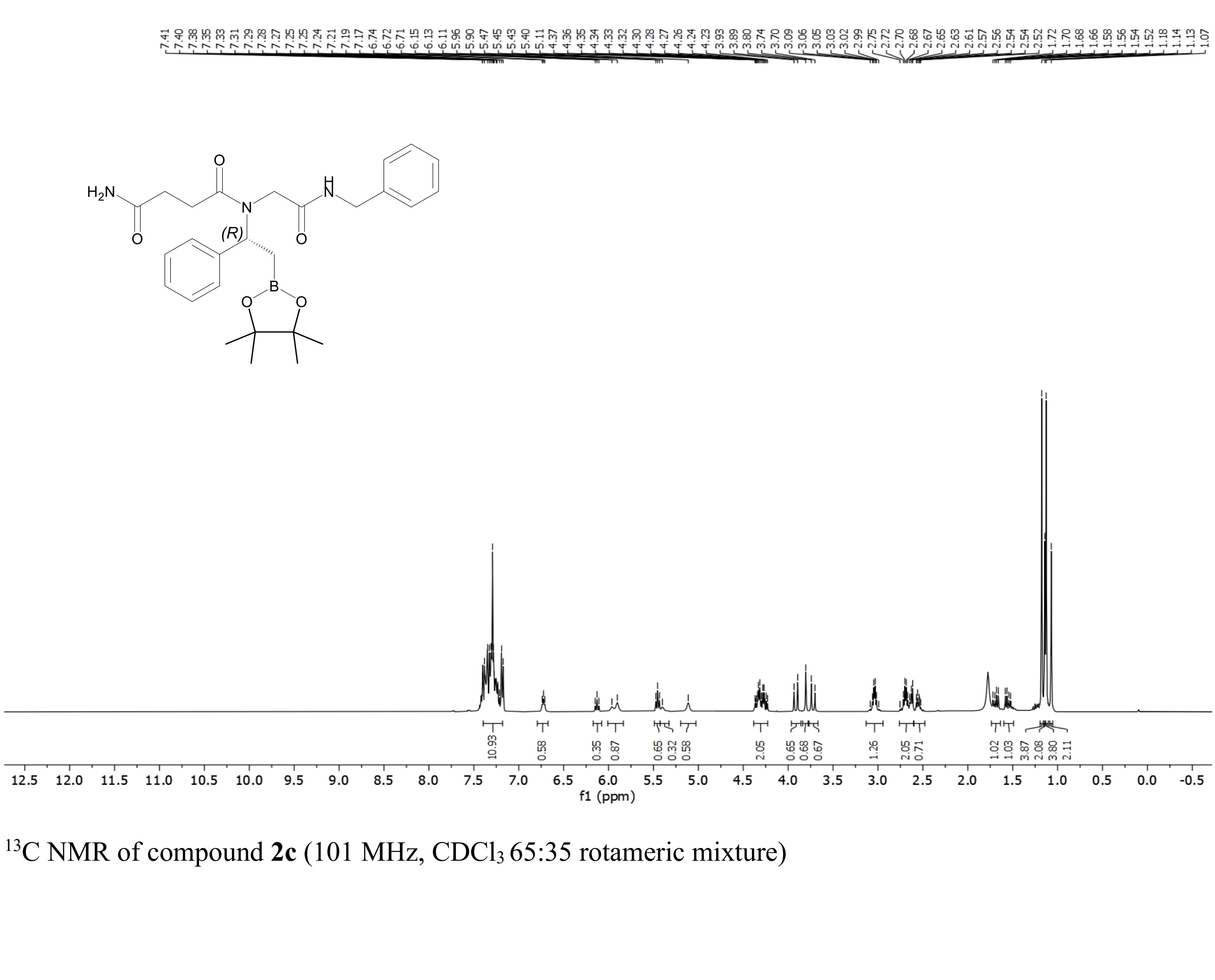

^{13}C NMR of compound **2c** (101 MHz, $CDCl_3$ 65:35 rotameric mixture)

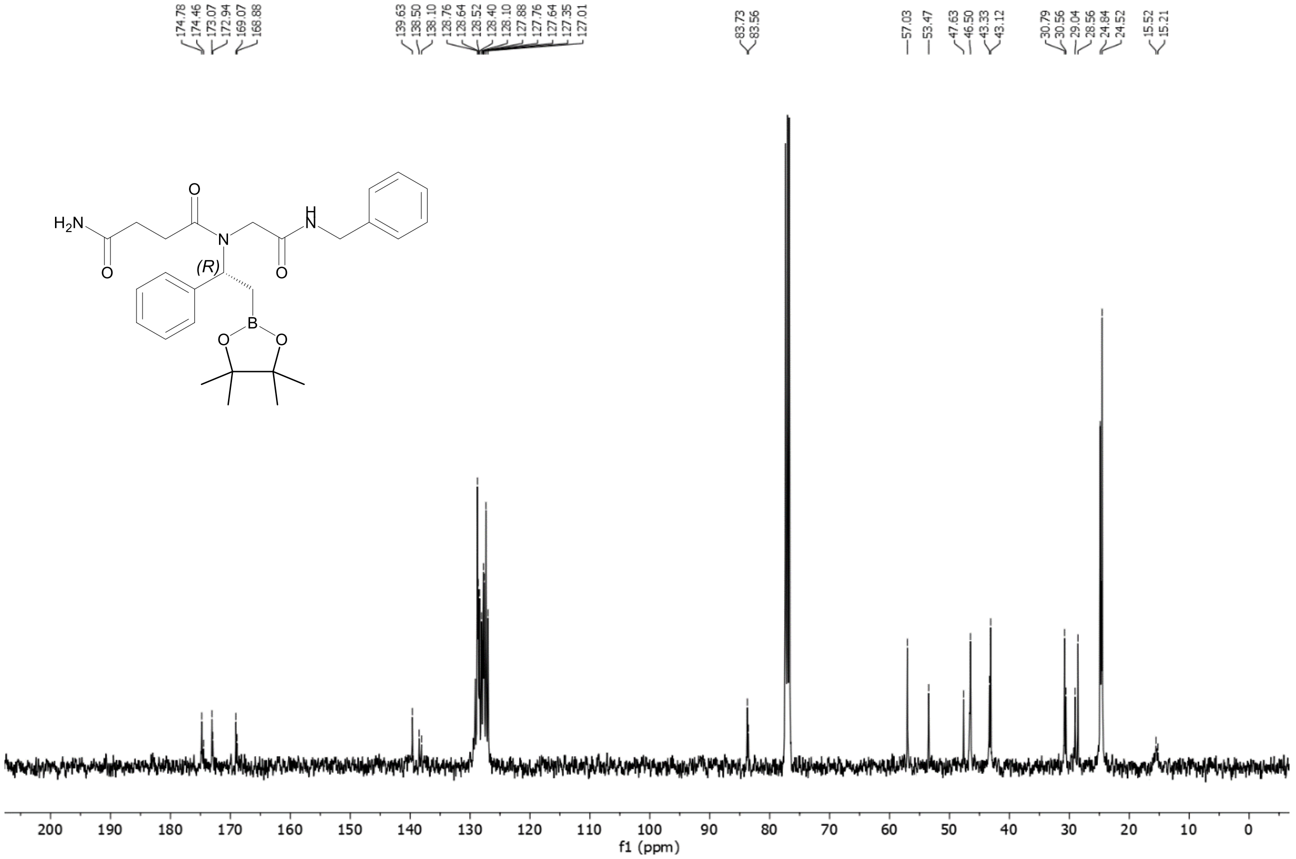

^{11}B NMR of compound **2c** (128 MHz, $CDCl_3$)

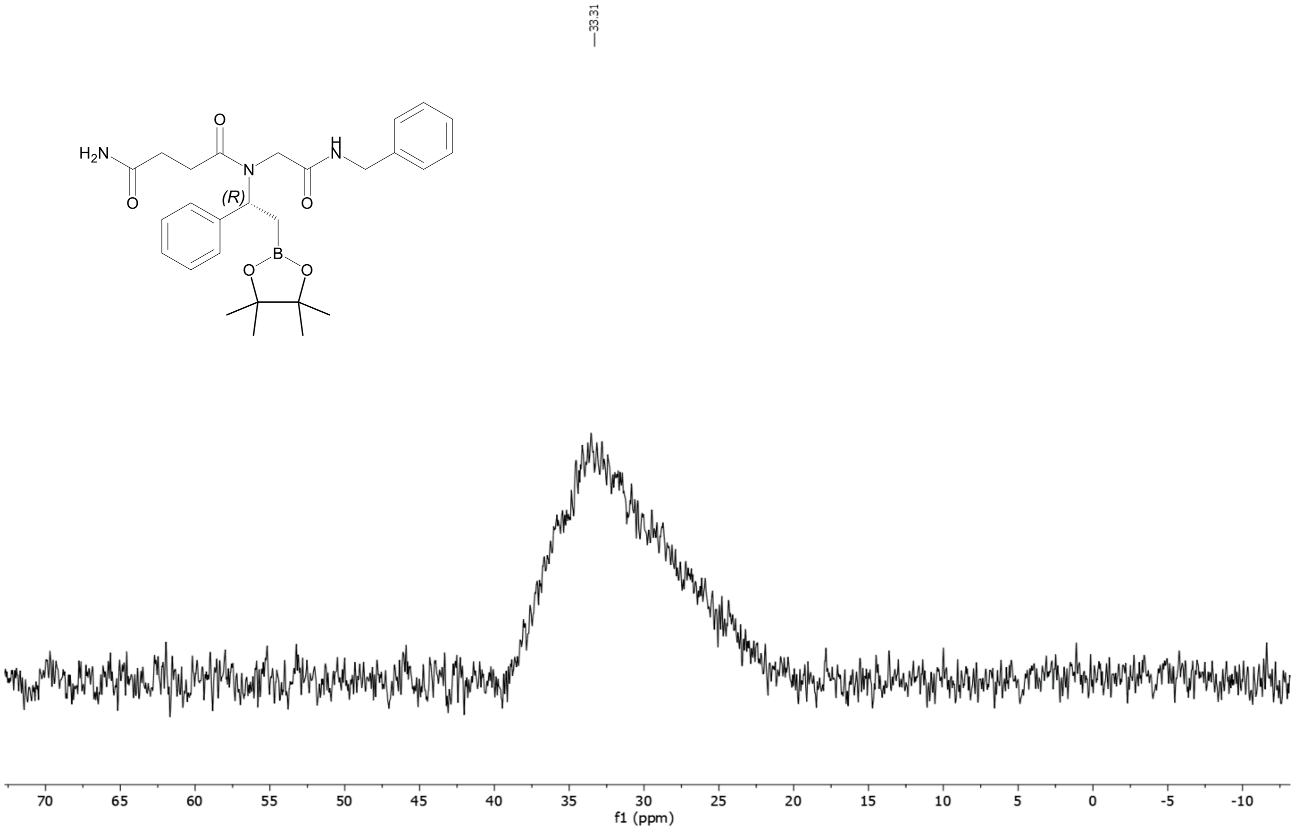

^{1}H NMR of compound **2d** (400 MHz, $CDCl_3$, complex rotameric mixture: the section by section integration proves the overall number of protons)

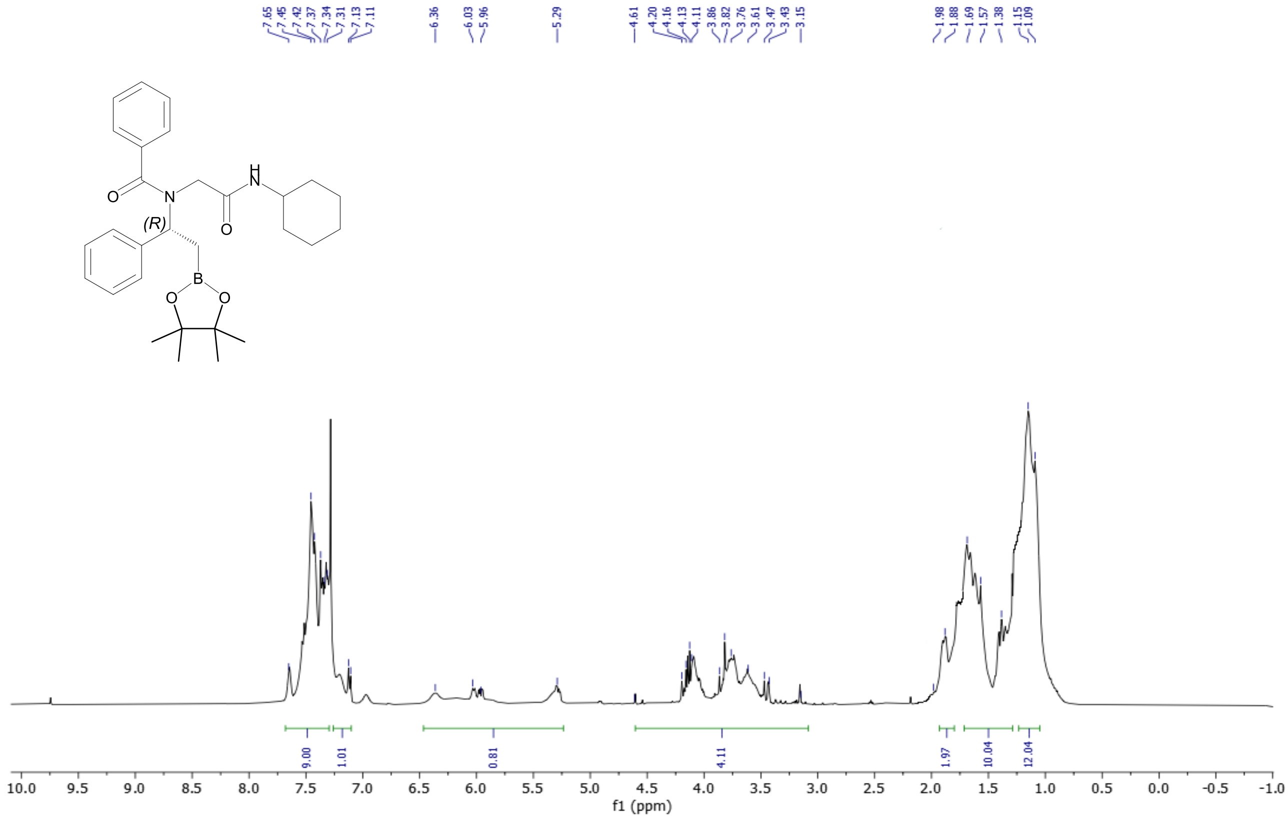

^{13}C NMR of compound **2d** (101 MHz, $CDCl_3$, complex rotameric mixture)

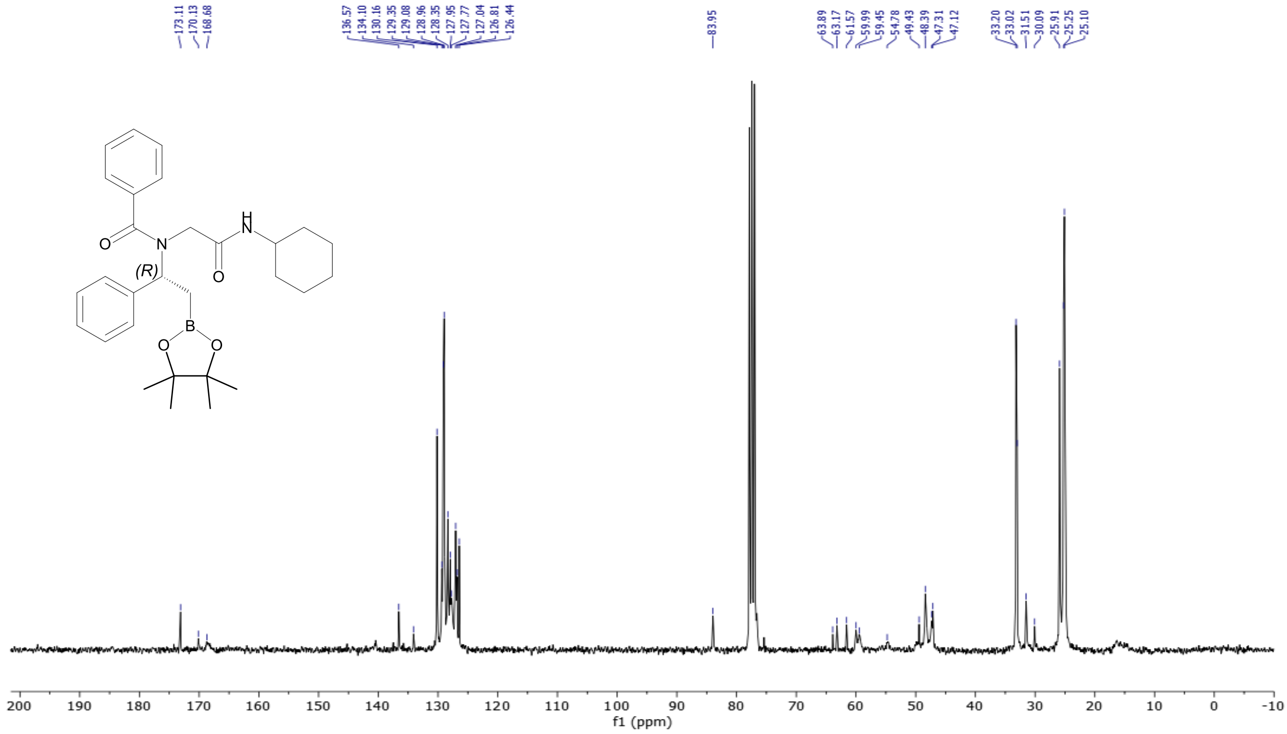

^{11}B NMR of compound **2d** (128 MHz, $CDCl_3$)

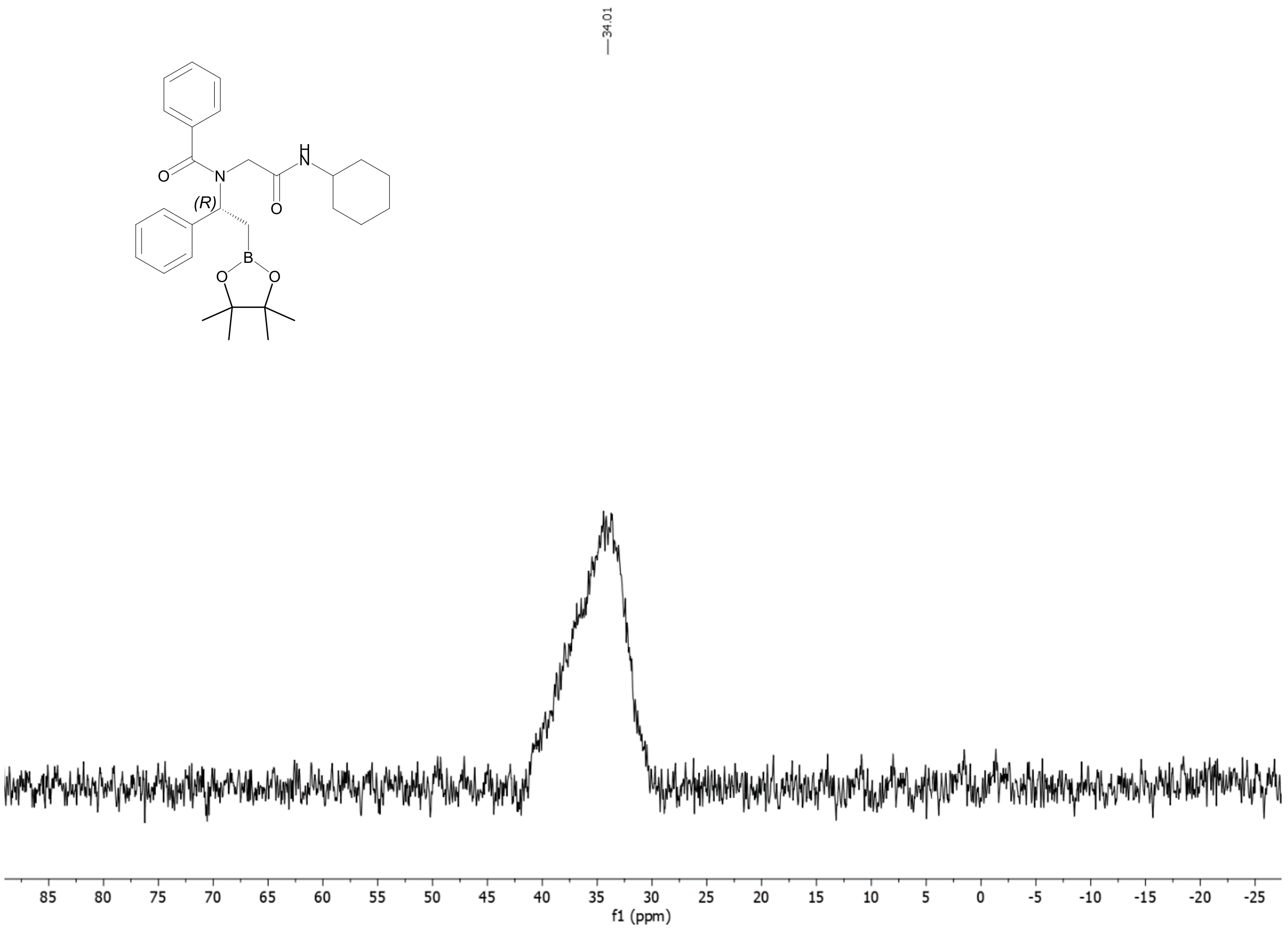

^{1}H NMR of compound **2ea** (400 MHz, $CDCl_3$ 60:40 rotameric mixture)

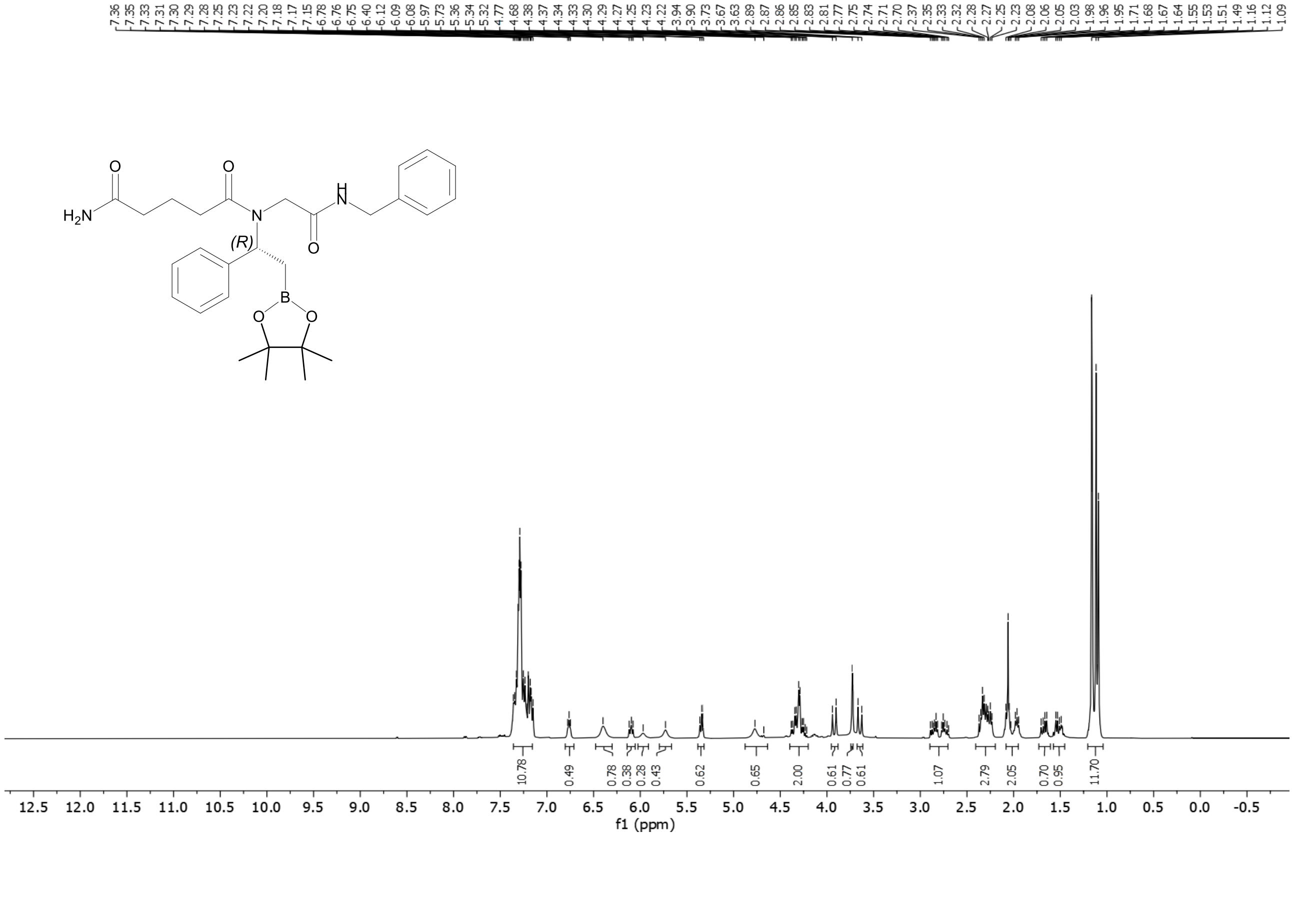

^{13}C NMR of compound **2ea** (101 MHz, $CDCl_3$, 60:40 rotameric mixture)

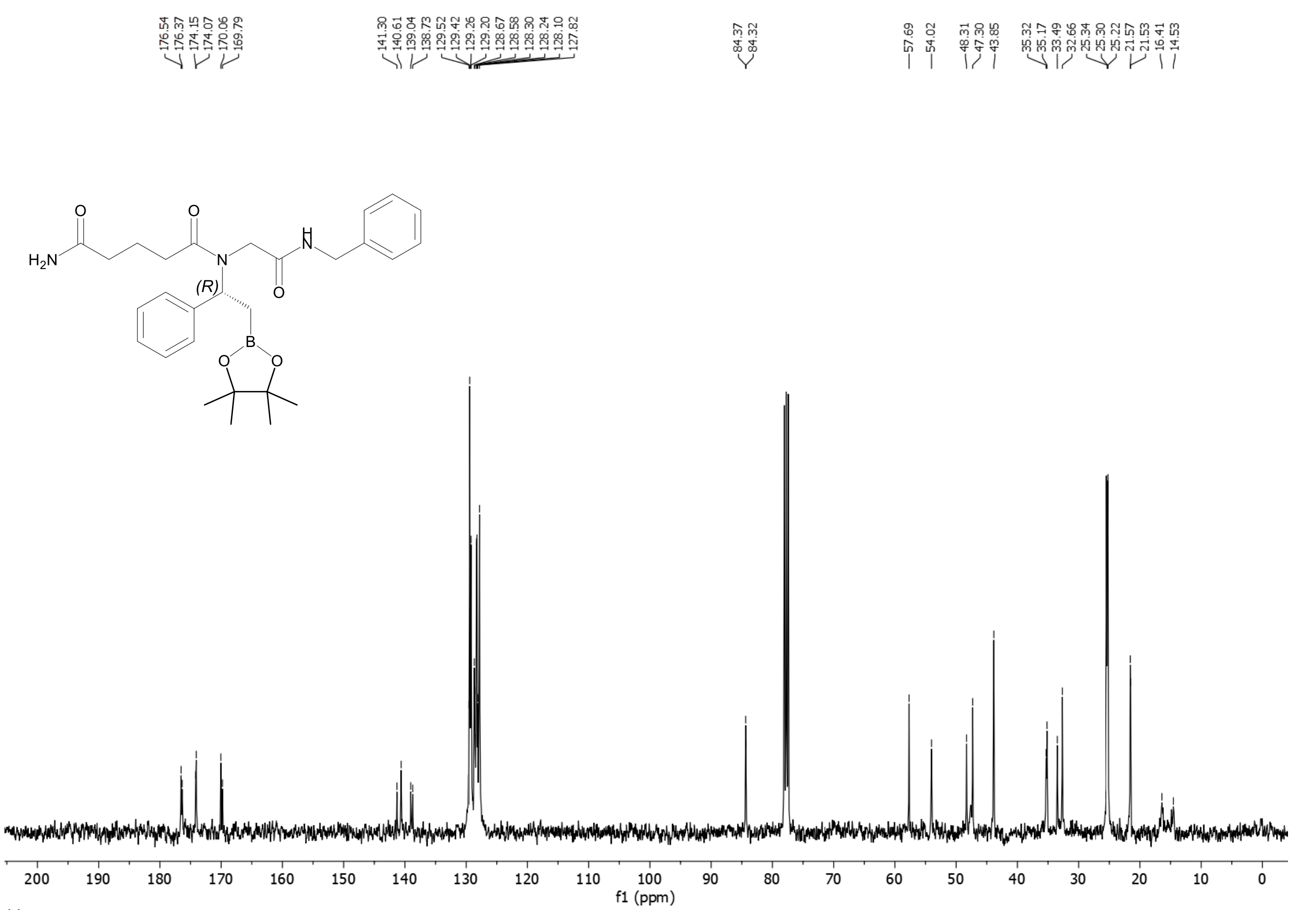

^{11}B NMR of compound **2ea** (128 MHz, $CDCl_3$)

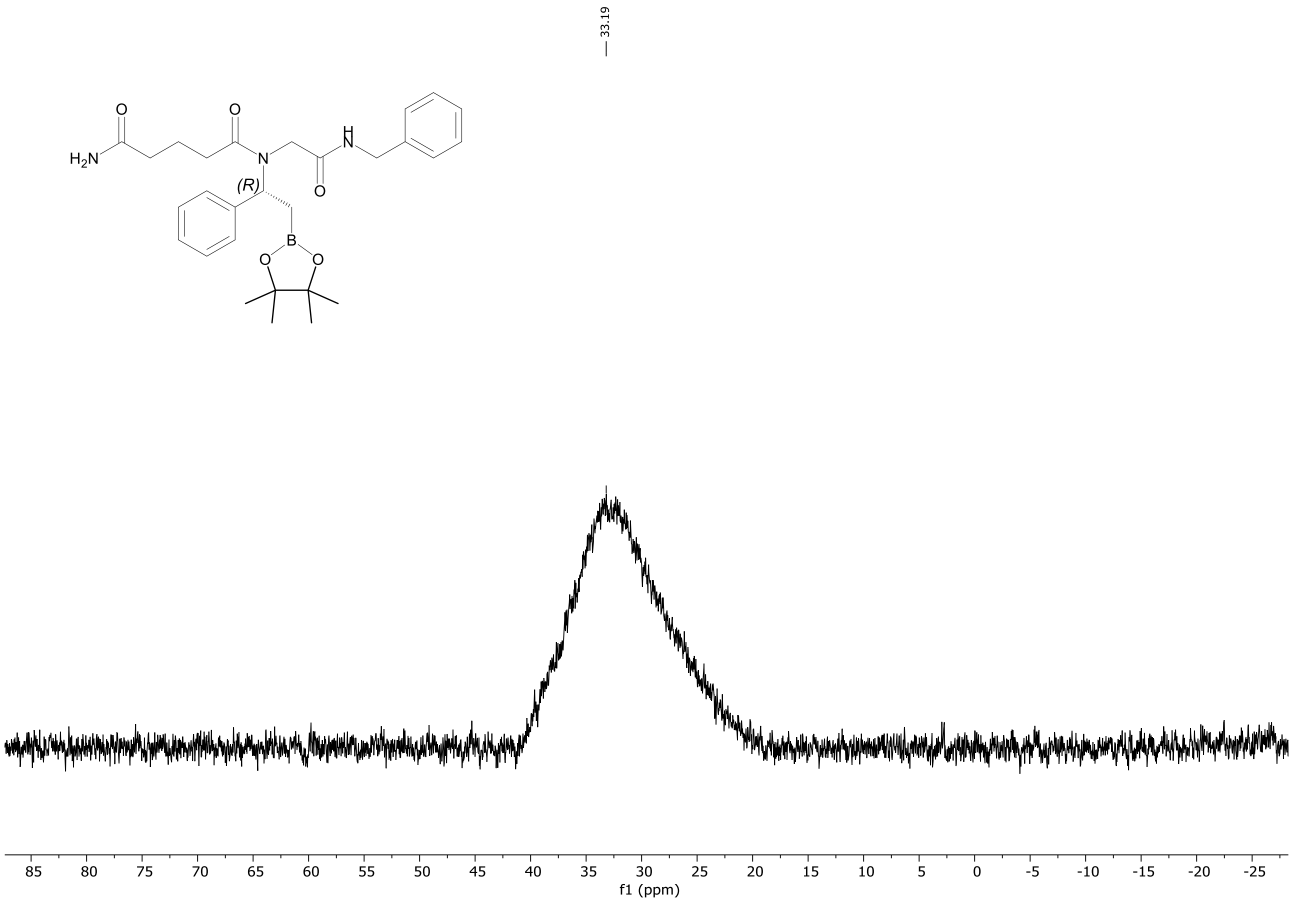

^{1}H NMR of compound **2f** (400 MHz, $CDCl_3$, 55:45 rotameric mixture)

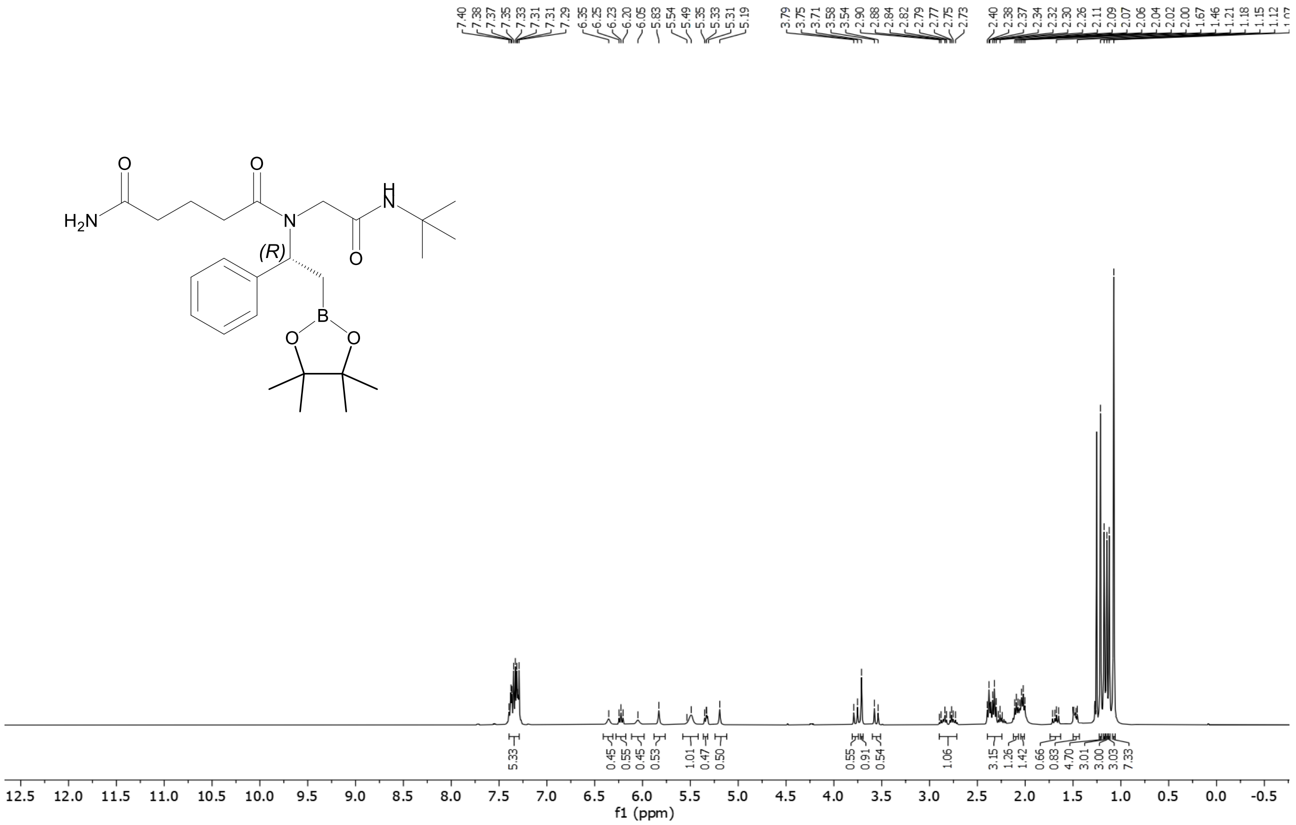

^{13}C NMR of compound **2f** (101 MHz, $CDCl_3$, 55:45 rotameric mixture)

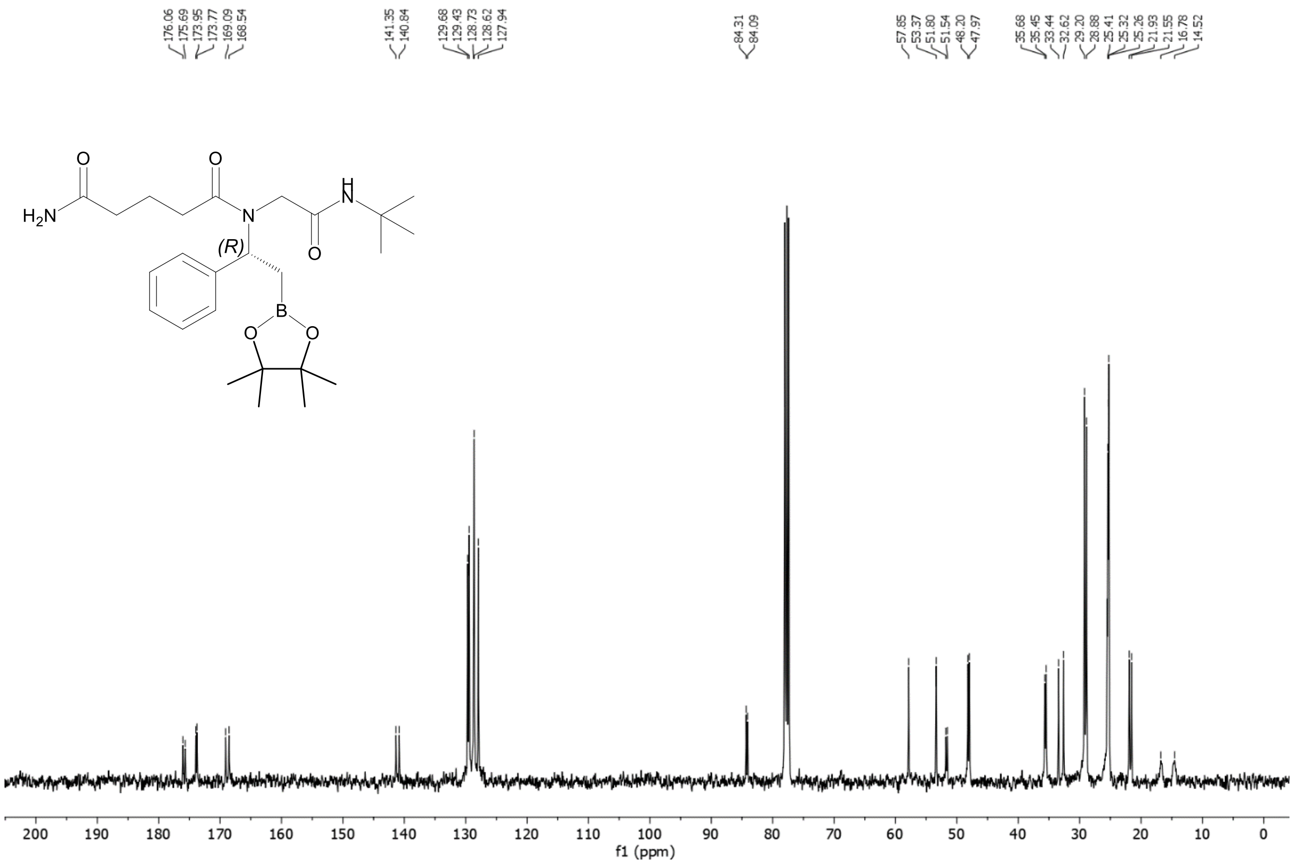

^{11}B NMR of compound **2f** (128 MHz, $CDCl_3$)

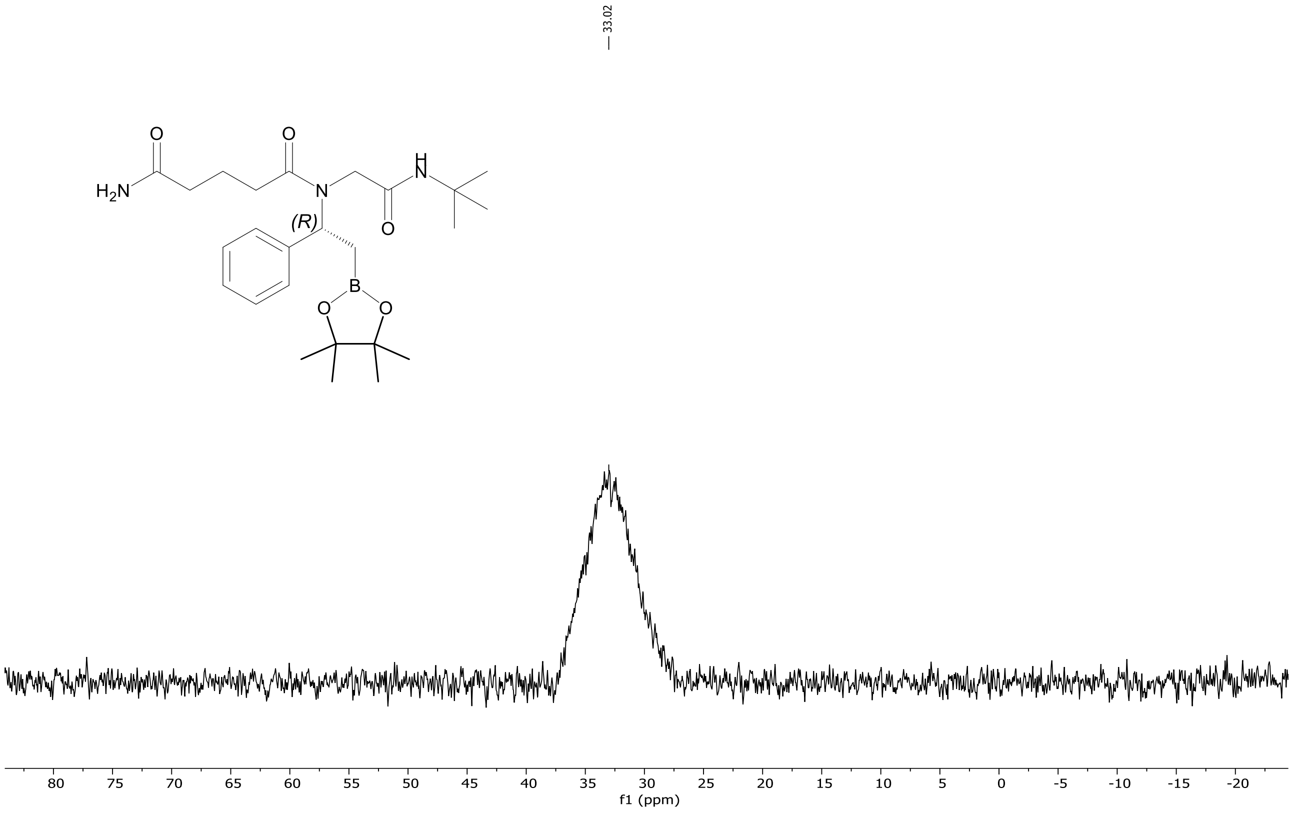

^{1}H NMR of compound **2g** (400 MHz, $CDCl_3$ 65:35 rotameric mixture)

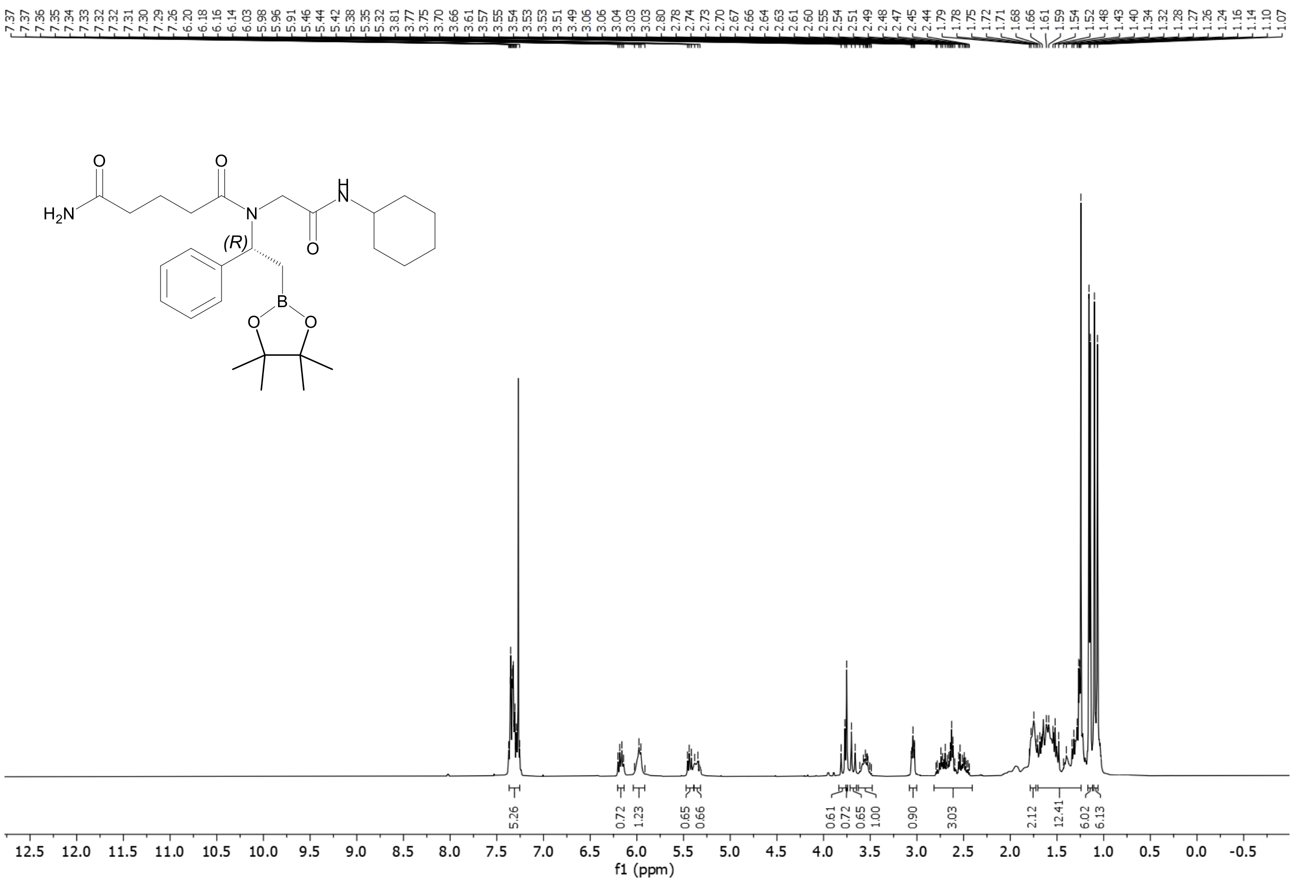

^{13}C NMR of compound **2g** (101 MHz, $CDCl_3$ 65:35 rotameric mixture)

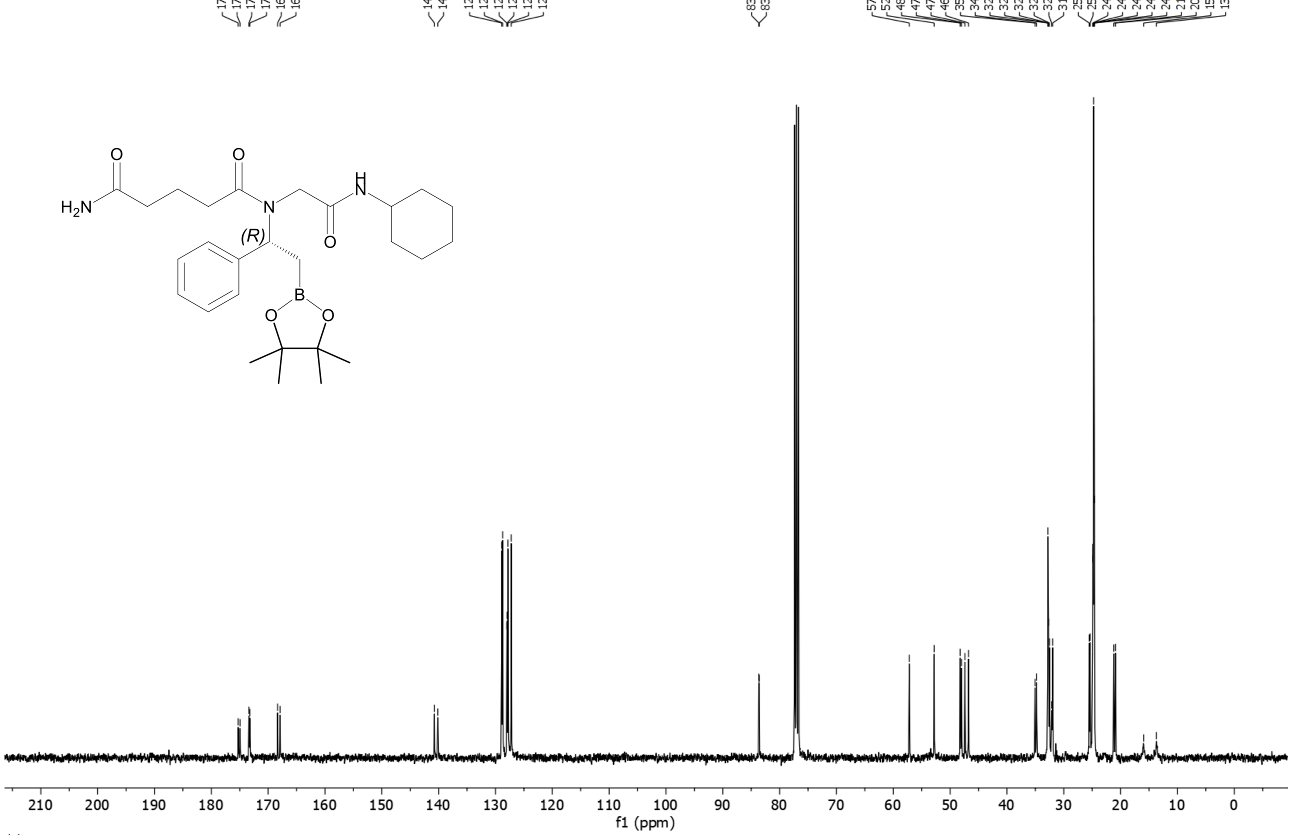

^{11}B NMR of compound **2g** (128 MHz, $CDCl_3$)

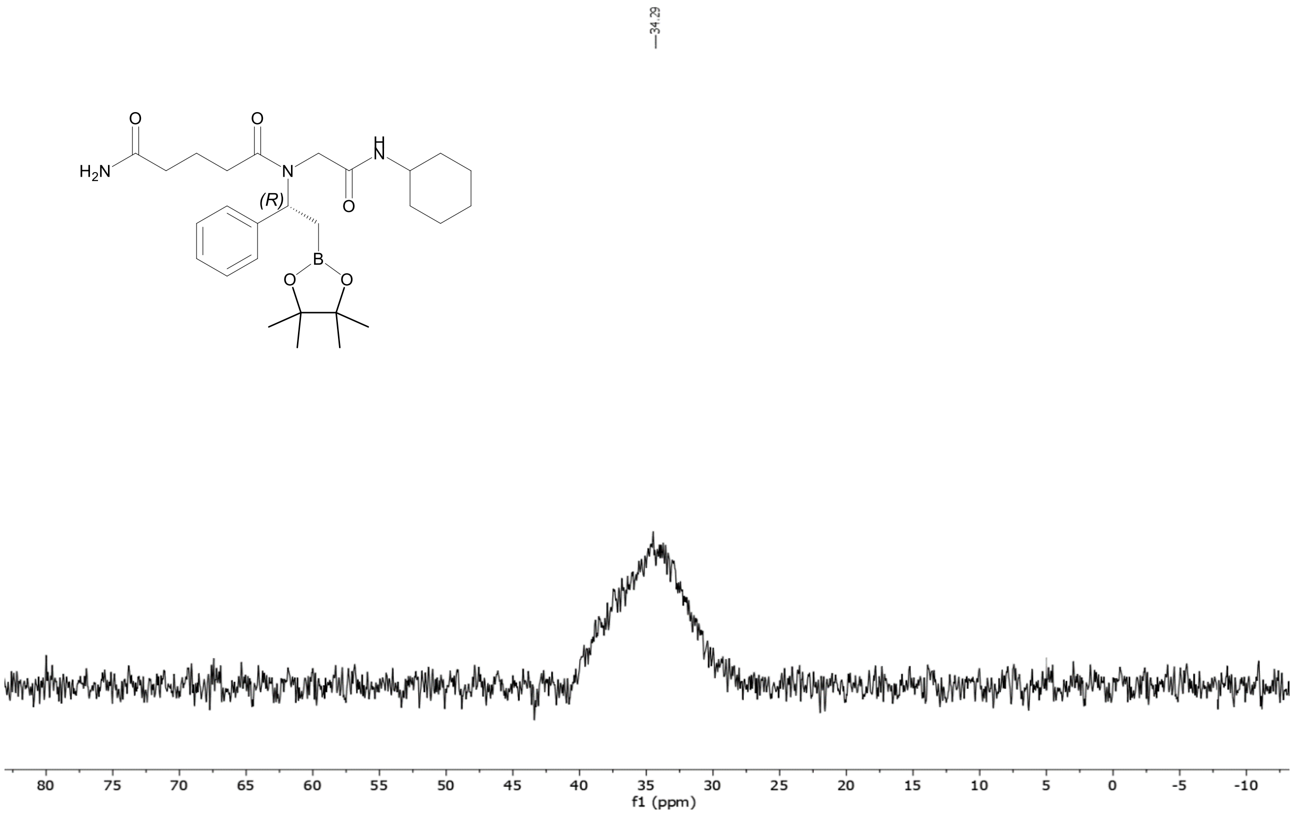

^{1}H NMR of compound **2h** (400 MHz, $CDCl_3$, 50:50 rotameric mixture)

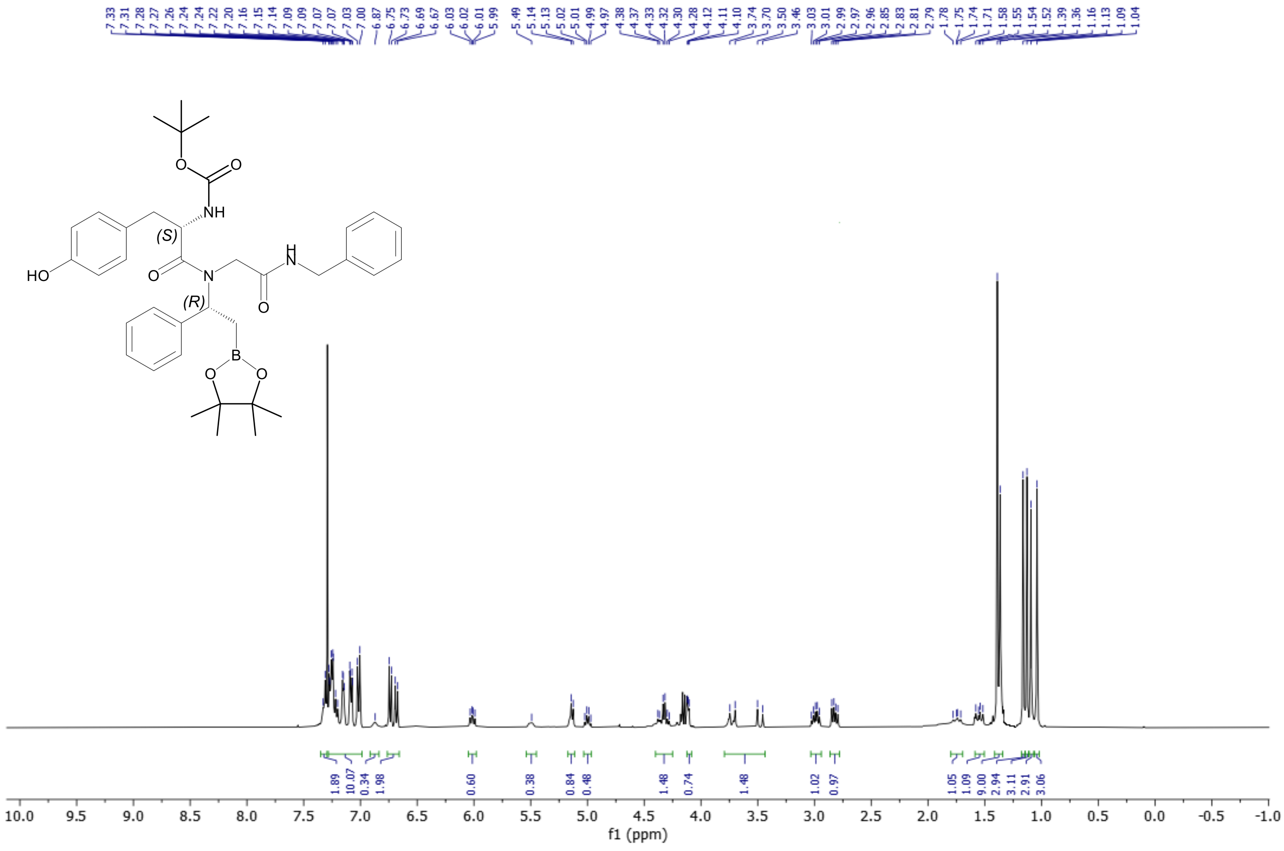

^{13}C NMR of compound **2h** (101 MHz, $CDCl_3$ 50:50 rotameric mixture)

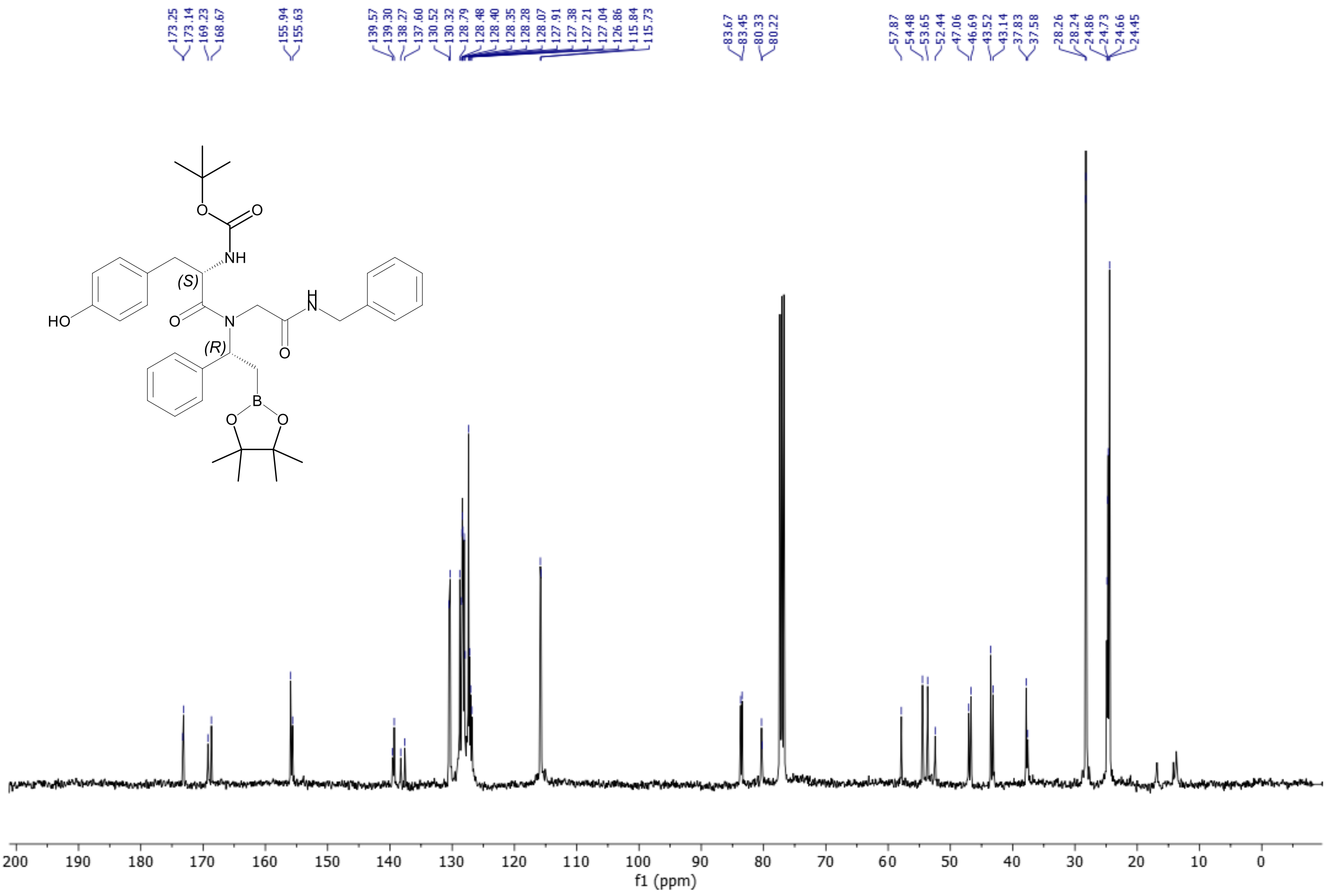

^{11}B NMR of compound **2h** (128 MHz, $CDCl_3$)

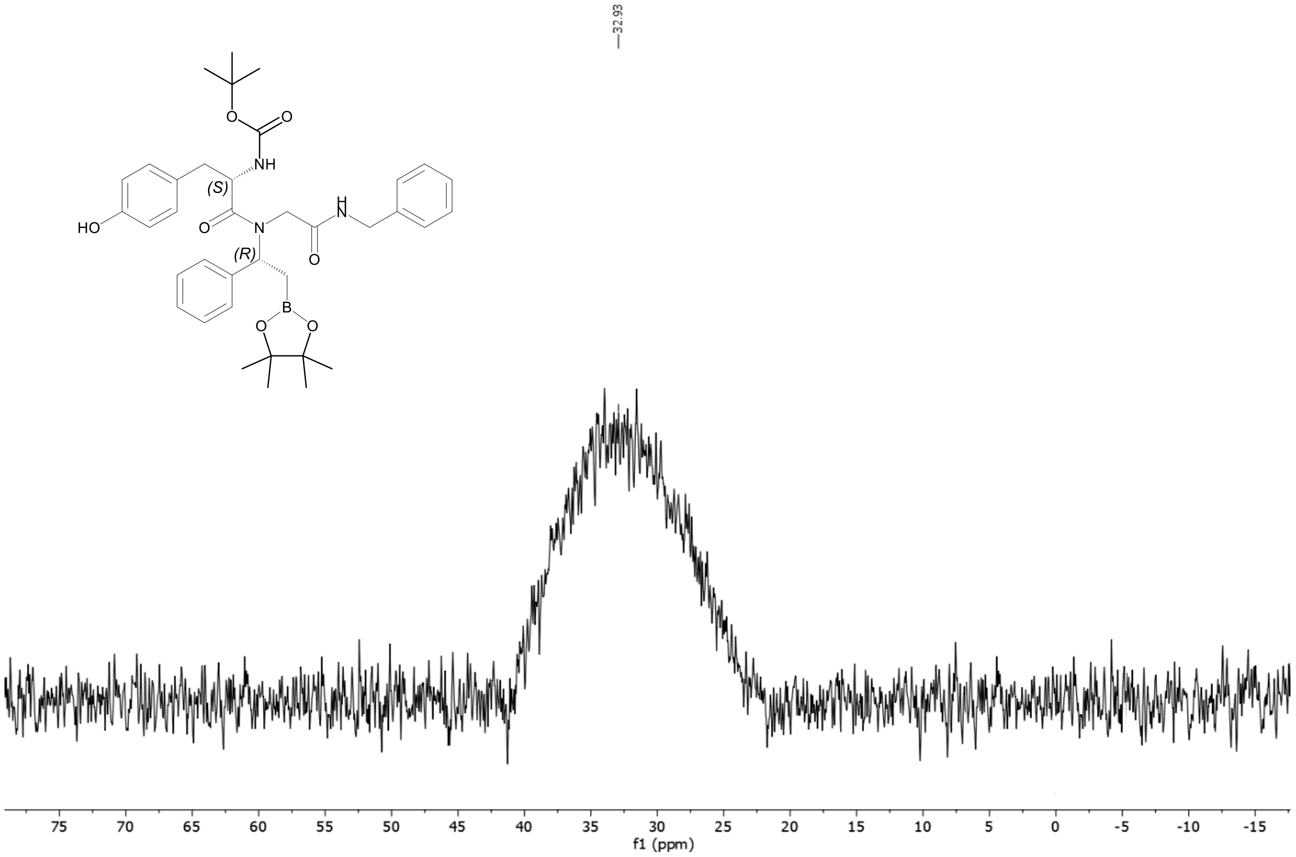

^{1}H NMR of compound **3a** (400 MHz, CD_3CN + 1 drop of H_2O, 60:40 rotameric mixture)

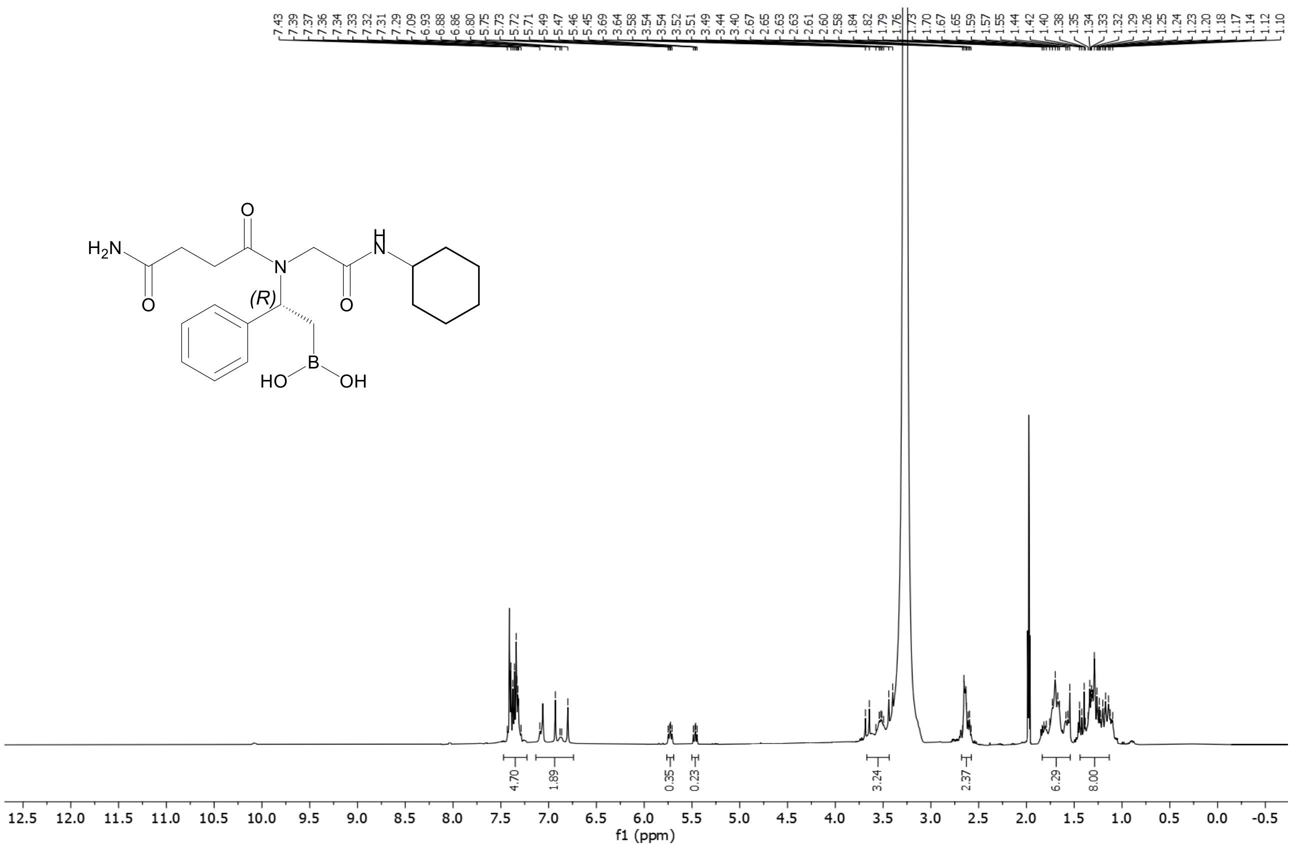

^{13}C NMR of compound **3a** (101 MHz, CD_3CN + 1 drop of H_2O, 60:40 rotameric mixture)

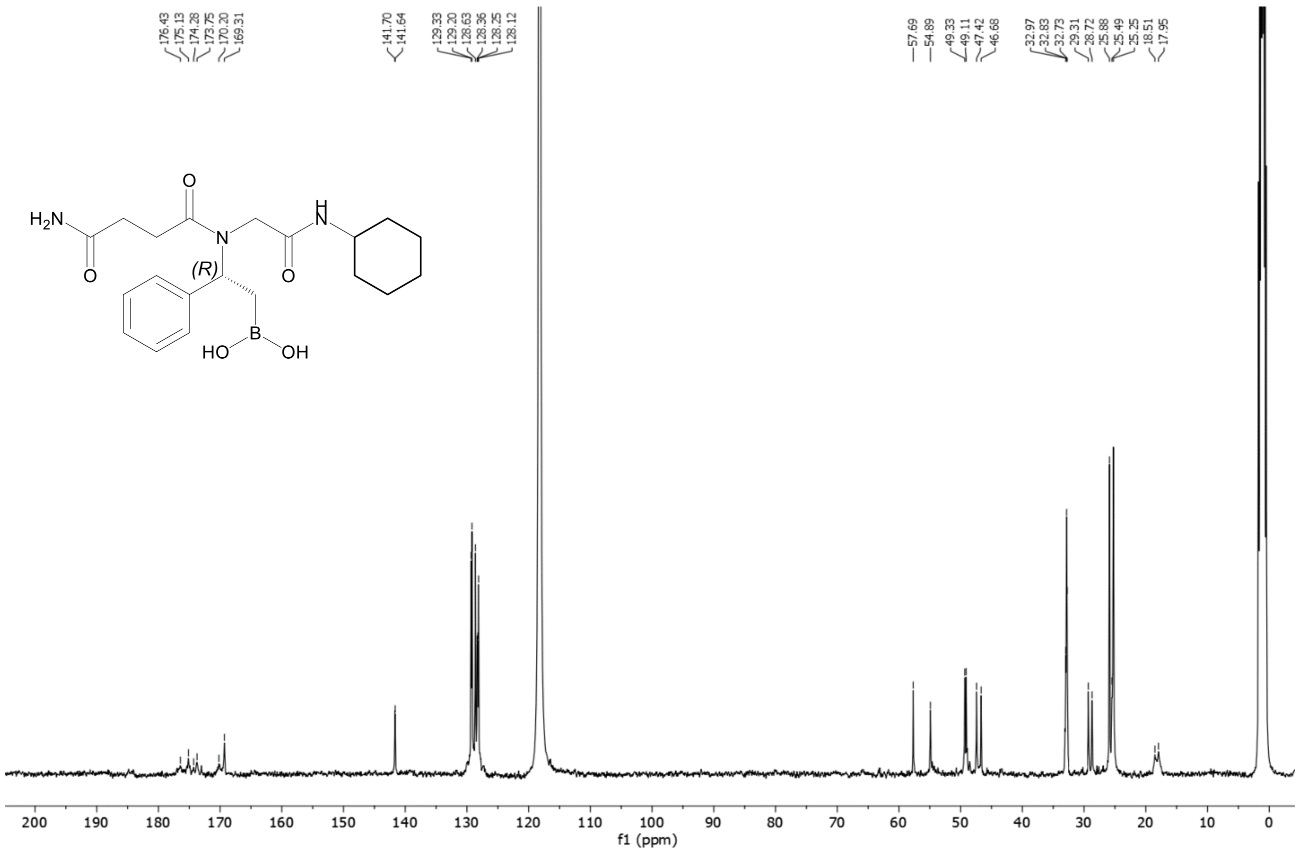

^{11}B NMR of compound **3a** (128 MHz, CD_3CN + 1 drop of H_2O)

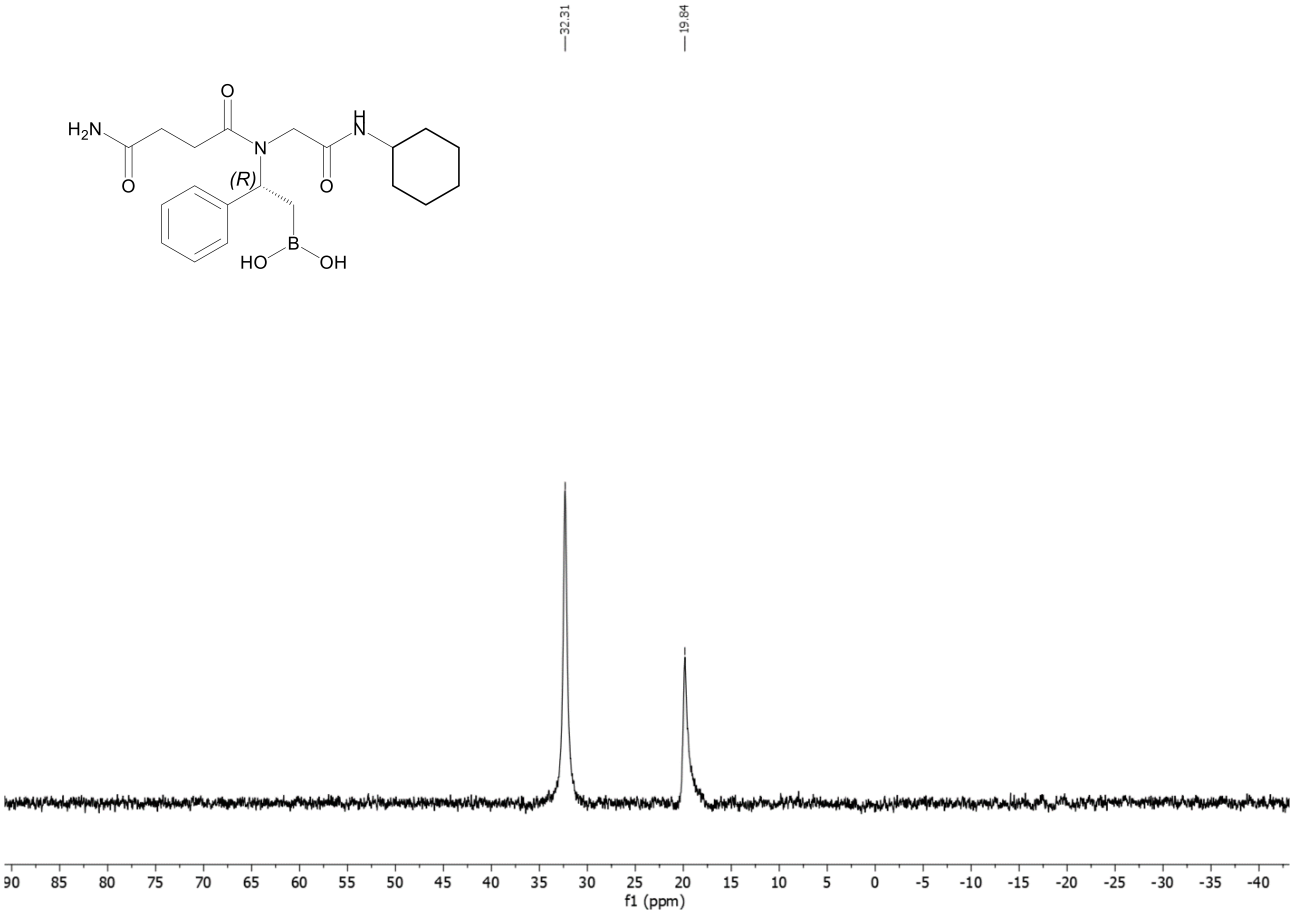

^{1}H NMR of compound **3b** (400 MHz, CD_3CN + 1 drop of H_2O, 70:30 rotameric mixture)

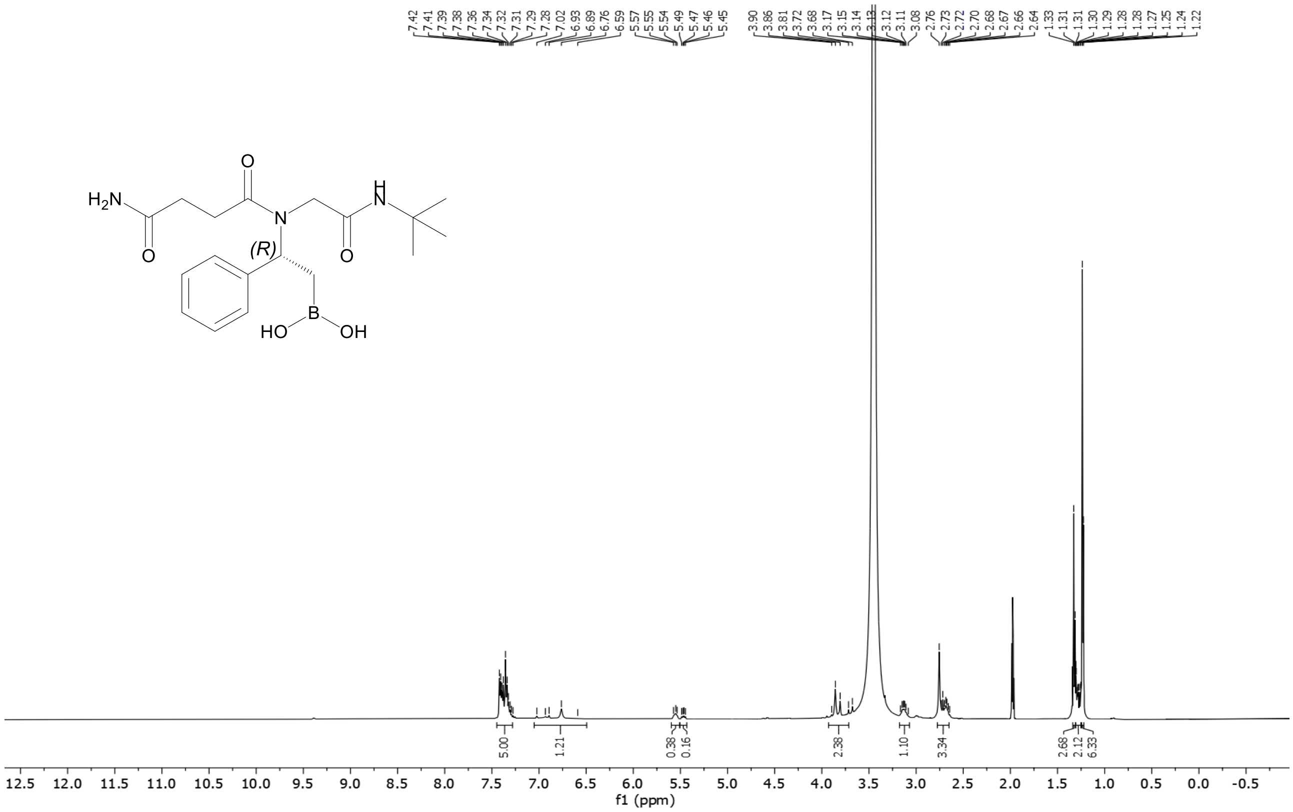

^{13}C NMR of compound **3b** (101 MHz, CD_3CN + 1 drop of H_2O, 70:30 rotameric mixture)

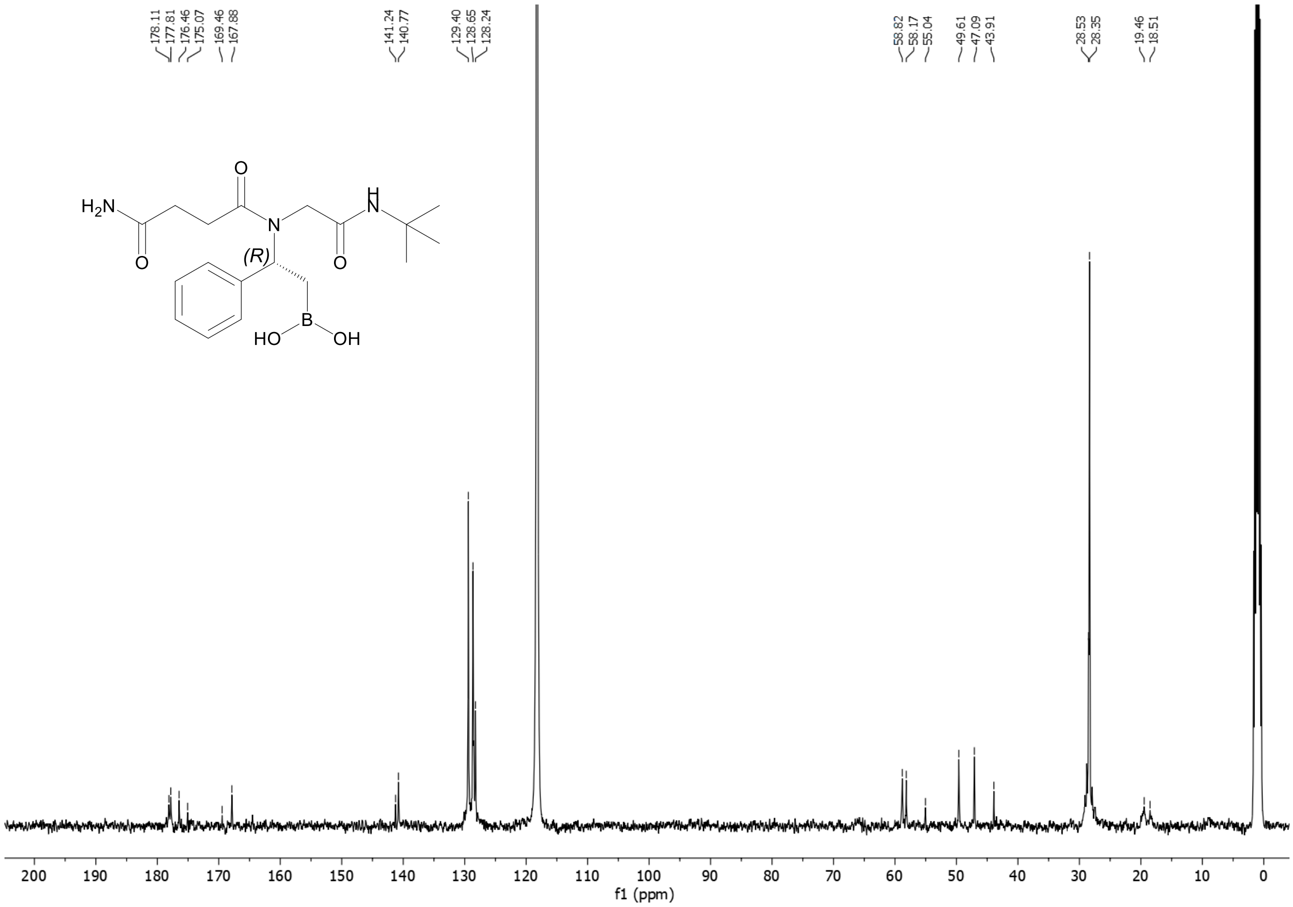

^{11}B NMR of compound **3b** (128 MHz, CD_3CN + 1 drop of H_2O)

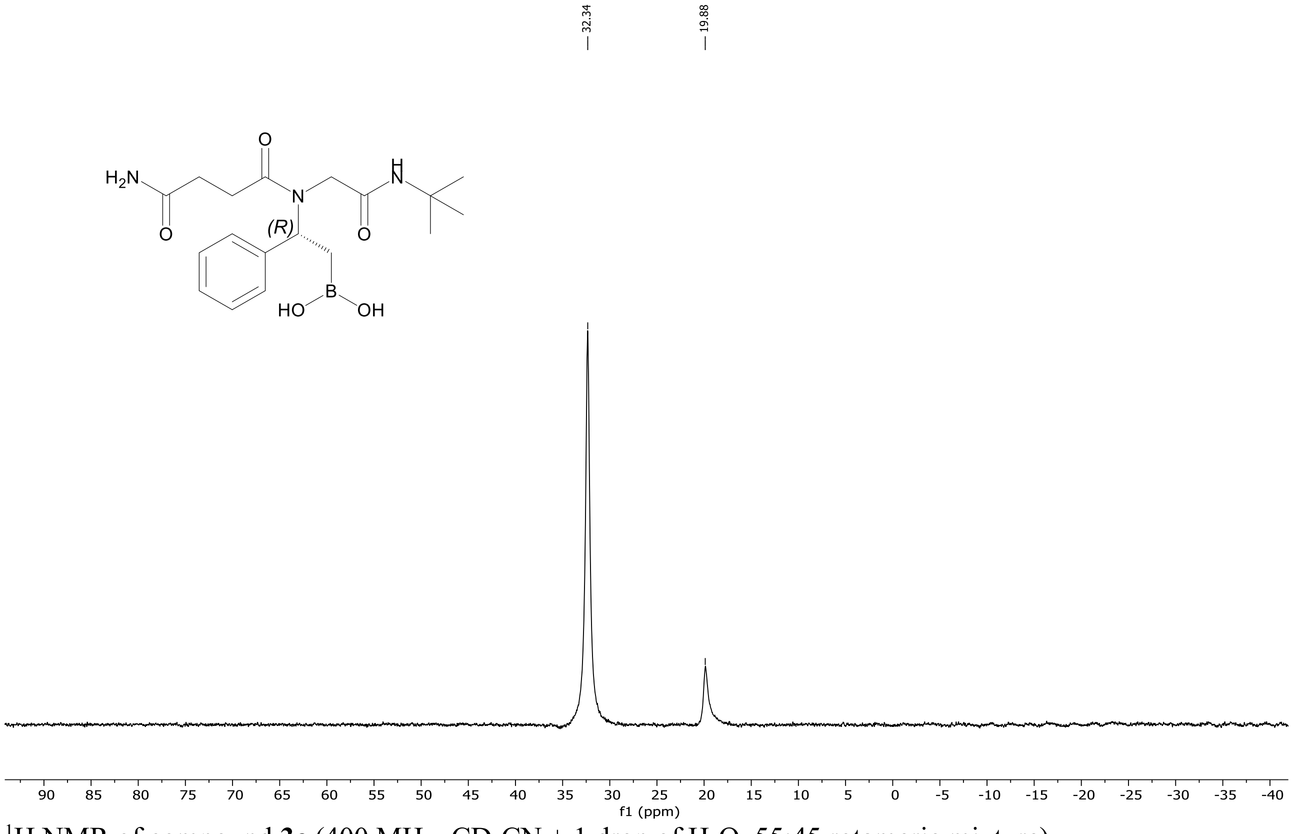

^{1}H NMR of compound **3c** (400 MHz, CD_3CN + 1 drop of H_2O, 55:45 rotameric mixture)

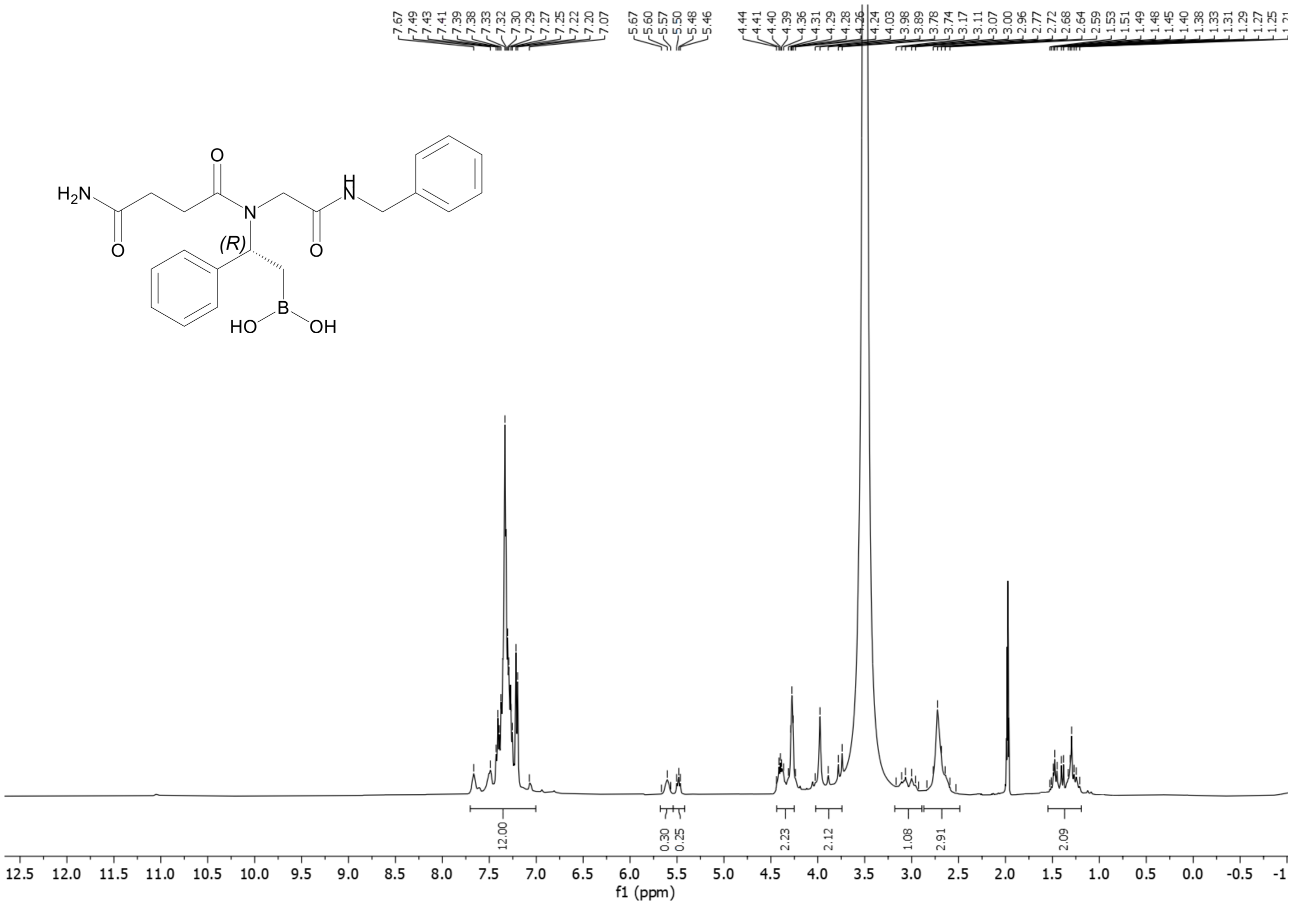

^{13}C NMR of compound **3c** (101 MHz, CD_3CN + 1 drop of H_2O, 55:45 rotameric mixture)

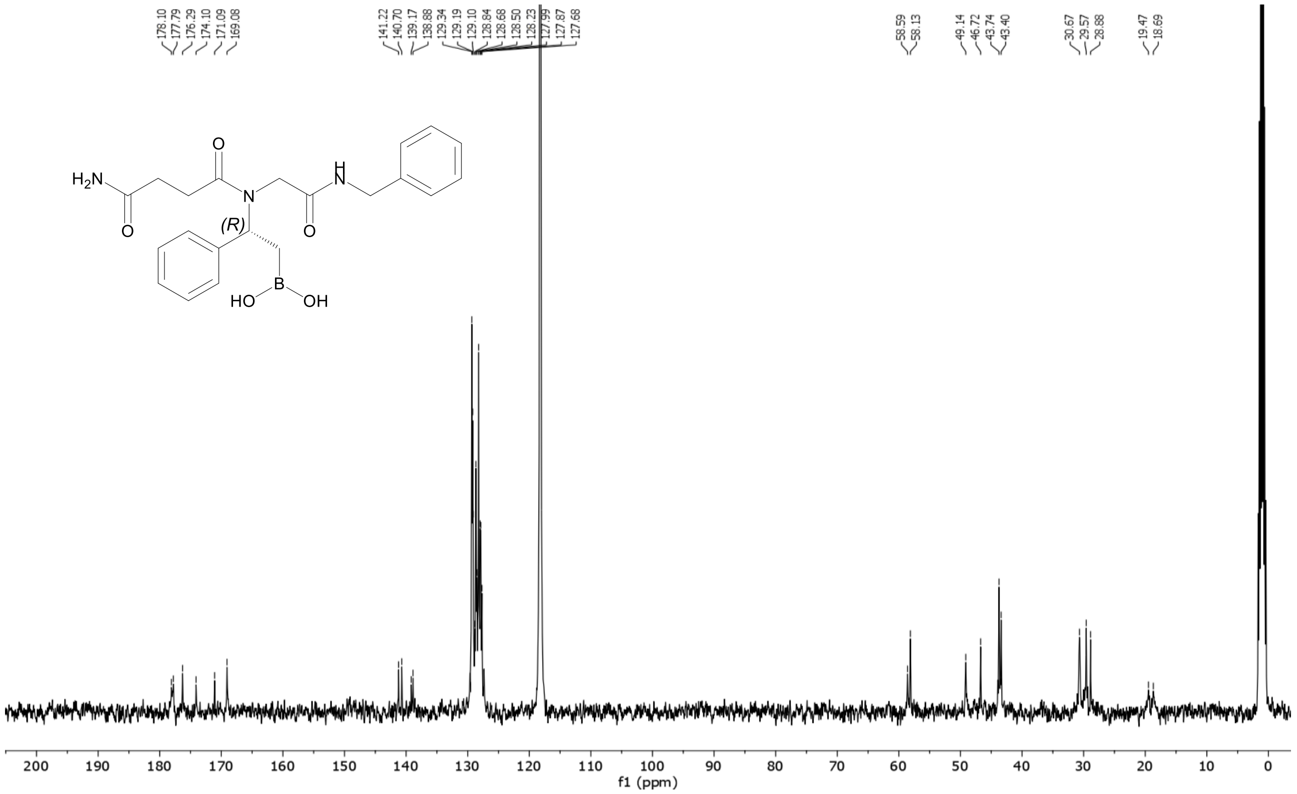

^{11}B NMR of compound **3c** (128 MHz, CD_3CN + 1 drop of H_2O)

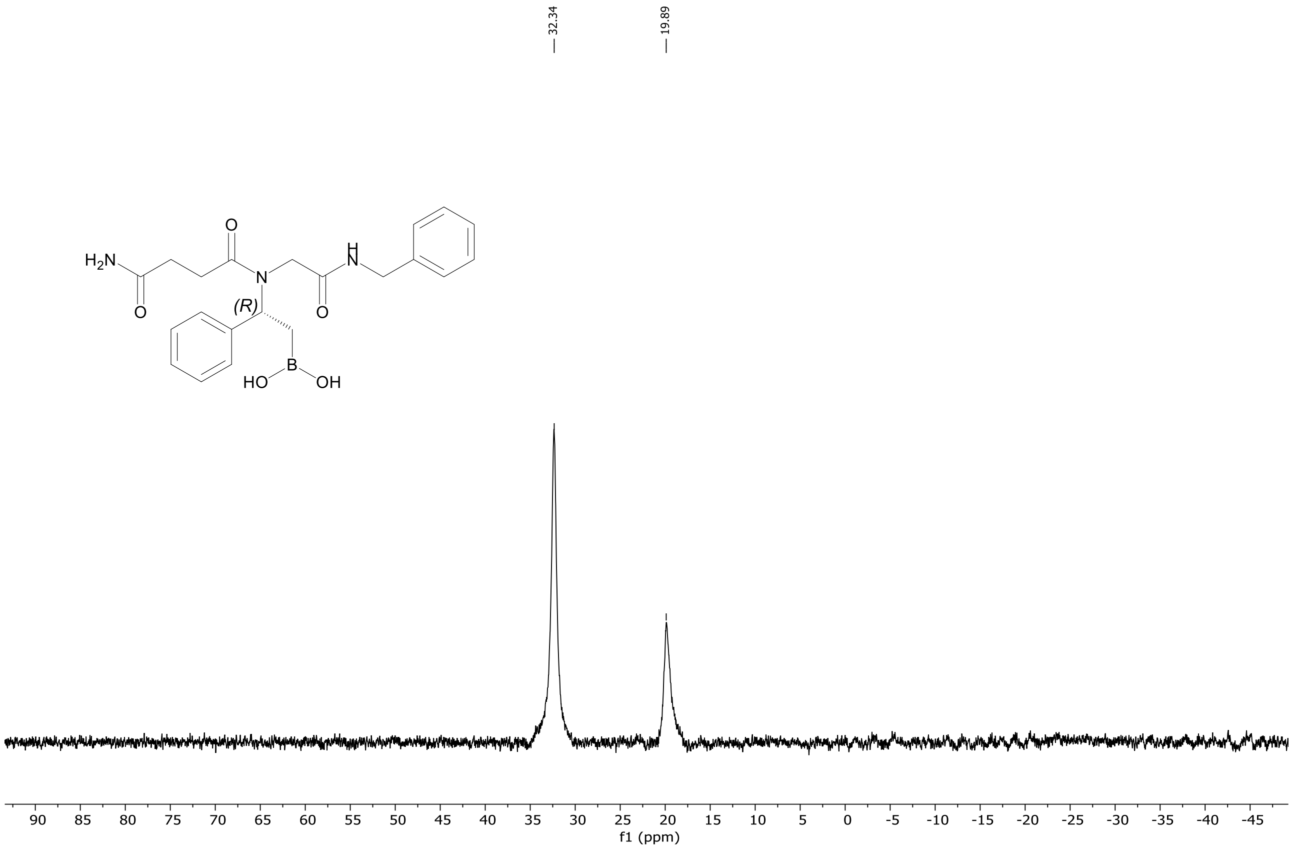

^{1}H NMR of compound **3d** (400 MHz, $CDCl_3$, complex rotameric mixture: the section by section integration proves the overall number of protons)

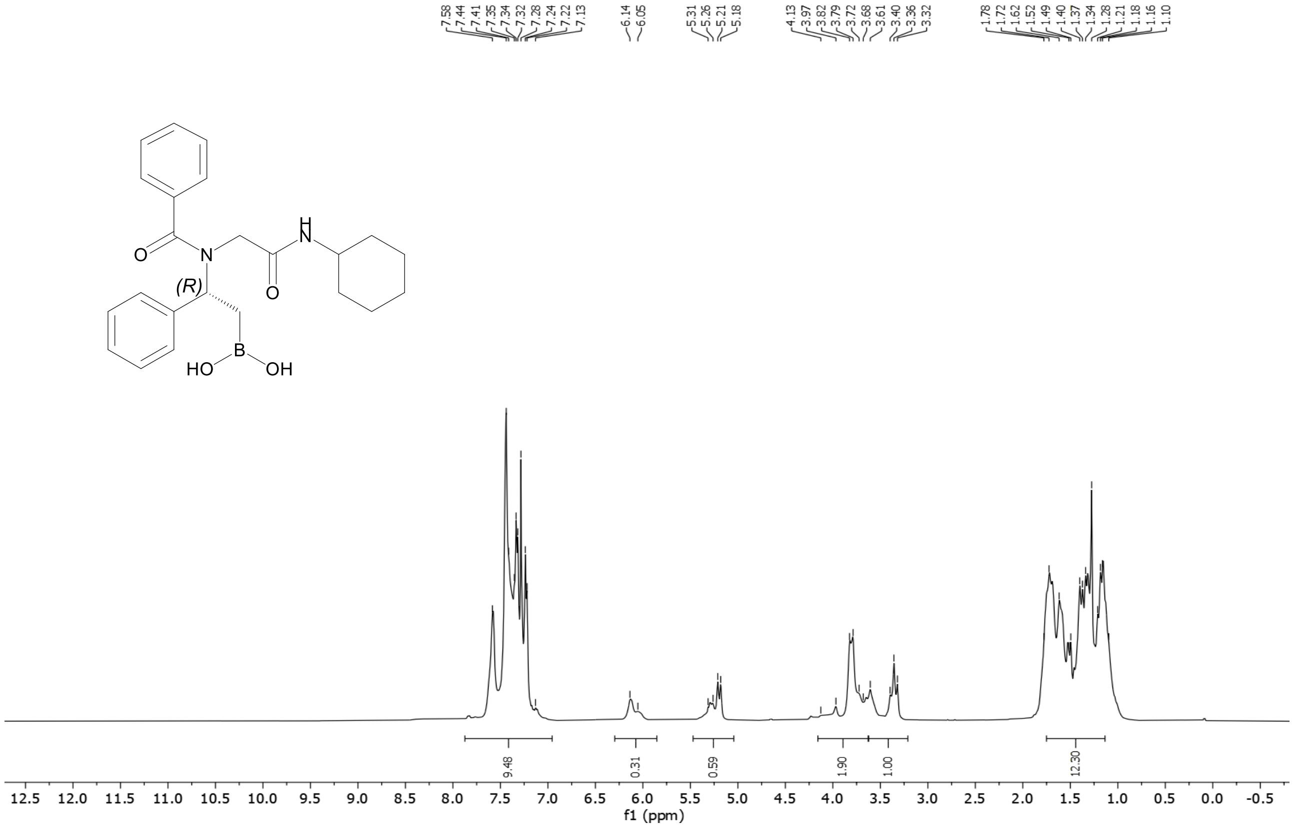

^{13}C NMR of compound **3d** (101 MHz, $CDCl_3$, complex rotameric mixture)

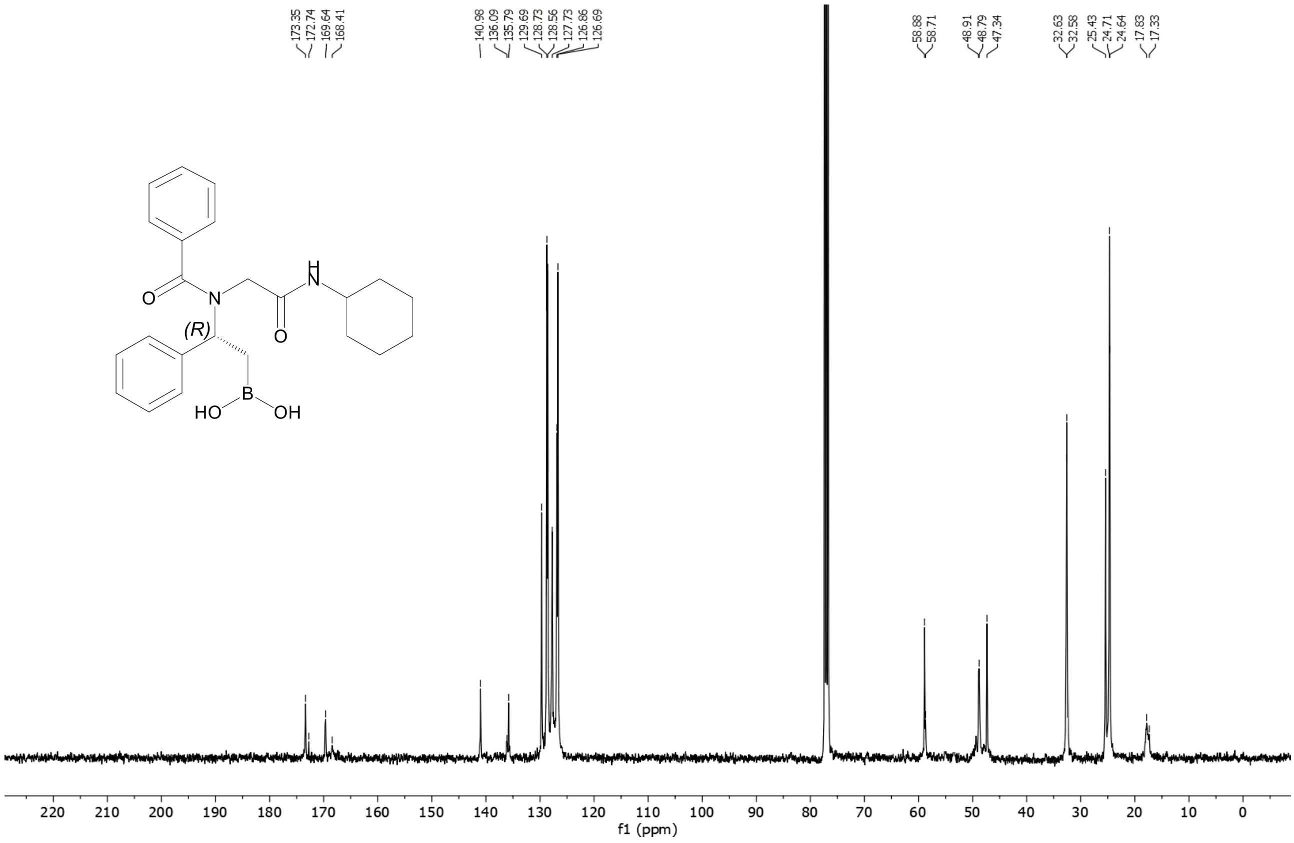

^{11}B NMR of compound **3d** (128 MHz, $CDCl_3$)

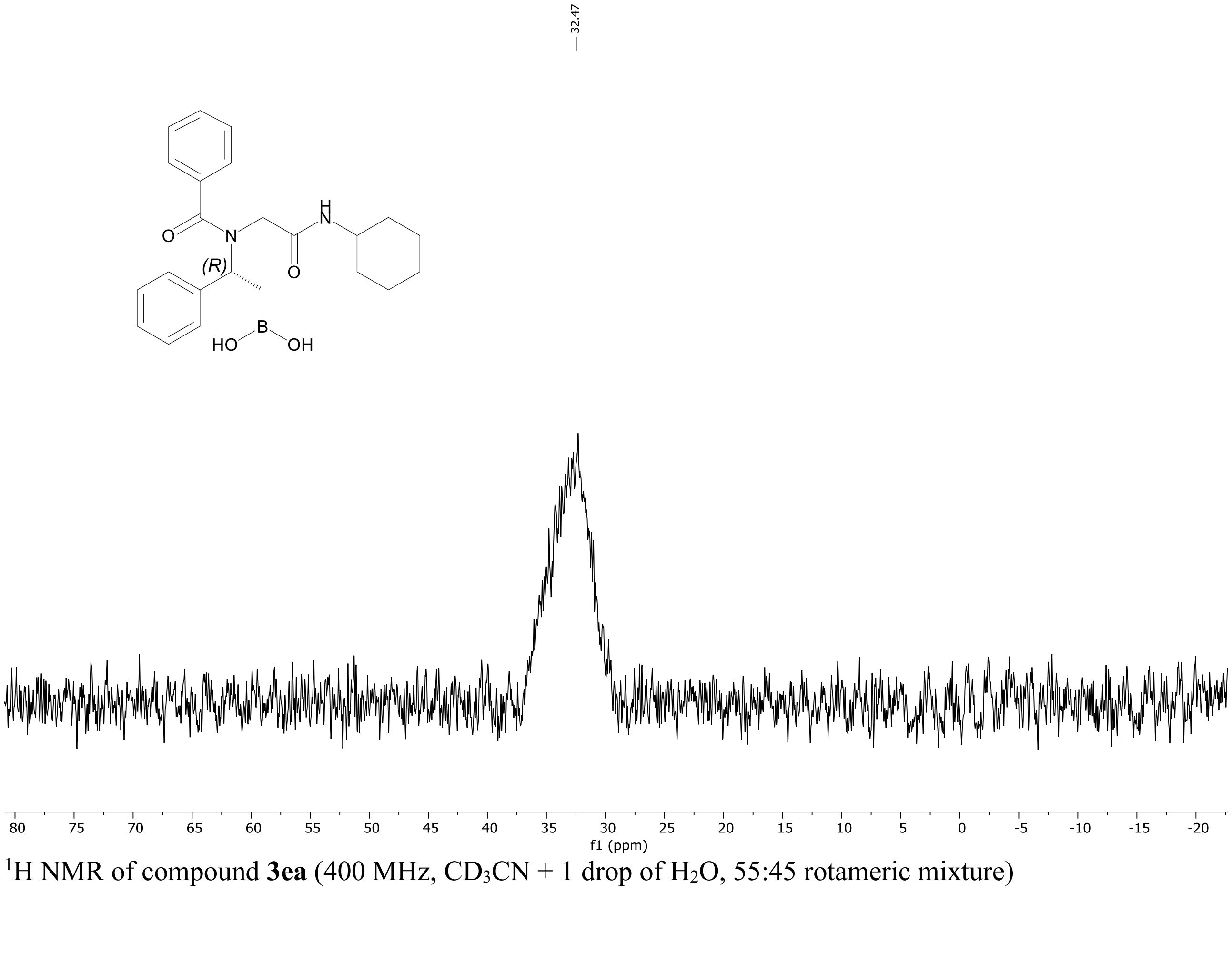

^{1}H NMR of compound **3ea** (400 MHz, CD_3CN + 1 drop of H_2O, 55:45 rotameric mixture)

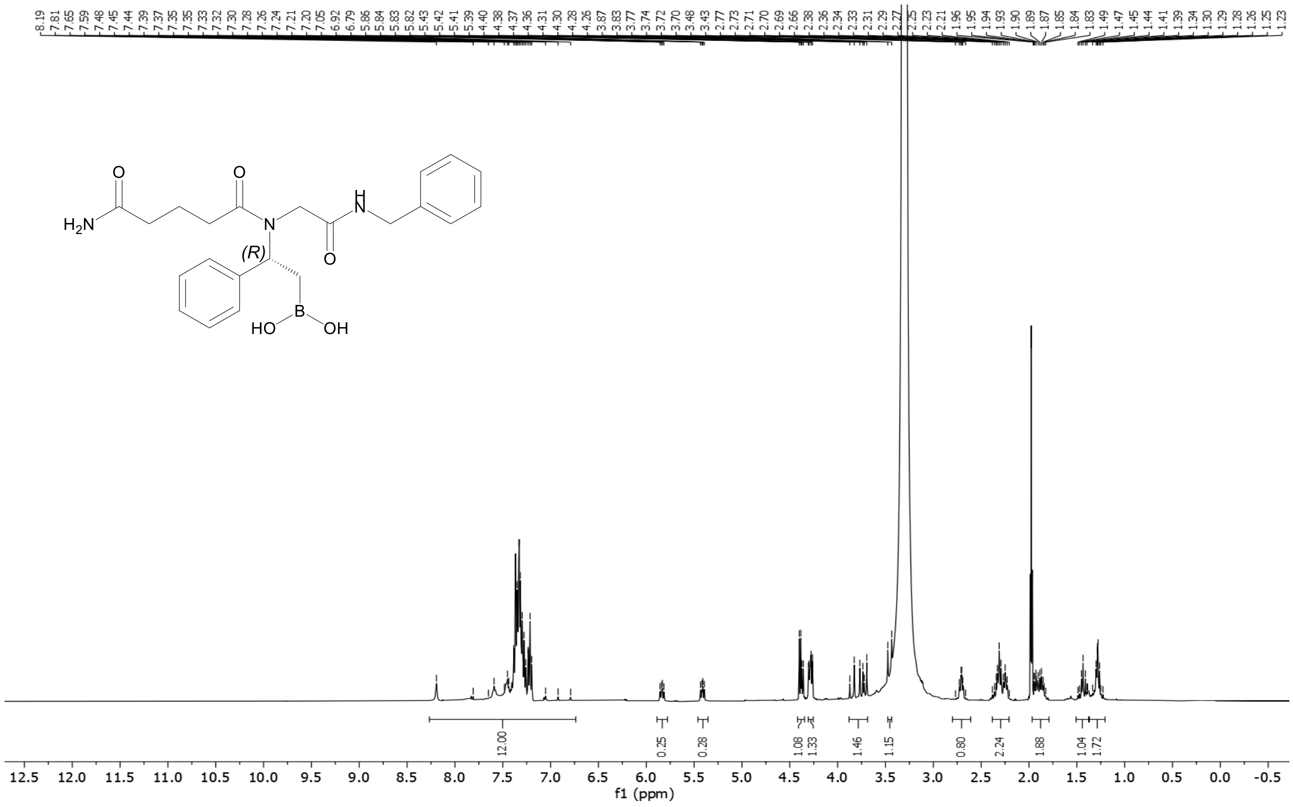

^{13}C NMR of compound **3ea** (101 MHz, CD_3CN + 1 drop of H_2O, 55:45 rotameric mixture)

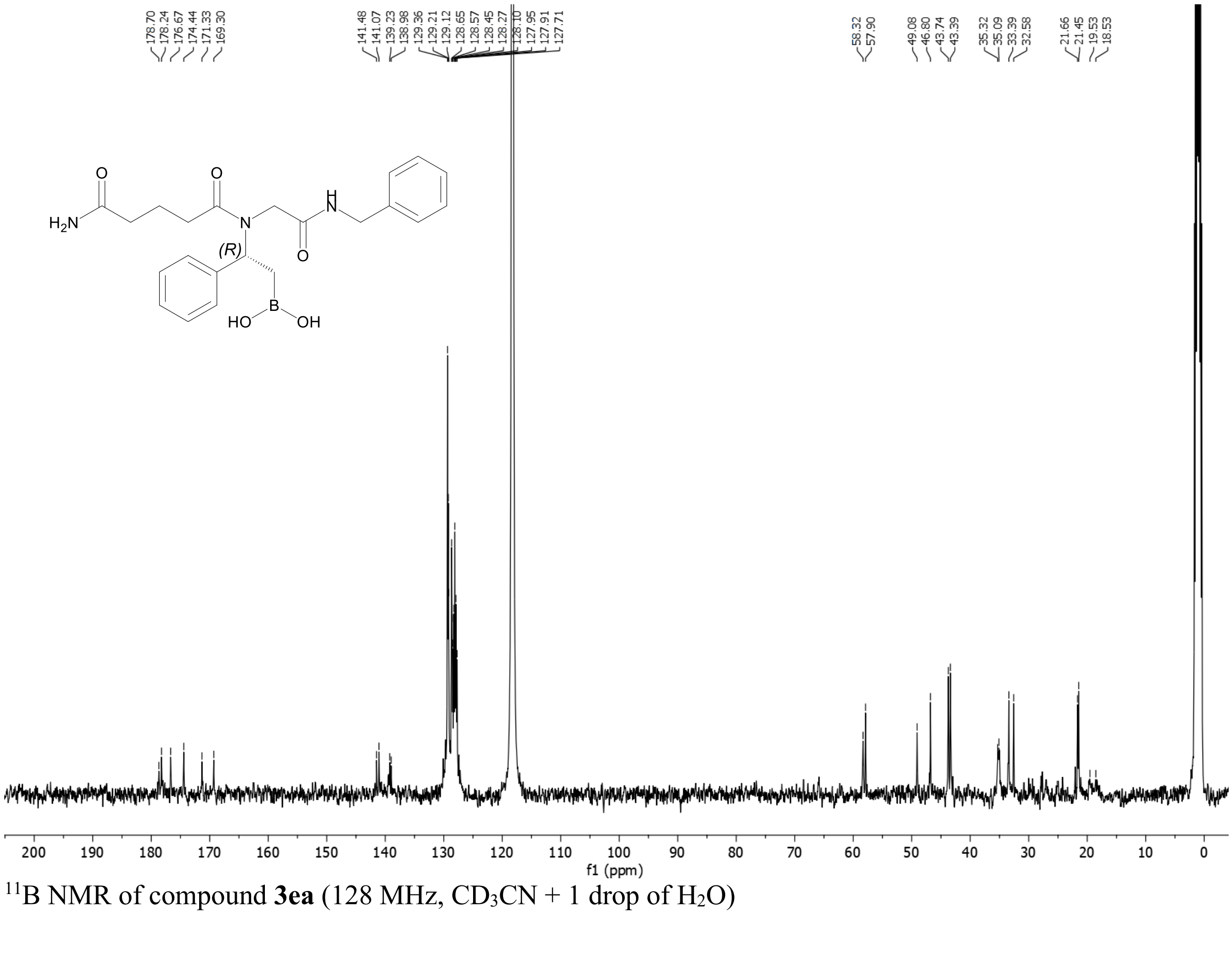

^{11}B NMR of compound **3ea** (128 MHz, CD_3CN + 1 drop of H_2O)

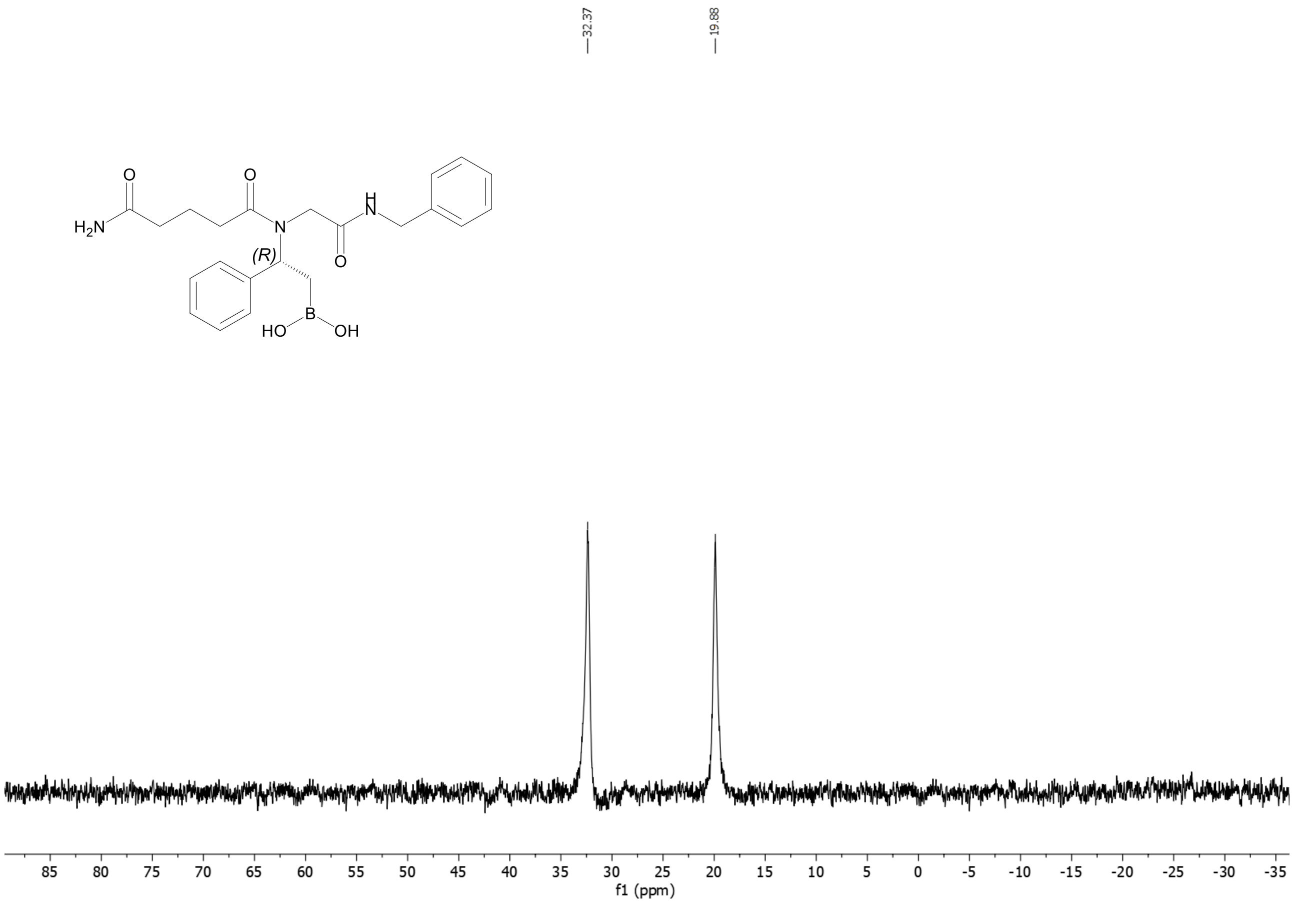

^{1}H NMR of compound **3f** (400 MHz, CD_3CN + 1 drop of H_2O, 65:35 rotameric mixture)

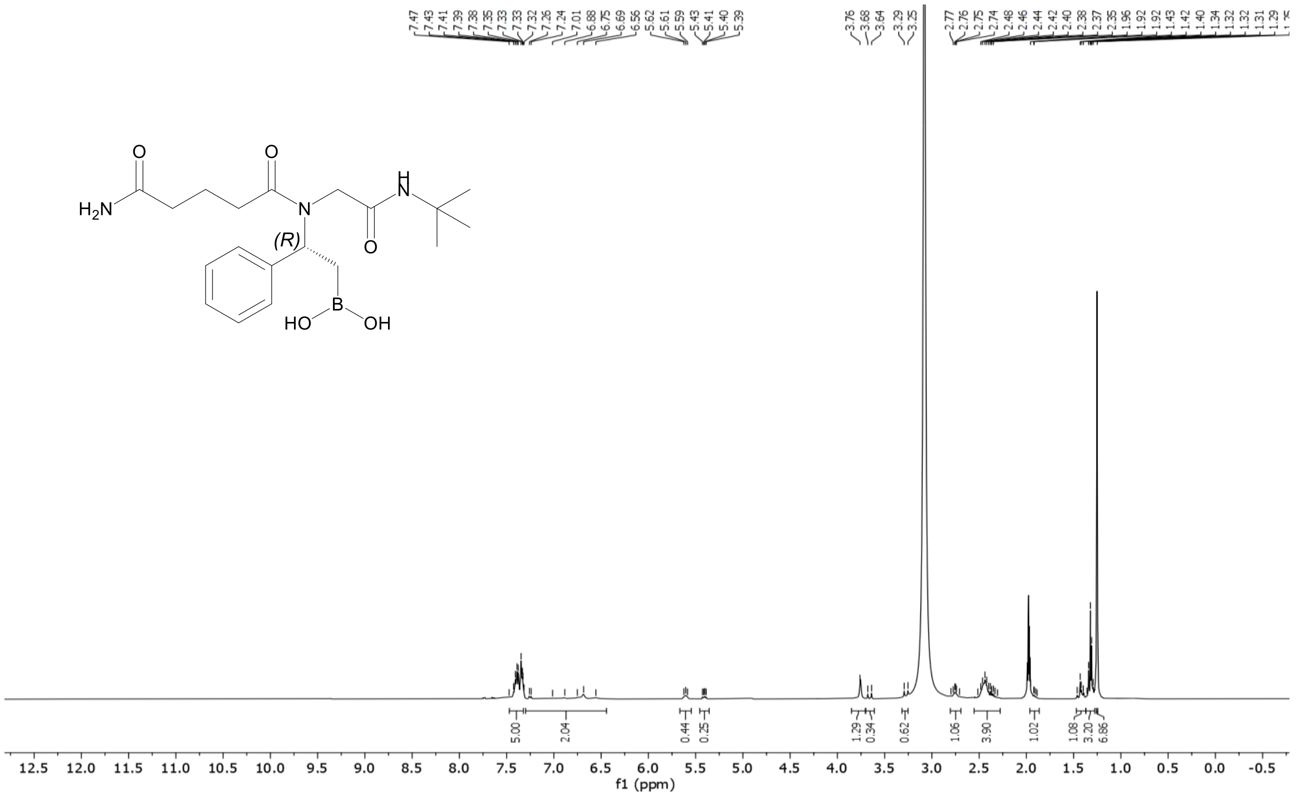

^{13}C NMR of compound **3f** (101 MHz, CD_3CN + 1 drop of H_2O, 65:35 rotameric mixture)

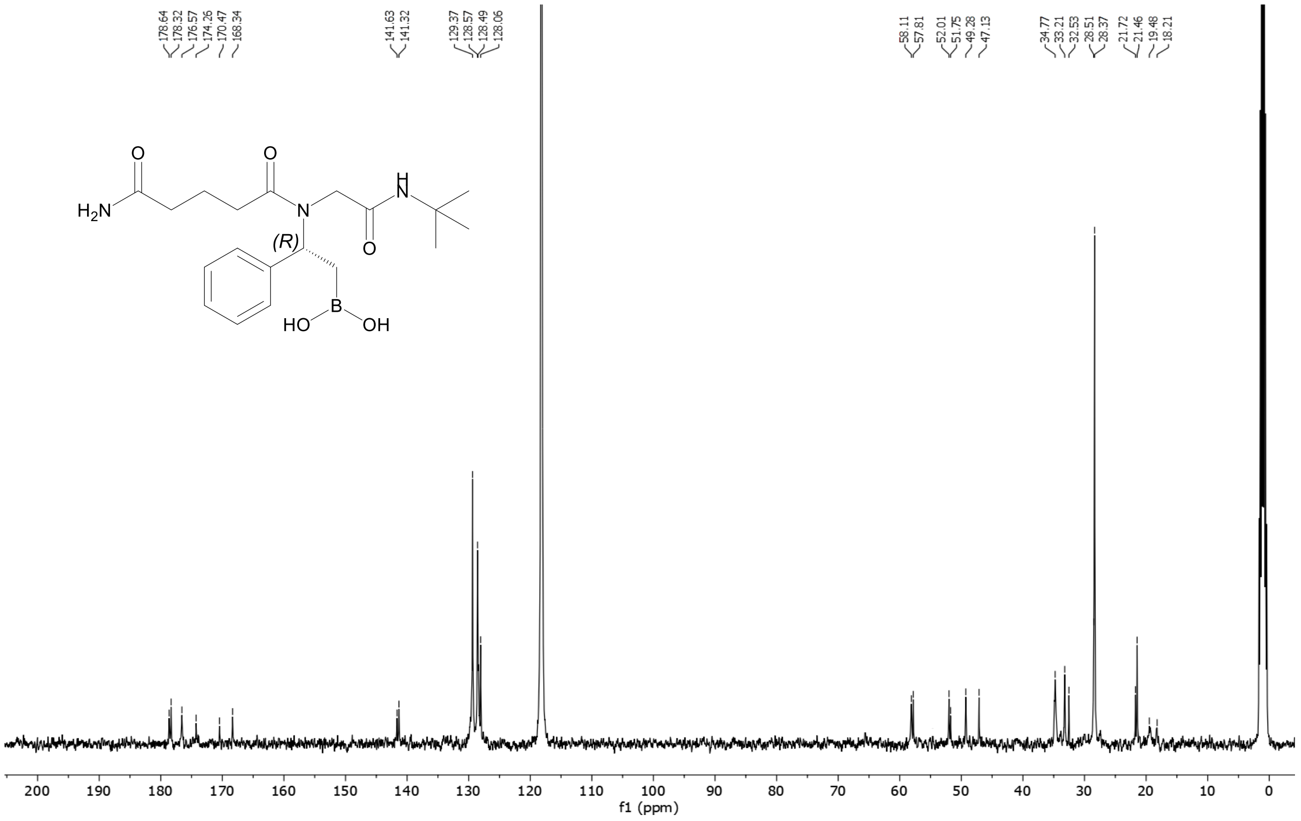

^{11}B NMR of compound **3f** (128 MHz, CD_3CN + 1 drop of H_2O)

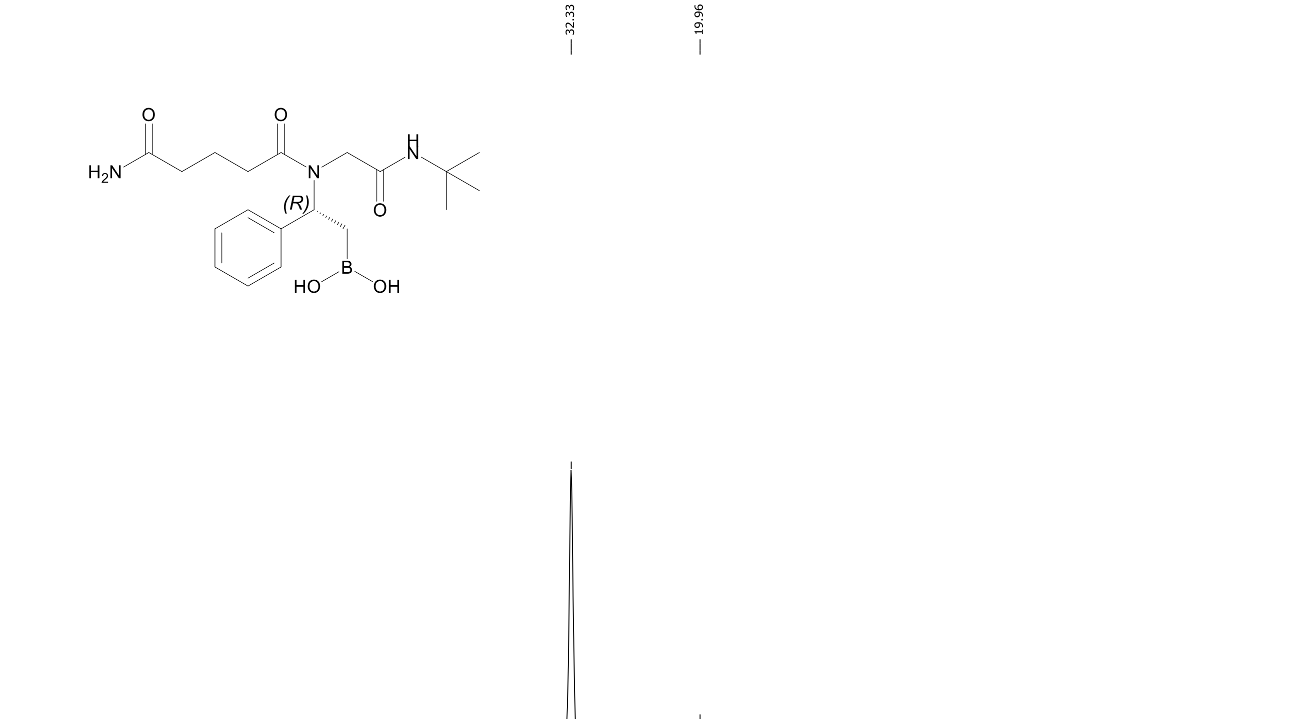

^{1}H NMR of compound **3g** (400 MHz, CD_3CN + 1 drop of H_2O, 60:40 rotameric mixture)

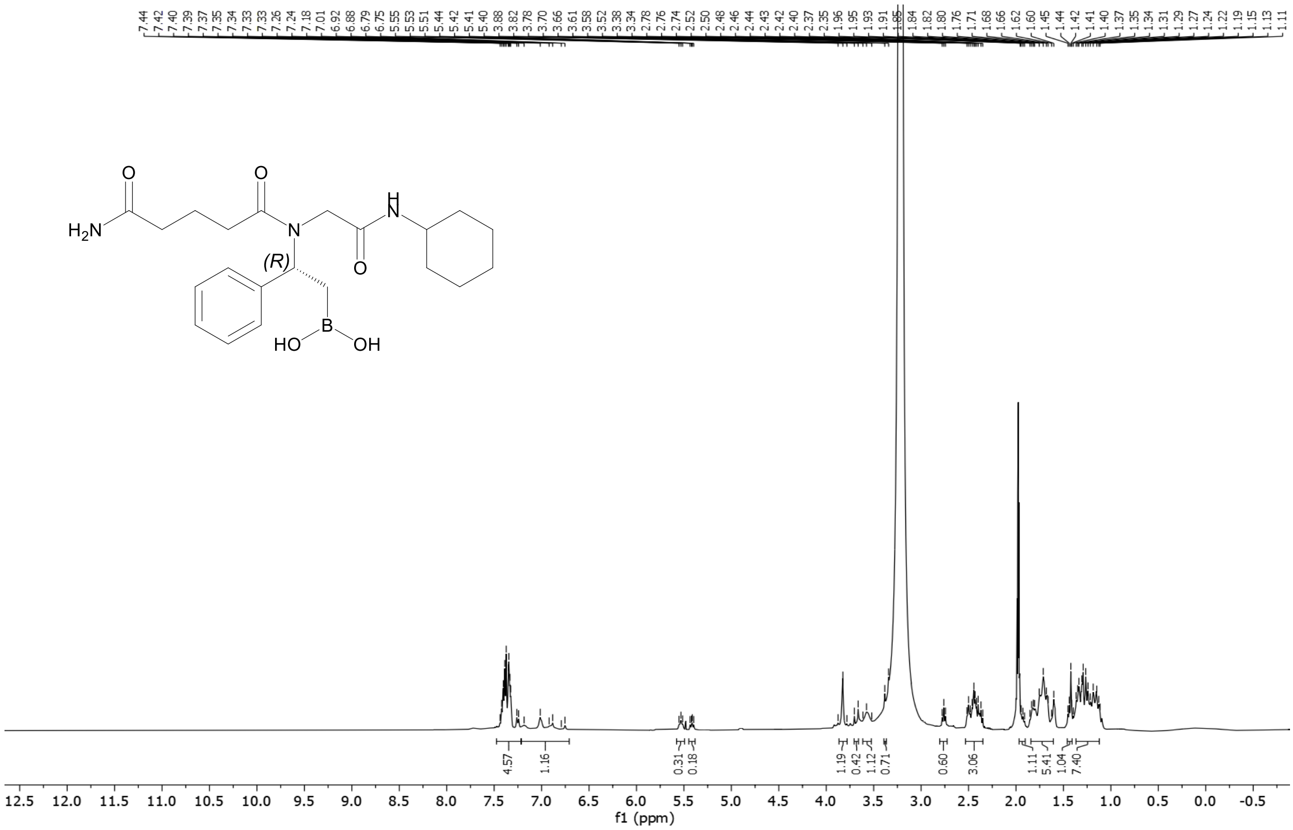

^{13}C NMR of compound **3g** (101 MHz, CD_3CN + 1 drop of H_2O, 60:40 rotameric mixture)

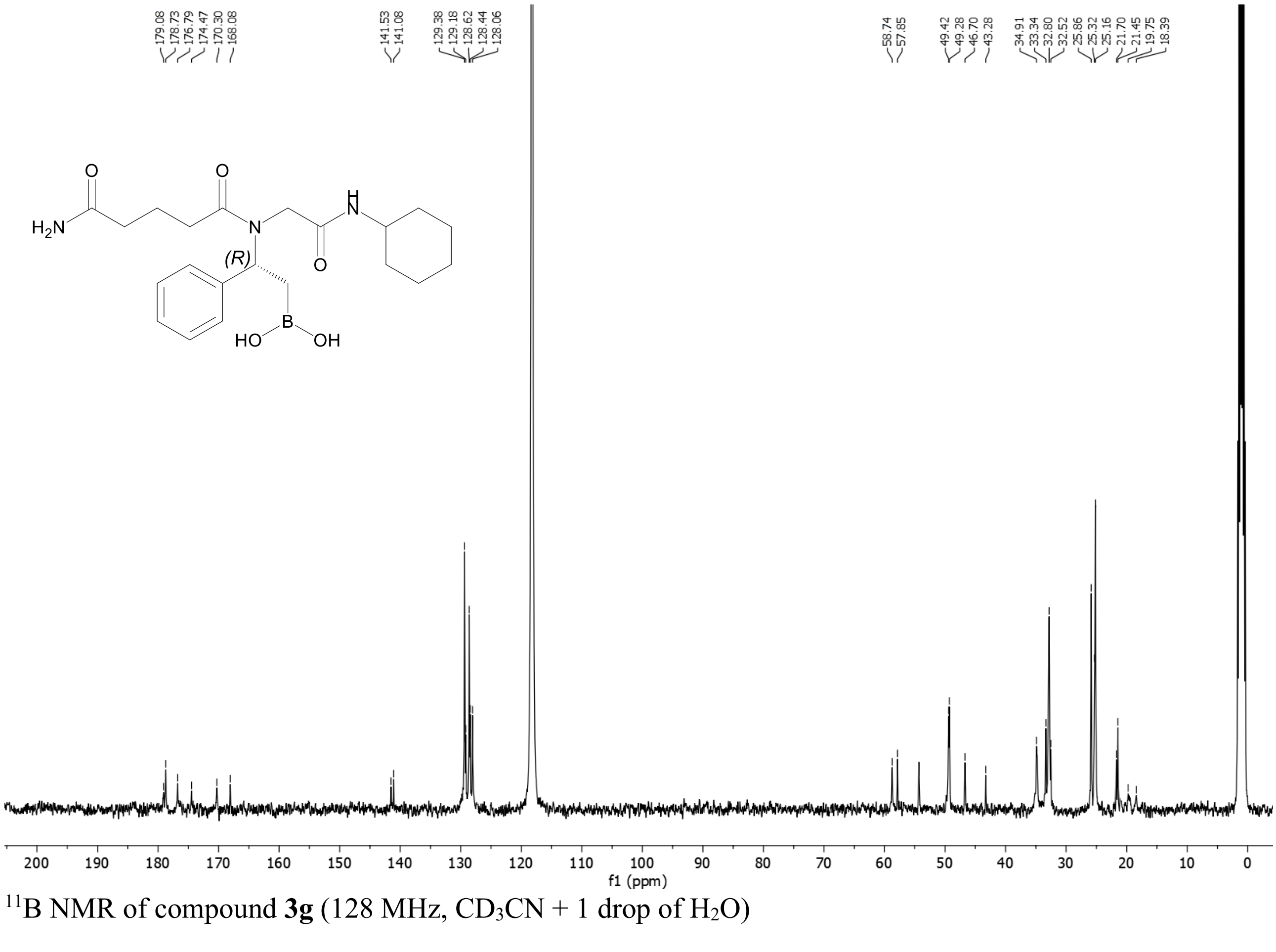

^{11}B NMR of compound **3g** (128 MHz, CD_3CN + 1 drop of H_2O)

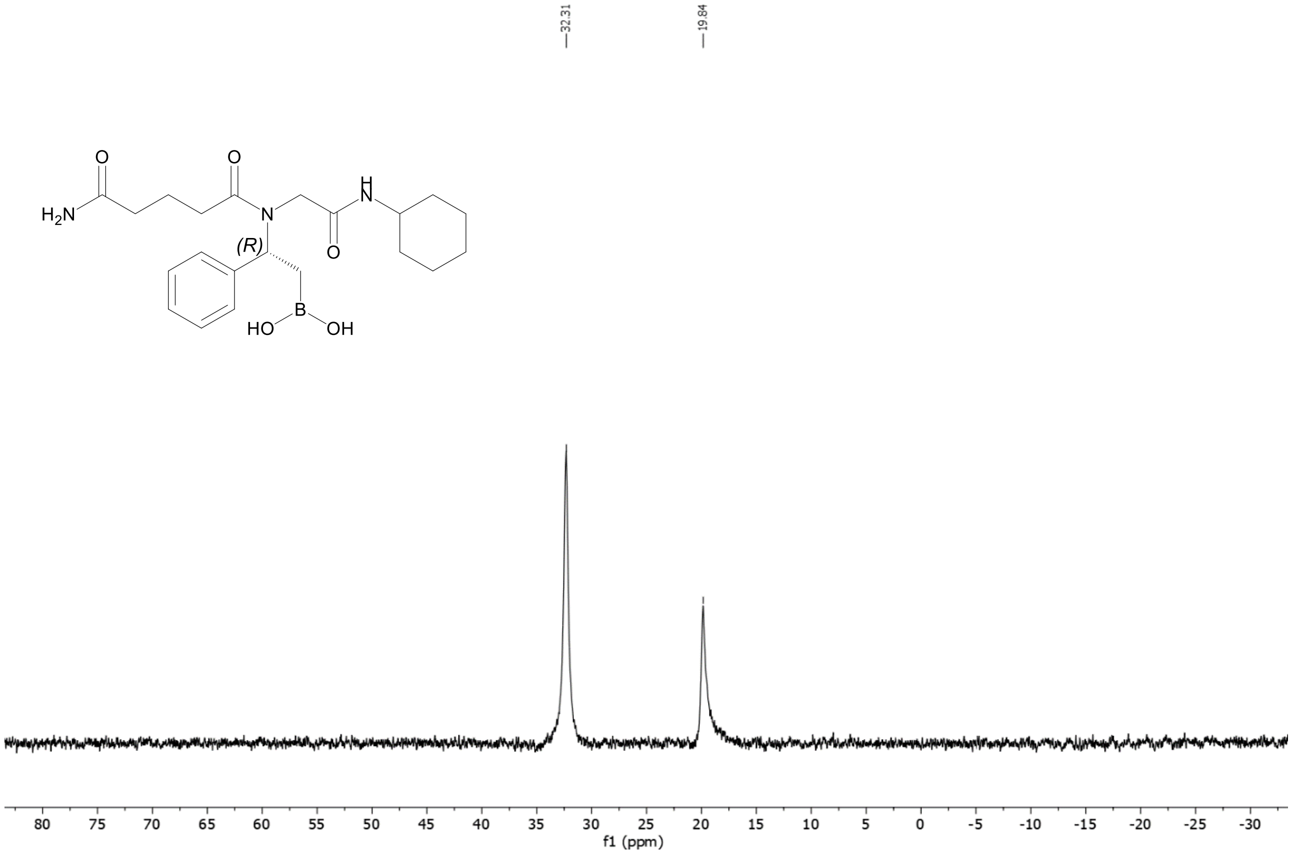

^{1}H NMR of compound **3h** (400 MHZ, CD_3CN + 1 drop of H_2O, 60:40 rotameric mixture)

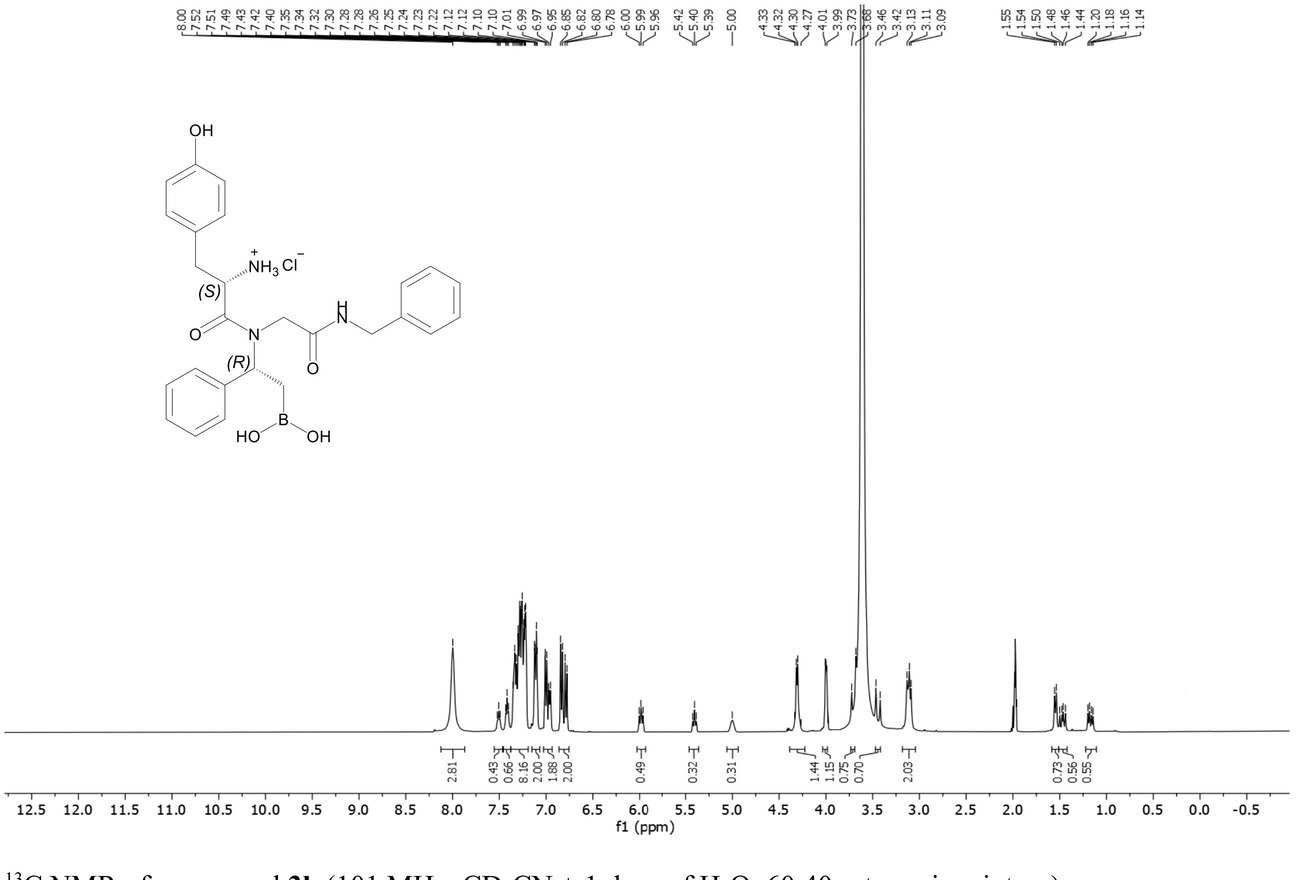

^{13}C NMR of compound **3h** (101 MHz, CD_3CN + 1 drop of H_2O, 60:40 rotameric mixture)

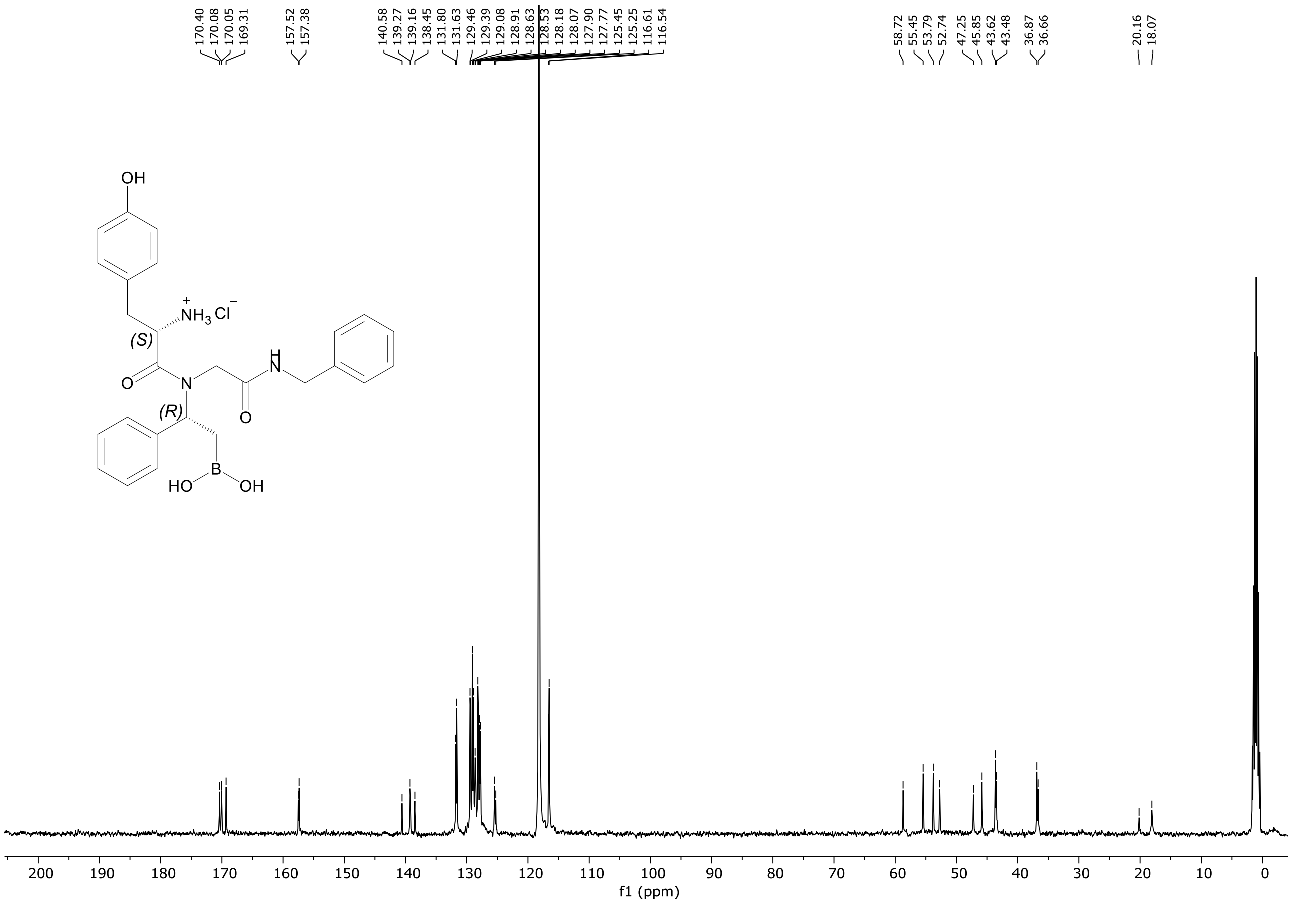

^{11}B NMR of compound **3h** (128 MHz, CD_3CN + 1 drop of H_2O)

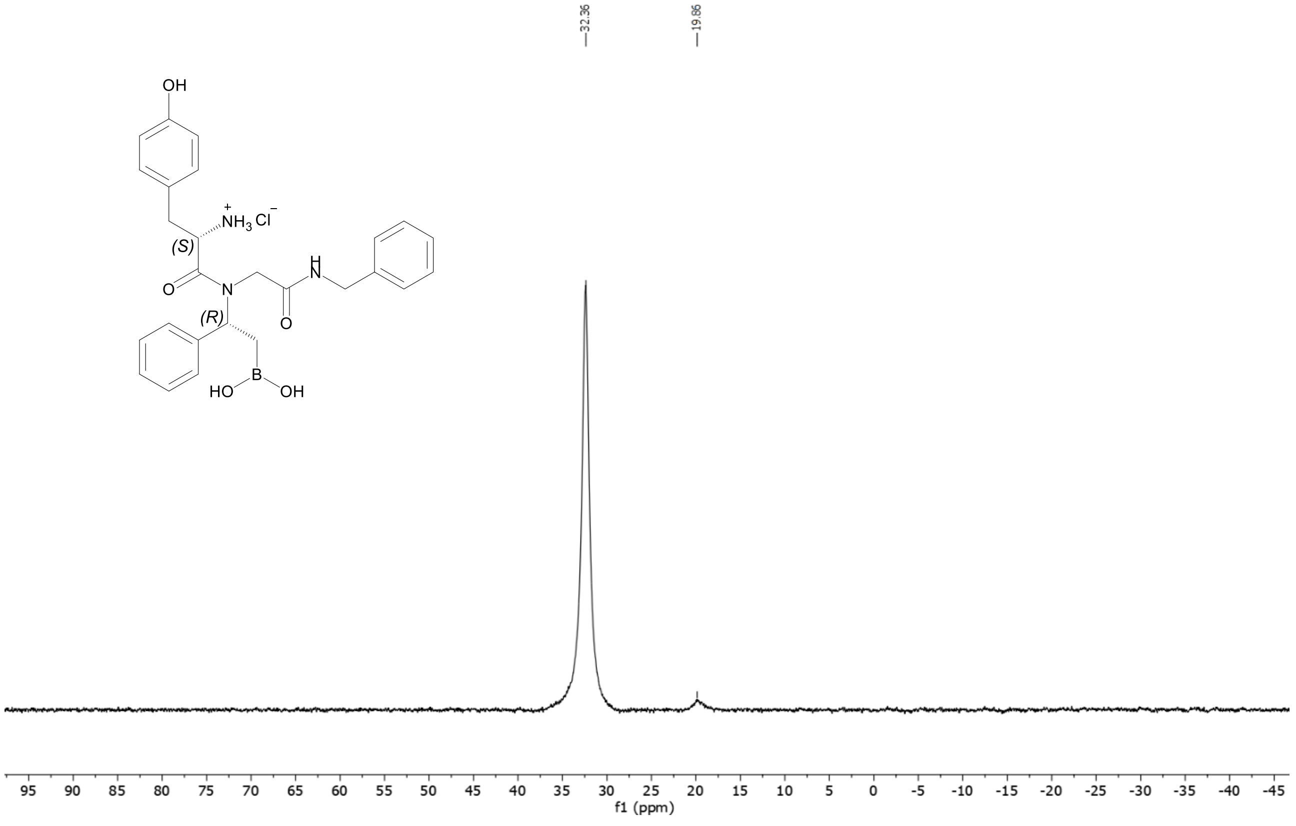

Supplement: Supplementary file 1 [file molecules-28-02356-s001.zip › molecules-2222639-supplementary.pdf]
